# Advancing infection therapy: the role of novel menthol-based antimicrobials

**DOI:** 10.1080/14756366.2025.2596488

**Published:** 2026-01-06

**Authors:** Annalisa Di Rienzo, Abdelmoujoud Faris, Marina Mingoia, Carmela Conte, Lorella Marinucci, Gloria Magi, Maria Concetta Cufaro, Piero Del Boccio, Marco Maioli, Antonio Di Stefano, Ivana Cacciatore

**Affiliations:** ^a^ Department of Pharmacy, University “G. d’Annunzio” of Chieti-Pescara, Chieti Scalo, Italy; ^b^ LIMAS, Department of Chemical Sciences, Faculty of Sciences Dhar El Mahraz, Sidi Mohamed Ben Abdellah University, Fez, Morocco; ^c^ Department of Biomedical Sciences and Public Health, Medical School, Polytechnic University of Marche, Ancona, Italy; ^d^ Department of Pharmaceutical Sciences, University of Perugia, Perugia, Italy; ^e^ Department of Medicine and Surgery, University of Perugia, Perugia, Italy; ^f^ Center for Advanced Studies and Technology (CAST), University “G. d’Annunzio” of Chieti-Pescara, Chieti Scalo, Italy; ^g^ Haupt Pharma Latina srl Aenova Group, Rome, Italy; ^h^ Algo Biotechnologies srl, Chieti Scalo, Italy

**Keywords:** Antibacterial, biofilm, menthol, molecular modelling, wound healing

## Abstract

In this work, 17 derivatives were synthesised by combining halogenated and non-halogenated cinnamoyl scaffolds with menthol and tested against a panel of Gram-positive and Gram-negative bacteria. Among the synthesised derivatives, **MF1** and **MCl2** demonstrated enhanced therapeutic potential. **MF1** showed the most potent antimicrobial activity (MIC values ranging from 8 to 64 mg/L against *E. faecium*), representing a significant improvement over menthol, with a five-fold reduction in MIC_50_. Additionally, **MF1** effectively reduced biofilm biomass production by 50% in *S. aureus* and by 20% in *P. aeruginosa* at sub-MIC concentrations. **MCl2** reduced biomass by up to 40% in *A. baumannii* at the lowest subMIC concentrations tested (0.06 x MIC). Moreover, **MCl2** showed potential as a wound healing agent promoting fibroblast-mediated repair within just 24 h. Notably, both compounds exhibited no cytotoxic effects. Molecular docking and molecular dynamics simulations confirmed strong binding affinity and high stability of **MF1** and **MCl2** with the target protein.

## Graphical Abstract

**Figure:**
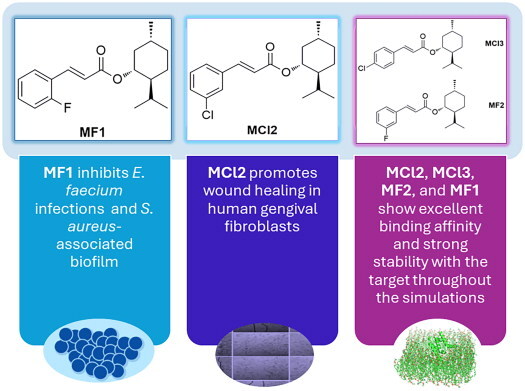

## Introduction

Natural products have long been recognised as important sources of lead compounds and inspiration for the development of novel drugs and agrochemicals^1^^,^^2^. Menthol, a cyclic monoterpene alcohol, is a prominent component of the essential oils extracted from *Mentha canadensis L.* (cornmint) and *M. piperita L.* (peppermint)^3^. Serving as the principal constituent in mint essential oil, L-menthol is responsible for the distinctive taste, fragrance, and cooling sensation associated with mint. Its versatile properties include antibacterial, antifungal, analgesic, anaesthetic, chemopreventive, and immunomodulatory effects^4–6^.

Notably, peppermint oil has been traditionally used to alleviate symptoms of stomachache and indigestion and has been studied for its potential benefits in managing symptoms of irritable bowel syndrome (IBS). It is believed to exert a relaxing effect on gastrointestinal muscles, which may help alleviate abdominal pain and bloating^7^. Moreover, it exhibits antimicrobial activity against some Gram-positive bacteria, including *Staphylococcus aureus* and *Enterococcus faecalis*^8^. This antimicrobial effect is primarily attributed to its ability to disrupt the integrity of microbial cell membranes, thereby affecting their structure and function. However, the clinical use of menthol is hindered by its rapid metabolism in the liver. Specifically, the hydroxyl group of menthol undergoes glucuronidation by UDP-glucuronosyltransferase 2B7, leading to primarily local pharmacological effects and limited systemic bioavailability. Studies have shown that after oral administration, only 1% of menthol is detectable in human plasma. Additionally, high volatility, chemical instability, and poor water solubility further restrict its application and bioavailability. To overcome these pharmacokinetic limitations, the primary aim of this study was to develop novel menthol-based derivatives by chemically masking the hydroxyl group with cinnamoyl moieties^9^.

The incorporation of cinnamoyl scaffolds serves a dual purpose in enhancing the properties of menthol. Primarily, it provides a protective effect by masking the hydroxyl group, which is susceptible to the hepatic first-pass effect. This structural modification not only stabilises the reactive hydroxyl moiety but also introduces a moiety that may confer additional pharmacological benefits. The cinnamoyl scaffold could contribute to an enhancement in the antimicrobial activity of menthol, thereby synergistically improving its overall efficacy. As reported in the literature, cinnamic acid is a naturally occurring compound with recognised antibacterial properties^10^. Notably, at sub-lethal concentrations (250 µg/mL), it reduces the production of virulence factors and biofilm formation in *Pseudomonas aeruginosa* PAO1^11^. It also inhibits the *Escherichia coli* gut microbial β-glucuronidase, whose overexpression is associated with gastrointestinal toxicity (IC_50_ = 3.2–22.2 mM)^12^. Several acid cinnamic derivatives have demonstrated activity against significant pathogens as *Mycobacterium tuberculosis*, *S. aureus*, and *Streptococcus pyogenes* 10535^13^.

Another objective of this study was to explore the structural activity-relationship of the menthol-based antimicrobials by introducing various functionalities on the phenyl ring of the cinnamic acid moiety. The cinnamic acid derivatives used herein differ by the presence of a methoxy group or halogen atoms (F, Cl, and Br) in various positions on the phenyl ring. Literature reports suggest that such substitutions influence antimicrobial activity by lowering the minimum inhibitory concentration (MIC) of cinnamic acid. For instance, 4-chlorocinnamic acid inhibits the growth of *S. aureus* ATCC 25925 with a MIC value of 5.09 µmol/mL, while 4-methoxycinnamic acid has demonstrated strong activity against *M. tuberculosis* with a MIC value of 86 µM^14^^,^^15^. Trans-cinnamaldehyde (CNMA) derivatives bearing 2,4-dichloro or fluoro substituents have shown inhibitory activity against *S. pyogenes* and *S. aureus* at concentrations ranging from 16 to 64 µg/mL. Halogenated CNMAs also affect bacterial virulence factors; specifically, 4-bromo-, 4-chloro-, 4-fluoro-, and dichloro-CNMAs have been shown to reduce protease activity, inhibit biofilm formation, and decrease cell surface hydrophobicity in both *Candida albicans* and *Vibrio spp*^16^.

In this study, chemical modifications and structural variations of cinnamic acids were investigated to assess the mechanisms of action and the structure-activity relationships of the novel menthol-based antimicrobials to antibacterial activity. Seventeen menthol-based antimicrobials were synthesised and evaluated against a representative panel of Gram-positive (*S. aureus*, *S. epidermidis*, *E. faecalis*, and *E. faecium*) and Gram-negative (*E. coli*, *K. pneumoniae*, *P. aeruginosa*, *A. baumannii*, and *Enterobacter spp*.) bacterial strains. The most active derivatives were further tested for cytotoxicity, biofilm inhibition, and wound healing potential.

Chemoinformatics has become a central tool in drug discovery, enabling the prediction and analysis of molecular interactions through in silico techniques. Among these, molecular docking and molecular dynamics simulations are particularly valuable for evaluating the binding affinity and stability of ligand–target complexes under simulated physiological conditions. Recent studies have successfully applied these methods to investigate novel bioactive compounds, such as thiazolidine-2,4-dione derivatives, functionalised isoxazoles, and various sugar-based analogs, demonstrating their antimicrobial, anticancer, and pharmacokinetic potentials^17^. Thus, in our study, computational studies were conducted to gain further insights into the interactions between the menthol-based antimicrobials and selected bacterial targets^18–21^.

## Experimental

### Materials and methods

All reagents were purchased from Sigma-Aldrich Co. (St. Louis, MO, USA). Chromatographic separations were carried out using column chromatography on silica gel (Merck 60, 230–400 mesh ASTM silica gel). The chemical structures of all synthesised compounds were verified by ^1^H- and ^13^C-NMR spectroscopy, using a Varian VXR-300 spectrometer (Varian Medical Systems, Inc., Palo Alto, CA, USA). The purity of the final compounds was assessed via HPLC. The HPLC system used was an Agilent 1260 Infinity II HPLC (Agilent, Santa Clara, CA, USA), equipped with a 1260 Infinity II Quaternary Pump (model G7111A), 1260 Infinity II Autosampler (model G7129A), 1260 Infinity II Multicolumn Thermostat (model G7116A), and a 1260 Infinity II Diode Array Detector (model G7115A). Data acquisition and integration were performed using Agilent OpenLAB CDS LC ChemStation software. Separation was achieved using a Poroshell 120 EC-C18 column (150 × 4.6 mm, particle size 4 µm, Agilent, Santa Clara, USA) at a temperature of 20 °C. The compounds were dissolved in acetonitrile (125 μg/mL), and the samples were run with a mobile phase consisting of water (A) and acetonitrile (B), both containing 0.1% v/v trifluoroacetic acid. The flow rate was set to 0.8 ml/min, and the UV detection wavelength was 254 nm. All compounds showed > 95% purity by HPLC analysis. The exact masses of the final compounds were determined using a high-resolution mass spectrometer. **MC1-7**, **MCl1-4**, **MBr1-2**, and **MF1-4** were dissolved in an ACN/H_2_O mixture (80/20) containing 0.1% formic acid at a concentration of 10 µg/mL and introduced into the mass spectrometer via a syringe pump at a flow rate of 5 µL/min. Mass spectrometric analysis was performed using a Thermo Fisher Orbitrap Fusion^™^ Tribrid^™^ mass spectrometer operating in MS scan mode within the m/z range of 80 to 500. The instrument was equipped with an Orbitrap detector set to a resolution of 240,000 (FWHM). All compounds were analysed in positive ion mode.

### General procedure for the synthesis of MC1-7

Cinnamic acid and its derivatives (1 eq) were refluxed in 10 ml of thionyl chloride (SOCl_2_) for 1.5 h. The reaction mixture was then evaporated under pressure, and the residue was dissolved in CH_2_Cl_2_ (5 ml). Menthol (1 eq), dissolved in 5 ml of CH_2_Cl_2_, and triethylamine (TEA) (2 eq) were added at 0 °C, and the reaction mixture was stirred for 24 h at rt. The solvent was evaporated to dryness, and the compound was quenched with 1 M HCl (10 ml), extracted with CH_2_Cl_2_, and dried over MgSO_4_. The crude residue was concentrated under reduced pressure and purified by column chromatography on silica gel using CH_2_Cl_2_ as eluent, yielding **MC1-7** as oils.

#### (1R,2S,5R)-2-isopropyl-5-methylcyclohexyl cinnamate (MC1)

Yield: 35%; Rf = 0.80, CH_2_Cl_2_; ^1^H NMR (300 MHz, CDCl_3_) δ: 0.78 (3H, d, *J* = 6.9 Hz), 0.96 (7H, b), 1.06–1.10 (3H, *m*), 1.41–1.52 (1H, *m*), 1.67–1.72 (2H, d, *J* = 14.1 Hz), 1.91 (1H, *m*), 2.04 (1H, d, *J* = 9.9 Hz), 4.81 (1H, *m*), 6.44 (1H, d, *J* = 20.4 Hz), 7.37 (2H, *s*), 7.53 (2H, *s*), 7.68 (1H, d, *J* = 20.4 Hz); ^13^C NMR (75 MHz, CDCl_3_) δ: 16.42 (CH_3_), 20.80 (CH_3_), 22.08 (CH_3_), 23.48 (CH_2_), 26.30 (CH), 31.42 (CH), 34.28 (CH_2_), 41.01 (CH_2_), 47.18 (CH), 74.24 (CH), 118.70 (CH), 128.05 (CH), 128.86 (2 x CH), 130.15 (2 x CH), 134.50 (C), 144.39 (CH), 166.62 (CO). Calcd for C_19_H_26_O_2_: C, 79.68; H, 9.15; O, 11.17. Found: C, 79.70; H, 9.12; O, 11.18. HR-MS (ESI) m/z: Calcd for C_19_H_26_O_2_, 286.193; found: [M + H]^+^ = 287.1996.

#### (E)-(1R,2S,5R)-2-isopropyl-5-methylcyclohexyl 3-(benzo[d][1,3] dioxol-5-yl)acrylate (MC2)

Yield: 23%; Rf = 0.62, CH_2_Cl_2_; ^1^H NMR (300 MHz, CDCl_3_) δ: 0.78 (3H, d, *J* = 6.9 Hz), 0.91 (6H, d, *J* = 6.9 Hz), 1.01–1.24 (2H, *m*), 1.38–1.55 (2H, *m*), 1.57 (3H, *m*), 1.67–1.72 (2H, *m*), 1.85–1.92 (1H, *m*), 2.02–2.07 (1H, *m*), 4.8 (1H, *m*), 5.99 (2H, *s*), 6.27 (1H, d, *J* = 15.9 Hz), 6.80 (1H, d, *J* = 7.8 Hz), 6.98 (1H, d, *J* = 7.8 Hz), 7.02 (1H, *s*), 7.6 (1H, d, *J* = 15.9 Hz); ^13^C NMR (75 MHz, CDCl_3_) δ: 16.42 (CH_3_), 20.80 (CH_3_), 22.06 (CH_3_), 23.47 (CH_2_), 26.29 (CH), 31.40 (CH), 34.28 (CH_2_), 41.03 (CH_2_), 47.17 (CH), 74.07 (CH), 101.52 (CH), 106.43 (CH), 108.51 (CH), 116.61 (CH), 124.36 (CH), 128.93 (C), 144.07 (CH), 148.28 (C), 149.47 (CH), 166.77 (CO). Calcd for C_20_H_26_O_4_: C, 72.70; H, 7.93; O, 19.37. Found: C, 72.72; H, 7.94; O, 19.34. HR-MS (ESI) m/z: Calcd for C_20_H_26_O_4_, 330.18; found: [M + H]^+^ = 331.1894.

#### (E)-(1R,2S,5R)-2-isopropyl-5-methylcyclohexyl 3–(3,4-dimethoxyphenyl) acrylate (MC3)

Yield: 39%; Rf = 0.48, CH_2_Cl_2_; ^1^H NMR (300 MHz, CDCl_3_) δ: 0.79 (3H, d, *J* = 6.9 Hz), 0.90–0.91 (6H, d, *J* = 6.9 Hz), 1.08–1.24 (1H, *m*), 1.40–1.53 (1H, *m*), 1.57 (2H, *s*), 1.58–1.72 (2H, *m*), 1.90–1.94 (1H, *m*), 2.03–2.07 (1H, *m*), 3.90 (6H, *s*), 4.82 (1H, *m*), 6.32 (1H, d, *J* = 16.2 Hz), 6.84 (1H, d, *J* = 8.1 Hz), 7–07 (1H, *s*), 7.11 (1H, d, *J* = 8.1 Hz), 7.77 (1H, d, *J* = 15.9 Hz); ^13^C NMR (75 MHz, CDCl_3_) δ: 16.31 (CH_3_), 20.76 (CH_3_), 22.03 (CH_3_), 23.38 (CH_2_), 26.20 (CH), 31.34 (CH), 34.22 (CH_2_), 41.01 (CH_2_), 47.15 (CH), 55.81 (2 x CH_3_), 73.89 (CH), 109.25 (CH), 110.81 (CH), 116.24 (CH), 122.57 (CH), 127.38 (C), 144.25 (CH), 149.02 (C), 150.87 (C), 166.69 (CO). Calcd for C_21_H_30_O_4_: C, 72.80; H, 8.73; O, 18.47. Found: C, 72.83; H, 8.74; O, 18.43. HR-MS (ESI) m/z: Calcd for C_21_H_30_O_4_, 346.214; found: [M + H]^+^ = 347.2206.

#### (E)-(1R,2S,5R)-2-isopropyl-5-methylcyclohexyl 3–(4-methoxyphenyl) acrylate (MC4)

Yield: 31%; Rf = 0.74, CH_2_Cl_2_; ^1^H NMR (300 MHz, CDCl_3_) 0.79 (3H, d, *J* = 6.3 Hz), 0.95 (6H, *J* = 17.1 Hz), 0.97–1.09 (2H, *m*), 1.40–1.52 (1H, *m*), 1.67–1.72 (2H, d, *J* = 20.1 Hz), 1.92 (1H, *m*), 2.04 (1H, d, *J* = 11.7 Hz), 3.83 (3H, *s*), 4.84 (1H, *m*), 6.32 (1H, d, *J* = 15.6 Hz), 6.92 (2H, d, *J* = 8.7 Hz), 7.46 (2H, d, *J* = 8.7 Hz), 7.59 (1H, d, *J* = 16.2 Hz).^13^C NMR (75 MHz, CDCl_3_) δ: 16.40 (CH_3_), 20.81 (CH_3_), 22.09 (CH_3_), 23.47 (CH_2_), 26.29 (CH), 31.42 (CH), 34.30 (CH_2_), 41.04 (CH_2_), 47.21 (CH), 55.37 (CH_3_), 73.99 (CH), 114.26 (2 x CH), 116.15 (CH), 127.22 (C), 129.68 (2 x CH), 144.05 (CH), 160.00 (C), 166.97 (CO); Calcd for C_20_H_28_O_3_: C, 75.91; H, 8.92; O, 15.17. Found: C, 75.93; H, 8.94; O, 15.13. HR-MS (ESI) *m*/*z*: Calcd for C_20_H_28_O_3_, 316.204; found: [M + H]^+^ = 317.2102.

#### (E)-(1R,2S,5R)-2-isopropyl-5-methylcyclohexyl 3–(2-methoxyphenyl) acrylate (MC5)

Yield: 27%; Rf = 0.90, CH_2_Cl_2_; ^1^H NMR (300 MHz, CDCl_3_) δ: 0.80 (3H, d, *J* = 6.9 Hz), 0.90 (6H, d, *J* = 11.1 Hz), 0.92–1.12 (2H, *m*), 1.41–1.52 (1H, *m*), 1.66–1.73 (2H, *m*), 1.95 (1H, *m*), 2.04 (1H, *m*), 3.88 (3H, *s*), 4.83 (1H, *m*), 6.55 (1H, d, *J* = 16.5 Hz), 6.89 (1H, d, *J* = 8.7 Hz), 6.95 (1H, d, *J* = 7.8 Hz), 7.32 (1H, *t*), 7.49 (1H, d, *J* = 7.8 Hz), 7.95 (1H, d, *J* = 16.5 Hz); ^13^C NMR (75 MHz, CDCl_3_) δ: 16.43 (CH_3_), 20.78 (CH_3_), 22.10 (CH_3_), 23.5 (CH_2_), 26.27 (CH), 31.4 (CH), 34.30 (CH_2_), 41.04 (CH_2_), 47.16 (CH), 55.35 (CH_3_), 73.89 (CH), 111.02 (CH), 119.12 (CH), 120.64 (CH), 123.38 (CH), 128.89 (C), 131.37 (CH), 139.83 (CH), 158.24 (C), 167.03 (CO). Calcd for C_20_H_28_O_3_: C, 75.91; H, 8.92; O, 15.17. Found: C, 75.93; H, 8.93; O, 15.14. HR-MS (ESI) *m*/*z*: Calcd for C_20_H_28_O_3_, 316.204; found: [M + H]^+^ = 317.3103.

#### (E)-(1R,2S,5R)-2-isopropyl-5-methylcyclohexyl 3–(3-methoxyphenyl) acrylate (MC6)

Yield: 30%; Rf = 0.74, CH_2_Cl_2_; ^1^H NMR (300 MHz, CDCl_3_) δ: 0.80 (2H, d, *J* = 6.3 Hz), 0.90 (6H, d, *J* = 6.9 Hz), 0.98–1.11 (1H, *m*), 1.41–1.52 (2H, *m*), 1.56 (1H, *s*), 1.68 (2H, *m*), 1.91–1.93 (1H, *m*), 2.03–2.08 (1H, *m*), 3.83 (3H, *s*), 4.81 (1H, *m*), 6.44 (1H, d, *J* = 15.6 Hz), 6.93 (1H, d, *J* = 10.5 Hz), 7.04 (1H, s), 7.13 (1H, d, *J* = 6.9 Hz), 7.29 (1H, d, *J* = 8.1 Hz), 7.65 (1H, d, *J* = 15.6 Hz); ^13^C NMR (75 MHz, CDCl_3_) δ: 16.39 (CH_3_), 20.78 (CH_3_), 22.06 (CH_3_), 23.48 (CH_2_), 26.30 (CH), 31.41 (CH), 34.27 (CH_2_), 40.99 (CH_2_), 47.17 (CH), 55.26 (CH3), 74.25 (CH), 112.73 (CH), 116.10 (CH), 118.97 (CH), 120.77 (CH), 129.83 (CH), 135.86 (C), 144.30 (CH), 159.84 (C), 166.52 (CO). Calcd for C_20_H_28_O_3_: C, 75.91; H, 8.92; O, 15.17. Found: C, 75.93; H, 8.91; O, 15.16. HR-MS (ESI) *m*/*z*: Calcd for C_20_H_28_O_3_, 316.204; found: [M + H]^+^ = 317.2103.

#### (E)-(1R,2S,5R)-2-isopropyl-5-methylcyclohexyl 3–(3,4,5-trimethoxyphenyl) acrylate (MC7)

Yield: 10% Rf = 0.45, CH_2_Cl_2_; ^1^H NMR (300 MHz, CDCl_3_) δ: 0.79 (3H, d, *J* = 6.3 Hz), 0.90 (7H, d, *J* = 8.7 Hz), 0.92–1.06 (2H, *m*), 1.44–1.49 (2H, *m*), 1.67 (2H, d, *J* = 12.9 Hz), 1.93 (1H, *m*), 2.04 (1H, d, *J* = 11.7 Hz), 3.87 (9H, *s*), 4.82 (1H, *m*), 6.35 (1H, d, *J* = 15.9 Hz), 6.74 (2H, *s*), 7.60 (1H, d, *J* = 15.9 Hz); ^13^C NMR (75 MHz, CDCl_3_) δ: 20.78 (CH_3_), 22.03 (2 x CH_3_), 23.44 (CH_2_), 26.26 (CH), 31.36 (CH), 34.25 (CH_2_), 41.03 (CH_2_), 47.21 (CH), 56.07 (2 x CH_3_), 60.90 (CH_3_), 74.18 (CH), 105.06 (2 x CH), 117.92 (CH), 130.02 (CH), 139.87 (C), 144.33 (CH), 153.36 (2 x C), 166.48 (CO). Calcd for C_22_H_32_O_5_: C, 75.91; H, 8.92; O, 15.17. Found: C, 75.93; H, 8.89; O, 15.18. HR-MS (ESI) *m*/*z*: Calcd for C_22_H_32_O_5_, 376.225; found: [M + H]^+^ = 377.2310.

### General procedure for the synthesis of MF1-4, MCl1-4, MBr1-2

To a solution of cinnamic acid derivatives (1 eq) in dry DCM (10 ml), menthol (1 eq), diclyclohexylcarbodiimide (DCC, 1 eq), and 4-(dimethylamino) pyridine (DMAP, 0.10 eq) were added. The mixture was stirred overnight at 0 °C and the solvent was removed under vacuum. The crude residue was then dissolved in CH_2_Cl_2_ and washed with 10% citric acid, 10% NaHCO_3_, water, and brine, and dried over anhydrous sodium sulphate. The compounds were filtered and purified by column chromatography on silica gel using CH_2_Cl_2_ as the eluent. These compounds were obtained as oils.

#### (E)-(1R,2S,5R)-2-isopropyl-5-methylcycloheyl 3–(2-fluorophenyl) acrylate (MF1)

Yield: 64%; Rf = 0.81, CH_2_Cl_2_; ^1^H NMR (300 MHz, CDCl_3_) δ: 0.77 (3H, d, *J* = 7.2 Hz), 0.89 (6H, d, *J* = 6.9 Hz), 1.03 (3H, *m*), 1.42 (2H, *m*), 1.67 (2H, d, *J* = 11.1 Hz), 1.89 (1H, *m*), 2.03 (1H, d, *J* = 6.6 Hz), 4.81 (1H, *m*), 6.52 (1H, br d), 7.10 (2H, *m*), 7.26 (1H, *m*), 7.48 (1H, *t*), 7.74 (1H, d, *J* = 16.5 Hz); ^13^C NMR (75 MHz, CDCl_3_) δ: 16.40 (CH_3_), 20.74 (CH_3_), 22.03 (CH_3_), 23.48(CH_2_), 26.31 (CH), 31.40 (CH), 34.25 (CH_2_), 40.95 (CH_2_), 47.11 (CH), 74.35 (CH), 115.98, 121.27, 124.36, 129.01, 131.47, 136.94, 159.60, 162.95, 166.36. Calcd for C_19_H_25_FO_2_: C, 74.97; H, 8.28; F, 6.24; O, 10.51. Found: C, 74.99; H, 8.29; F, 6.22; O, 10.50.

#### (E)-(1R,2S,5R)-2-isopropyl-5-methylcyclohexyl 3–(3-fluorophenyl) acrylate (MF2)

Yield: 81%; Rf = 0.87, CH_2_Cl_2_; ^1^H NMR (300 MHz, CDCl_3_) δ: 0.79 (3H, d, *J* = 6.9 Hz), 0.88 (7H), 1.02 (2H, *m*), 1.48 (2H, *m*), 1.72 (2H, *m*), 1.90 (1H, *m*), 2.07 (1H, d, *J* = 11.1 Hz), 4.85 (1H, *m*), 6.45 (1H, d, *J* = 16.2 Hz), 7.09 (1H, *m*), 7.33–7.38 (3H, *m*), 7.57 (1H, d, *J* = 16.5 Hz); ^13^C NMR (75 MHz, CDCl_3_) δ: 16.40 (CH_3_), 20.75 (CH_3_), 22.03 (CH_3_), 23.50 (CH_2_), 26.32 (CH), 31.42 (CH), 34.25 (CH_2_), 40.97 (CH_2_), 47.16 (CH), 74.48 (CH), 114.10, 117.13, 120.13, 124.04, 130.32, 136.73, 142.97, 164.62 (C), 166.20 (C). Calcd for C_19_H_25_FO_2_: C, 74.97; H, 8.28; F, 6.24; O, 10.51. Found: C, 74.95; H, 8.23; F, 6.28; O, 10.54.

#### (E)-(1R,2S,5R)-2-isopropyl-5-methylcyclohexyl 3–(4-fluorophenyl) acrylate (MF3)

Yield: 81%; Rf = 0.79, CH_2_Cl_2_; ^1^H NMR (300 MHz, CDCl_3_) δ: 0.73 (3H, d, *J* = 6.6 Hz), 0.84 (6H, d, *J* = 6.6 Hz), 0.98 (3H, *m*), 1.41 (2H, *m*), 1.63 (2H, d, *J* = 11.1 Hz), 1.88 (1H, *m*), 2.00 (1H, d, *J* = 11.7 Hz), 4.77 (1H, *m*), 6.31 (1H, d, *J* = 15.9 Hz), 6.97 (2H, *t*), 7.42 (2H, *m*), 7.54 (1H, d, *J* = 15.9 Hz); ^13^C NMR (75 MHz, CDCl_3_) δ: 16.38 (CH_3_), 20.72 (CH_3_), 22.00 (CH_3_), 23.47 (CH_2_), 26.31 (CH), 31.37 (CH), 34.25 (CH_2_), 40.98 (CH_2_), 47.16 (CH), 74.19 (CH), 115.78 (CH), 116.07 (CH), 118.42 (CH), 129.89 (CH), 130.76 (CH), 142.98 (CH), 162.08 (C), 165.43 (C), 166.34 (CO). Calcd for C_19_H_25_FO_2_: C, 74.97; H, 8.28; F, 6.24; O, 10.51. Found: C, 74.96; H, 8.30; F, 6.27; O, 10.47.

#### (E)-(1R,2S,5R)-2-isopropyl-5-methylcyclohexyl 3–(3,4-difluorophenyl) acrylate (MF4)

Yield: 67%; Rf = 0.80, CH_2_Cl_2_; ^1^H NMR (300 MHz, CDCl_3_) δ: 0.79 (3H, d, *J* = 6.9 Hz), 0.92 (7H, m), 1.00–1.11 (2H, *m*), 1.40–1.56 (2H, *m*), 1.68 (2H, d, *J* = 13.5 Hz), 1.90 (1H, *m*), 2.02 (1H, d, *J* = 12.3 Hz), 4.81 (1H, *m*), 6.36 (1H, d, *J* = 16.2 Hz), 7.14 (1H, *m*), 7.25 (1H, *m*), 7.34 (1H, *t*), 7.57 (1H, d, *J* = 15.6 Hz); ^13^C NMR (75 MHz, CDCl_3_) δ: 16.39 (CH_3_), 20.75 (CH_3_), 22.02 (CH_3_), 23.48 (CH_2_), 26.34 (CH), 31.40 (CH), 34.24 (CH_2_), 40.97 (CH_2_), 47.16 (CH), 74.55 (CH), 116.13 (CH), 116.36 (CH), 117.64 (CH), 117.89 (CH), 119.87 (C), 124.63 (C), 124.77 (C), 141.96 (CH), 166.05 (CO). Calcd for C_19_H_24_F_2_O_2_: C, 70.78; H, 7.50; F, 11.79; O, 9.93. Found: C, 70.80; H, 7.53; F, 11.75; O, 9.92.

#### (E)-(1R,2S,5R)-2-isopropyl-5-methylcycloheyl 3–(2-chlorophenyl) acrylate (MCl1)

Yield: 75%; Rf = 0.86, CH_2_Cl_2_; ^1^H NMR (300 MHz, CDCl_3_) δ: 0.87 (3H, d, *J* = 7.2 Hz), 0.94 (7H, d, *J* = 6.3 Hz), 0.98 (2H, *m*), 1.67 (2H, *m*), 1.70 (2H, d, *J* = 12.9 Hz), 1.91 (1H, *m*), 2.07 (1H, d, *J* = 12.3 Hz), 4.85 (1H, *m*), 6.39 (1H, d, *J* = 16.2 Hz), 7.25 (2H, *m*), 7.40 (1H, d, *J* = 9.3 Hz), 7.60 (1H, d, *J* = 6.9 Hz), 8.04 (1H, d, *J* = 16.5 Hz); ^13^C NMR (75 MHz, CDCl_3_) δ: 16.53 (CH_3_), 20.74 (CH_3_), 22.05 (CH_3_), 23.59 (CH_2_), 26.40 (CH), 31.40 (CH), 34.25 (CH_2_), 40.95 (CH_2_), 47.13 (CH), 74.47 (CH), 121.31(CH), 127.03 (CH), 127.58 (CH), 130.12 (CH), 130.90 (CH), 132.76 (CH), 134.88 (C), 140.12 (CH), 166.05 (CO). Calcd for C_19_H_25_ClO_2_: C, 71.12; H, 7.85; Cl, 11.05; O, 9.97. Found: C, 71.14; H, 7.88; Cl, 11.02; O, 9.95. HR-MS (ESI) m/z: Calcd for C_19_H_25_ClO_2_, 320.154; found [M + H]^+^ = 321.1609.

#### (E)-(1R,2S,5R)-2-isopropyl-5-methylcyclohexyl 3–(3-chlorophenyl) acrylate (MCl2)

Yield: 78%; Rf = 0.88, CH_2_Cl_2_; ^1^H NMR (300 MHz, CDCl_3_) δ: 0.79 (3H, d, *J* = 6.9 Hz), 0.91 (7H, d, *J* = 9.3 Hz), 1.09 (2H, *m*), 1.47 (2H, *m*), 1.70 (2H, d, *J* = 10.5 Hz), 1.91 (1H, *m*), 2.07 (1H, d, *J* = 11.7 Hz), 4.80 (1H, *m*), 6.45 (1H, d, *J* = 16.2 Hz), 7.39 (3H, *m*), 7.50 (1H, *s*), 7.61 (1H, d, *J* = 15.6 Hz); ^13^C NMR (75 MHz, CDCl_3_) δ: 16.40 (CH_3_), 20.78 (CH_3_), 22.05 (CH_3_), 23.47 (CH_2_), 26.31 (CH), 31.40 (CH), 34.24 (CH_2_), 40.95 (CH_2_), 47.15 (CH), 74.45 (CH), 120.18 (CH), 126.20 (CH), 127.74 (CH), 129.99 (CH), 130.08 (CH), 134.85 (C), 136.33 (C), 142.72 (CH), 166.14 (CO). Calcd for C_19_H_25_ClO_2_: C, 71.12; H, 7.85; Cl, 11.05; O, 9.97. Found: C, 71.15; H, 7.88; Cl, 11.02; O, 9.94. HR-MS (ESI) *m*/*z*: Calcd for C_19_H_25_ClO_2_, 320.154; found [M + H]^+^ = 321.1609.

#### (E)-(1R,2S,5R)-2-isopropyl-5-methylcyclohexyl 3–(4-chlorophenyl) acrylate (MCl3)

Yield: 66%; Rf = 0.90, CH_2_Cl_2_; ^1^H NMR (300 MHz, CDCl_3_) δ: 0.77 (3H, d, *J* = 9.6 Hz), 0.90 (7H, d, *J* = 7.2 Hz), 1.03 (2H, *m*), 1.45 (2H, *m*), 1.67 (2H, d, *J* = 12.9 Hz), 1.90 (1H, *m*), 2.05 (1H, d, *J* = 11.7 Hz), 4.80 (1H, *m*), 6.40 (1H, d, *J* = 18.6 Hz), 7.31 (2H, d, *J* = 9.0 Hz), 7.40 (2H, d, *J* = 10.5 Hz), 7.56 (1H, d, *J* = 18.0 Hz); ^13^C NMR (75 MHz, CDCl_3_) δ: 16.40 (CH_3_), 20.75 (CH_3_), 22.03 (CH_3_), 23.45 (CH_2_), 26.30 (CH), 31.39 (CH), 34.25 (CH_2_), 40.98 (CH_2_), 47.15 (CH), 74.32 (CH), 119.29 (CH), 129.08 (2 x CH), 129.16 (2 x CH), 132.99 (C), 135.98 (C), 142.85 (CH), 166.22 (CO). Calcd for C_19_H_25_ClO_2_: C, 71.12; H, 7.85; Cl, 11.05; O, 9.97. Found: C, 71.11; H, 7.83; Cl, 11.09; O, 9.96. HR-MS (ESI) *m*/*z*: Calcd for C_19_H_25_ClO_2_, 320.154; found [M + H]^+^ = 321.1610.

#### (E)-(1R,2S,5R)-2-isopropyl-5-methylcyclohexyl 3–(3,4-dichlorophenyl) acrylate (MCl4)

Yield: 78%; Rf = 0.94, CH_2_Cl_2_; ^1^H NMR (300 MHz, CDCl_3_) δ:0.77 (3H, d, *J* = 6.6 Hz), 0.88 (6H, d, *J* = 6.9 Hz), 1.08 (2H, *m*), 1.46 (2H, *m*), 1.69 (2H, d, *J* = 10.8 Hz), 2.02 (1H, *m*), 2.02 (2H, d, *J* = 9.3 Hz), 4.80 (1H, *m*), 6.42 (1H, d, *J* = 16.5 Hz), 7.32 (1H, d, *J* = 8.7 Hz), 7.40 (1H, d, *J* = 8.4 Hz), 7.50 (1H, s), 7.57 (1H, d, *J* = 6.3 Hz); ^13^C NMR (75 MHz, CDCl_3_) δ: 16.37 (CH_3_), 20.77 (CH_3_), 22.03 (CH_3_), 23.45 (CH_2_), 26.31 (CH), 31.39 (CH), 34.22 (CH_2_), 40.94 (CH_2_), 47.13 (CH), 74.55 (CH), 120.63 (CH), 126.97 (CH), 129.53 (CH), 130.81 (CH), 133.14 (C), 133.97 (C), 134.56 (C), 141.53 (CH), 165.88 (CO). Calcd for C_19_H_24_Cl_2_O_2_: C, 64.23; H, 6.81; Cl, 19.96; O, 9.01. Found: C, 64.21; H, 6.83; Cl, 19.98; O, 8.99. HR-MS (ESI) m/z: Calcd for C_19_H_24_Cl_2_O_2_, 354.115; found [M + H]^+^ = 355.1219.

#### (E)-(1R,2S,5R)-2-isopropyl-5-methylcycloheyl 3–(3-bromophenyl) acrylate (MBr1)

Yield: 63%; Rf = 0.81, CH_2_Cl_2_; ^1^H NMR (300 MHz, CDCl_3_) δ: 0.79 (3H, d, *J* = 6.9 Hz), 0.92 (6H, d, *J* = 14.1 Hz), 1.05–1.11 (1H, *m*), 1.40–1.55 (2H, *m*), 1.57 (1H, *s*), 1.66–1.74 (2H, *m*), 1.88–1.92 (1H, *m*), 2.02–2.08 (1H, *m*), 4.84 (1H, *m*), 6.44 (d, 1H, *J* = 16.2 Hz), 7.39 (1H, *t*), 7.45 (2H, *m*), 7.47 (d, 1H, *J* = 15.9 Hz), 7.68 (*s*, 1H); ^13^C NMR (75 MHz, CDCl_3_) δ: 16.43 (CH_3_), 20.77 (CH_3_), 22.03 (CH_3_), 23.53 (CH_2_), 26.35 (CH), 31.40 (CH), 34.27 (CH_2_), 40.97 (CH_2_), 47.17 (CH), 74.45 (CH), 120.25 (CH), 122.98 (C), 126.60 (CH), 130.32 (CH), 130.67 (CH), 132.37 (CH), 136.64 (C), 142.56 (CH), 166.04 (CO). Calcd for C_19_H_25_BrO_2_: C, 62.47; H, 6.90; Br, 21.87; O, 8.76 Found: C, 62.49; H, 6.92; Br, 21.85; O, 8.74. HR-MS (ESI) *m*/*z*: Calcd for C_19_H_25_BrO_2_, 364.104; found [M + H]^+^ = 365.1102.

#### (E)-(1R,2S,5R)-2-isopropyl-5-methylcyclohexyl 3–(4-bromophenyl) acrylate (MBr2)

Yield: 78%; Rf = 0.73, CH_2_Cl_2_; 1H NMR (300 MHz, CDCl_3_) δ: 0.79 (3H, d, *J* = 6.9 Hz), 0.92 (7H, d, *J* = 9.9 Hz), 1.01–1.11 (2H, *m*), 1.40–1.54 (2H, *m*), 1.59 (1H, *s*), 1.66–1.71 (2H, *m*), 1.87–1.93 (1H, *m*), 2.06 (1H, *m*), 4.84 (1H, *m*), 6.43 (d, 1H, *J* = 15.9 Hz), 7.39 (1H, d, *J* = 9.0 Hz), 7.49 (d, 1H, *J* = 8.4 Hz), 7.61 (d, 1H, *J* = 15.6 Hz); ^13^C NMR (75 MHz, CDCl_3_) δ: 16.42 (CH_3_), 20.77 (CH_3_), 22.05 (CH_3_), 23.51 (CH_2_), 26.34 (CH), 31.40 (CH), 34.27 (CH_2_), 40.98 (CH_2_), 47.18 (CH), 74.38 (CH), 119.44 (CH), 124.34 (C), 129.39 (2 x CH), 132.06 (2 x CH), 133.45 (C), 142.92 (CH), 166.23 (CO). Calcd for C_19_H_25_BrO_2_: C, 62.47; H, 6.90; Br, 21.87; O, 8.76. Found: C, 62.48; H, 6.92; Br, 21.85; O, 8.75. HR-MS (ESI) *m*/*z*: Calcd for C_19_H_25_BrO_2_, 364.104; found [M + H]^+^ = 365.1103.

### In silico evaluation of physicochemical properties

*In silico* assessment of the physicochemical properties of all synthesised antimicrobials was conducted using the SwissADME online tool (accessed online on March 20, 2023, at https://swissadme.ch). To perform the study, the SMILE strings of the compounds were uploaded on SwissADME. This approach aimed to acquire comparative results for various physicochemical parameters, including water solubility, LogP, molecular weight (MW), polar surface area, the count of hydrogen bond donors (HBD) and acceptors (HBA), as well as the number of rotary bonds (Rb), TSPA (Topological Polar Surface Area), and MR (Molecular Refractivity).

### Antimicrobial assays

A total of 80 Gram-positive and Gram-negative clinical strains, including strains from the American Type Culture Collection (ATCC), were chosen to assess the antimicrobial activity of the novel menthol-based compounds. The bacterial strains used in this study are part of our institutional biobank and were routinely collected by the Clinical Microbiology Laboratory of the Regional University Hospital of Ancona (Italy). The collection includes both clinical isolates and reference strains purchased from the American Type Culture Collection (ATCC). All strains are routinely isolated and preserved for microbiological research purposes. No sensitive patient data were accessed, and no patients were specifically recruited for this study. However, informed consent for the use of biological materials for research purposes is routinely obtained from patients upon hospital admission. As the study did not involve any clinical data or patient intervention, ethical approval was not required according to the policies of the Regional University Hospital of Ancona (Italy).

Stock solutions of the derivatives were prepared in DMSO (10 mg/L, w/v) immediately prior to use. The minimal inhibitory concentration (MIC) values were determined using the broth microdilution method, following the guidelines of the European Committee on Antimicrobial Susceptibility Testing (EUCAST) (https://www.eucast.org/ast_of_bacteria/mic_determination). The novel menthol-based antimicrobials were tested at concentrations ranging from 512 to 8 mg/L. Appropriate growth controls were included in all experiments to ensure that DMSO did not affect culture viability. The MIC was defined as the lowest concentration of the compound at which no visible growth of microorganisms was observed.

### Antibiofilm activity and biofilm biomass quantification

The antibiofilm activity of menthol or derivatives was investigated on ATCC and clinical biofilm-producing strains (*S. aureus* ATCC 43300; *S. epidermidis* ATCC 35984; *P. aeruginosa* ATCC 27853; *A. baumannii* ATCC 19606, *S. agalactiae* 343676, *E. faecalis* 304599, and *E. faecium* 254696) by using a microtiter-plate assay as previously described^22^. Briefly, overnight cultures grown in Tryptic Soy Broth (Oxoid, Basingstoke, UK) supplemented with 1% glucose (TSB-G) were harvested by centrifugation and adjusted to an OD_650_ of 0.1 (corresponding to ∼ 1.0 × 10^8^ CFU/mL). 0.1 ml aliquots of inoculum were added to 96-well cell flat-bottom microtiter plates containing 0.1 ml of TSB-G with substances at different sub-Mic concentrations. The microplates were incubated at 37 °C for 24 h under static conditions. Then, the plates were washed three times in phosphate-buffered saline (PBS) to remove non-attached cells, dried for 1 h at 60 °C, and stained with Hucker’s crystal violet. Biomass quantification was assessed by measuring absorbance at 690 nm with a Multiscan Ascent apparatus (Thermo Scientific, Waltham, MA, USA). All tests were performed in triplicate. The results are shown as mean ± standard deviations (SD) of two independent experiments.

### Cell cultures

Human gingival fibroblasts (HGF 24, Code BS CL 138) were obtained from the Experimental Zooprophylactic Institute of Lombardy and Emilia (IZSLER, Brescia, Italy, website: http://www.ibvr.org/Home.aspx) and cultured in Eagle’s Minimum Essential Medium (MEM, HIMEDIA Laboratories, Mumbai, India) supplemented with 10% Foetal bovine serum (FBS, MICROGEM s.r.l., Naples, Italy), along with penicillin (100 U/mL), streptomycin (100 μg/mL), and 25 μg/mL amphotericin B as an antifungal agent (HIMEDIA Laboratories, Mumbai, India).

Human oral keratinocytes (PSC-200–014) were sourced from the American Type Culture Collection (ATCC^®^ Manassas, VA, USA) and grown in Dulbecco’s Modified Eagle Medium (DMEM, HIMEDIA Laboratories, Mumbai, India), supplemented with 7% Foetal bovine serum (FBS, MICROGEM s.r.l., Naples, Italy), penicillin (100 U/mL), streptomycin (100 μg/mL), and 2 μg/mL amphotericin B as an antifungal agent (HIMEDIA Laboratories, Mumbai, India).

Both cell types were maintained in a humidified incubator at 37 °C with 5% CO_2_, with medium changes performed twice weekly. The cells were observed under a Leitz inverted phase-contrast microscope until they reached subconfluence or confluence. For passaging, the cells were treated with trypsin (0.25% trypsin and 0.02% EDTA), counted using a Countess Automated Cell Counter (Thermo Fisher Scientific, Waltham, MA, USA), and plated in specific culture plates as described below. All experiments were conducted between the seventh and ninth subcultures^23^.

#### MTT assay

Cell viability was evaluated using the MTT assay (3-[4,5-dimethylthiazol-2-yl]-2,5-diphenyltetrazolium bromide (Sigma Chemical Co., St. Louis, MO, USA). The MTT assay was used to assess the effects of **MF1**, **MF2**, **MC12**, and **MC13** on the proliferation and viability of human fibroblast and keratinocyte cell lines. Cells were seeded at a density of 1 × 10^4^ cells/well in 96-well plates (Euroclone, Pero, MI, Italy) with 200 µL of DMEM and incubated at 37 °C in a humidified atmosphere containing 5% CO_2_. After 24 h, the cells were treated with **MF1**, **MF2**, **MC12**, or **MC13** at two different concentrations (1.0 and 100 µM) for 24, 48, and 72 h. Untreated control cells received either fresh medium alone or fresh medium with DMSO (0.01% final concentration). At the end of each treatment period, the medium was removed, and the cells were rinsed with PBS. Then, 10 µL of MTT reagent (5 mg/mL stock solution) was added to 100 µL of fresh medium per well. Plates were incubated at 37 °C for 4 h. Following incubation, the resulting formazan crystals were dissolved in 100 µL of DMSO, and absorbance was measured at 570 and 620 nm using a microplate spectrophotometer (Biorad model 680 XR, CA, USA). The absorbance values were directly proportional to the number of viable cells. Data are expressed as means ± standard deviations (SDs) from three independent experiments, each conducted in octuplicate.

#### Scratch assay

Human gingival fibroblasts and oral keratinocytes were plated in 6-well plates (Falcon, Corning, NY, USA) and grown to 90% confluence in 2 ml of medium to assess cell migration. Once the cells reached the desired confluence, the medium was removed, and a straight scratch was made across the centre of each well using a sterile P-200 pipette tip, following a previously established protocol^24^. The cells were gently rinsed with PBS to remove debris and then treated with **MF1**, **MF2**, **MC12**, and **MC13**. Images of the wound closure were captured at 0, 24, and 48 h using a phase-contrast microscope (Olympus, Tokyo, Japan), with photographs taken at 200X magnification. At each time point, the wound area was measured using ImageJ software, and the percentage of wound closure was calculated using the following equation:

(Wound area (0)−Wound area (xh))/Wound area (0)×100

### Docking studies

In reversible (non-covalent) docking, ligand–protein interactions are predicted based on non-covalent binding, allowing the ligand to associate and dissociate freely from the target site^25^. It encompasses various forces like hydrogen bonding, van der Waals, hydrophobic, and electrostatic interactions. The technique forecasts the ligand’s optimal binding within the target protein, finding applications in drug discovery and virtual screening.

Before the molecular docking process, the ligands designated for docking were optimised through Avogadro software version 2.0. Subsequently, the protein structure of *E. faecium* was retrieved from the RCSB database (PDB ID: 7X5N). The crystal complex of *E. faecium* included water molecules and the co-crystallized ligand 5M5 bound to the protein 7X5N. To prepare the protein, we removed all water molecules and non-essential elements and added polar hydrogens to the protein structure, a task accomplished using Discovery Studio software from 2021 (D. Systèmes, “Free Download: BIOVIA Discovery Studio Visualizer,” Dassault Systèmes. Accessed: Feb. 05, 2023. [Online]. Available: https://discover.3ds.com/discovery-studio-visualizer-download). Following the preparation of ligands and the protein, molecular docking was conducted employing AD4 and AutoVina to explore the active site of 7X5N, which was determined based on the area encompassing the co-crystallized ligands (5M5)^26^. The three-dimensional grid was established using the AUTOGRID algorithm, which calculated the binding energy between ligands and their receptor. The default grid size for 7X5N was set to x = 84.61, y = 82.21, and z = 42.54, with a spacing of 0.375 Å between grid points. The docking results obtained from AD4, and Vina were subsequently visualised using Discovery Studio software from 2021.

### Molecular dynamic’s (MD)

MD simulations were conducted using GROMACS version 2023 (M. Abraham *et al*., “GROMACS 2023.2 Manual,” Jul. 2023, doi: 10.5281/zenodo.8134388). The atomic positions within the system were optimised through 250,000 steps of steepest descent energy minimisation. Subsequently, the system was further equilibrated through position-restrained MD simulations, employing a harmonic force constant of 1000 kJ/mol·nm^2^ on all headgroup atoms of the lipid bilayer. Three additional equilibration runs were performed, gradually reducing the force constant to zero to achieve thermodynamic equilibrium. Following the equilibration runs, production MD simulations were carried out.

Following energy minimisation, **MF1**, **MF2**, **MC12**, and **MC13** were prepared for molecular docking using the AutoDock4 software. The resulting complex structures, obtained in conjunction with protein ID 7X5N, were embedded into a membrane environment using the CHARMM-GUI web server. The membrane was configured as a bilayer, with its initial orientation adjusted to align the first principal axis with the Z-axis. The membrane bilayer/ligand system was constructed using the CHARMM-GUI web server^27^. In this setup, a 3:1 ratio of zwitterionic 1,2-dimyristoyl-sn-glycero-3-phosphocholine (DMPC) lipids and anionic 1,2-dimyristoyl-sn-glycero-3-phosphoglycerol (DMPG) lipids was used. It is important to note that, while Gram-positive bacteria typically have a low proportion of zwitterionic lipids—mainly phosphatidylethanolamine—and a high proportion of anionic lipids such as phosphatidylglycerol and cardiolipins in their membranes, our simulation deviated slightly from this composition. In our study, we used DMPC as the zwitterionic lipid, employing a slightly higher DMPC to DMPG ratio (3:1 DMPC/DMPG), in line with common practices in molecular dynamics (MD) simulations. For equilibrium MD simulations, a lipid composition of 96 DMPC and 32 DMPG was generated for each leaflet. A bilayer configuration comprising 48 DMPC and 16 DMPG lipids in each leaflet was employed in MD simulations involving biasing forces. Throughout the equilibrium MD simulations, the compounds were initially positioned outside the lipid bilayer, with an adequate number of water molecules introduced to maintain a hydration level of 70 per lipid. Na^+^ (138) and Cl- ions (31) were added to the system to maintain overall charge neutrality. Simulation Configuration: Lipids were modelled using the CHARMM36 force field in conjunction with the TIP3P water model. **MF1**, **MF2**, **MC12**, and **MC13**, on the other hand, were modelled using the CGENFF36 force field^28^. The parameters for CGENFF36 were automatically generated using the ParamChem service integrated into the CHARMM-GUI web server.

## Results

### Chemistry

**Scheme 1.**
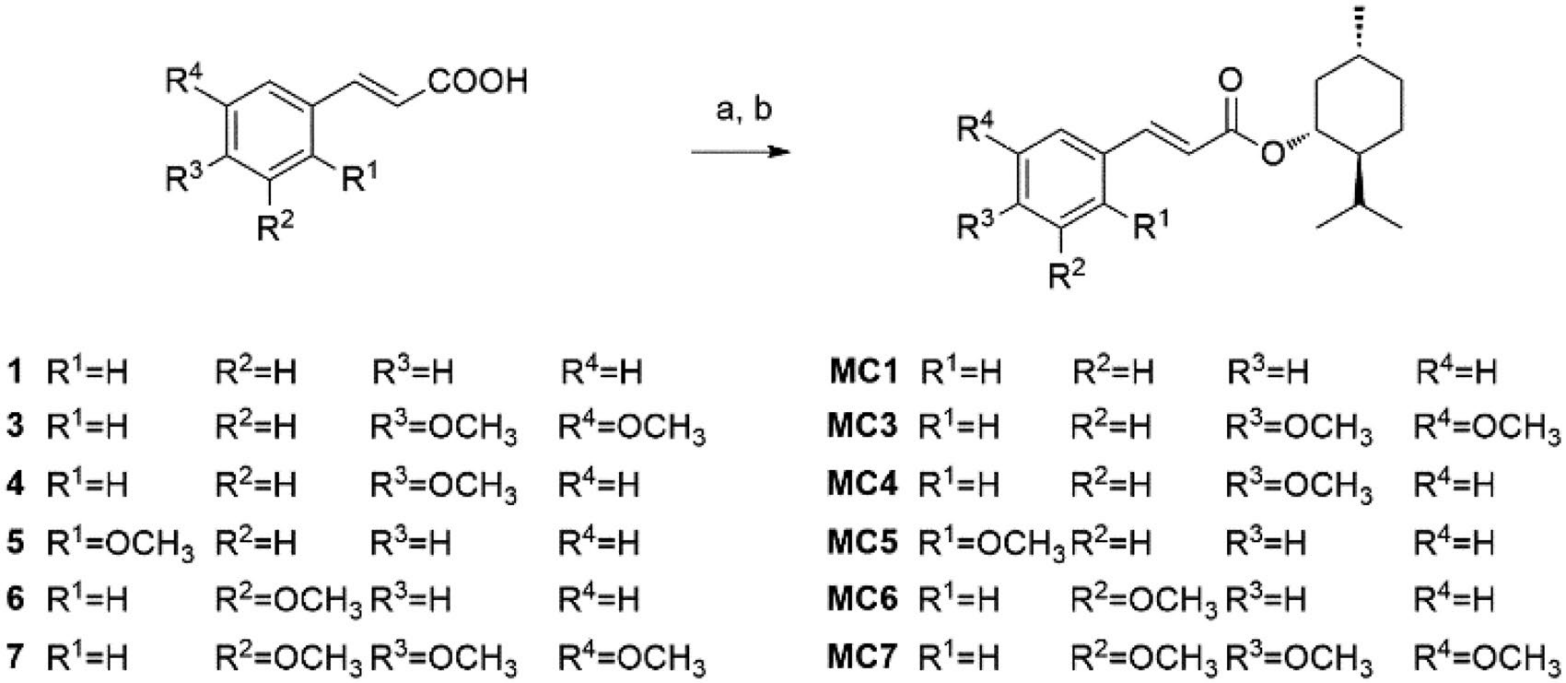
(a) SOCl_2_, DMF, reflux for 4 h; (b) menthol, dry DCM, TEA, at 0 °C and then 12 h at rt.

**MC1** and **MC3-7** were synthetised using the corresponding cinnamoyl derivatives through a two-step process (Scheme 1)^21^. The initial step involved chlorination with SOCl_2_ to facilitate esterification. Notably, the use of SOCl_2_ for activation proved instrumental in achieving higher yields. Subsequently, the second step entailed esterifying the acid chloride derivative with the hydroxy group of menthol, utilising triethylamine (TEA), as illustrated in Scheme 1.

**Scheme 2.**
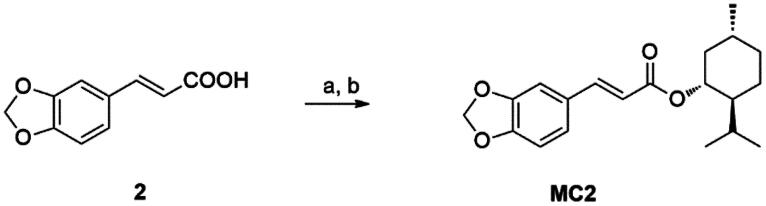
(a) SOCl_2_, DMF, reflux for 4 h; (b) menthol, dry DCM, TEA, at 0 °C and then 12 h at rt.

The synthesis of **MC2**, detailed in Scheme 2, followed a similar procedure, employing benzo[d][1,3]dioxol-5-yl)acrylic acid (**2**) as the initial compound.

**Scheme 3.**
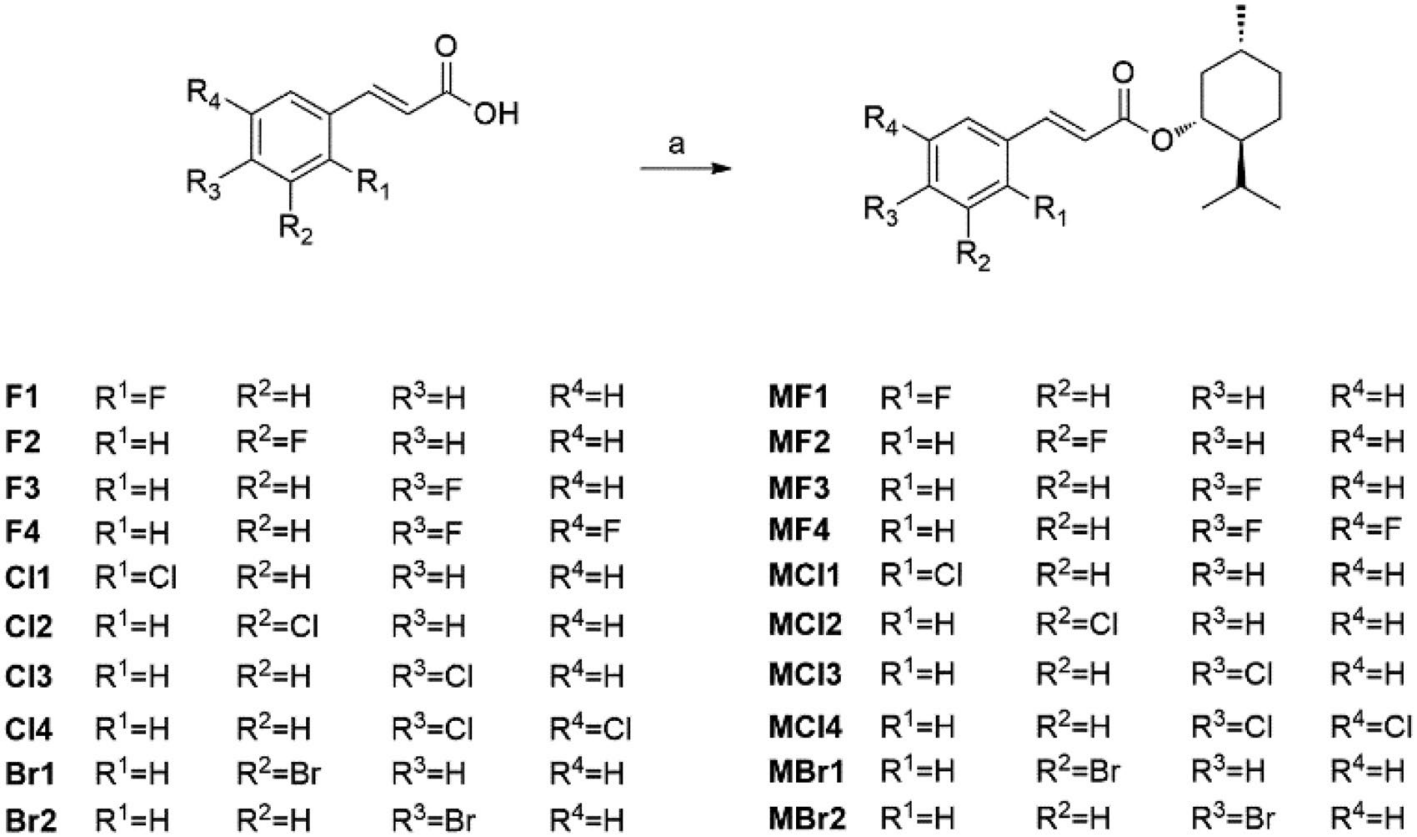
(a) menthol, DCC, DMAP. DCM at 0 °C and then 24 h at rt.

A distinct synthetic approach produced compounds **MF1-4**, **MCl1-4**, and **MBr1–2**. This alternative strategy utilised the respective cinnamoyl derivatives in the presence of *N*, *N*′-dicyclohexylcarbodiimide (DCC), and 4-dimethylaminopyridine (DMAP) for the esterification of the hydroxy group of menthol (Scheme 3).

All newly synthesised menthol-based antimicrobials were characterised by NMR and HR-MS, and their purities were confirmed by HPLC (spectra available in the Supporting information).

### ADME study

ADME studies, which include the analysis of Absorption, Distribution, Metabolism, and Excretion, are fundamental to drug development. In this study, the synthesised compounds underwent comprehensive screening for their pharmacokinetic and physico-chemical properties utilising SwissADME software (Table 1S Supporting Information). Notably, the most promising results emerged for **MC1-7**, revealing adherence to important parameters such as no violation of Lipinski’s rule, a molecular weight (MW) below 500, fewer than 5 hydrogen bond donors (HBD), a logarithm of the partition coefficient (log P) value below 5, and less than 10 hydrogen bond acceptors (HBA). These characteristics are pivotal indicators for favourable oral bioavailability and enhanced gastrointestinal (GI) absorption, positioning **MC1-7** as lead candidates with promising drug-like properties in ADME considerations.

**Table 1.** Molecular docking interactions between ligands (**MF1**, **MF2**, **MCl2**, **MCl3**) and 7X5N protein.

Ligand	Interacting Residue	Interaction Type	Distance (Å)
**MF1**	Leu117	Alkyl	5.23
	PRO352	Alkyl	5.00
	Val129	π-Alkyl	4.25
	His122	π-Alkyl	4.18
**MF2**	Leu117	Alkyl	5.22
	PRO352	Alkyl	4.90
	His122	π-Alkyl	4.40
**MCl2**	Hsd200	Hydrogen bond	3.18
	Arg359	Hydrogen bond	1.71
	Lys226	π-Cation	3.80
	His122	π–π Stacked	3.58
	Leu123, Ile29, Ala199, His122	Alkyl, π-Alkyl, π–π Stacked	Various
**MCl3**	Arg359	Hydrogen bond	2.50
	Ser31	C–H Bond	2.24
	Ser172	C–H Bond	2.91
	Lys226	π-Cation + π-Donor H-Bond	3.80, 2.69
	His122	π–π Stacked	3.79
	Leu123, Ile29, Ala199, His122	Alkyl, π-Alkyl, π–π Stacked	Various

Veber’s drug-likeness rule assesses the capacity of drugs to traverse the cellular membrane from the gastrointestinal tract by considering the number of rotatable bonds (Rb) and the topological polar surface area (TSPA). All synthesised compounds adhere to Veber’s guidelines, where the number of rotatable bonds (Rb) is required to be less than 10, and the TSPA is expected to be less than 140 Å, suggesting that they are likely to be orally bioavailable (Table 1S Supporting Information).

### Biological results

Menthol-based antimicrobials (**MC1-7**) were assessed for their antimicrobial activity against a diverse panel of bacteria (Table 2S Supporting Information). The results revealed a general lack of antimicrobial efficacy for **MC1-7**, as indicated by high MIC_50_ (Minimum Inhibitory Concentration required to inhibit the growth of 50% of the tested strains) values, largely equal to or exceeding 512 mg/L. However, within this group, certain derivatives – specifically **MC2**, **MC4**, and **MC5** – displayed slightly improved antimicrobial activity, particularly against *E. faecium*, with a MIC_50_ of 256 mg/L, in contrast to menthol (> 512 mg/L). This modest increase in efficacy suggests a potential specificity of these compounds towards *E. faecium*, although the overall activity of the **MC1-7** series remained limited.

**Table 2.** Summary of free energy landscape (FeL) and principal component analysis (PCA) for protein–ligand complexes.

Ligand	PC1 Range	PC2 Range	RMSD (nm)	Radius of Gyration (Rg, nm)	FeL Conformation Stability
**MF1**	−0.3 to 0.5	−0.4 to 0.4	0.06	2.165	Single stable conformation identified
**MF2**	−0.2 to 0.4	−0.4 to 0.4	–	–	Stable conformations observed
**MCl2**	−0.4 to 0.4	−0.4 to 0.4	–	–	More constrained and energetically favourable conformations
**MCl3**	−0.4 to 0.4	−0.4 to 0.6	–	–	More constrained and energetically favourable conformations

Halogenated menthol derivatives, including fluorinated (**MF1-4**), acid chloride derivatives (**MCl1-4**), and brominated (**MBr1-2**) compounds, were subsequently tested against the same bacterial panel (Table 3S Supporting Information). Interestingly, these derivatives exhibited enhanced antimicrobial activity, particularly against *E. faecalis* and *E. faecium* strains.

**Table 3.** MM/GBSA free binding energy components of ligands bound to 7X5N protein.

Energy (kcal/mol)	**MF1**	**MF2**	**MCl2**	**MCl3**
Δ_TOTAL_	−21.19	−25.99	−26.58	−26.52
Δ_VDWAALS_	−29.69	−32.71	−35.94	−37.28
ΔE_EL_	0.21	−18.16	−3.03	−4.58
ΔE_GB_	12.88	29.94	17.45	20.41
ΔE_SURF_	∼−5.00	∼−5.00	∼−5.00	∼−5.00
ΔG_GAS_	−29.47	−50.88	−38.97	−41.86
ΔG_SOLV_	8.28	24.88	12.39	15.34

Among them, **MF1** showed the most potent antimicrobial activity, with MIC values ranging from 8 to 64 mg/L against *E. faecium*, representing a significant improvement over menthol, with a five-fold reduction in MIC_50_. This marked reduction highlights the superior potency of **MF1** against this specific pathogen (Table 3S, Supporting Information). **MF2**, **MCl2**, and **MCl3** also showed improved activity (MIC < 256 mg/L) compared to menthol (MIC > 512 mg/L), but to a lesser extent than **MF1**. Although we did not directly test standard antibiotics in this study, literature data indicate that the MIC of ampicillin, the drug of choice for susceptible *E. faecium*, is typically around 1–16 mg/L, depending on the strain^29^. By comparison, our results show that **MF1** exhibits meaningful antimicrobial activity against *E. faecium*, supporting its potential as a lead compound for further development.

**Figure 1.**
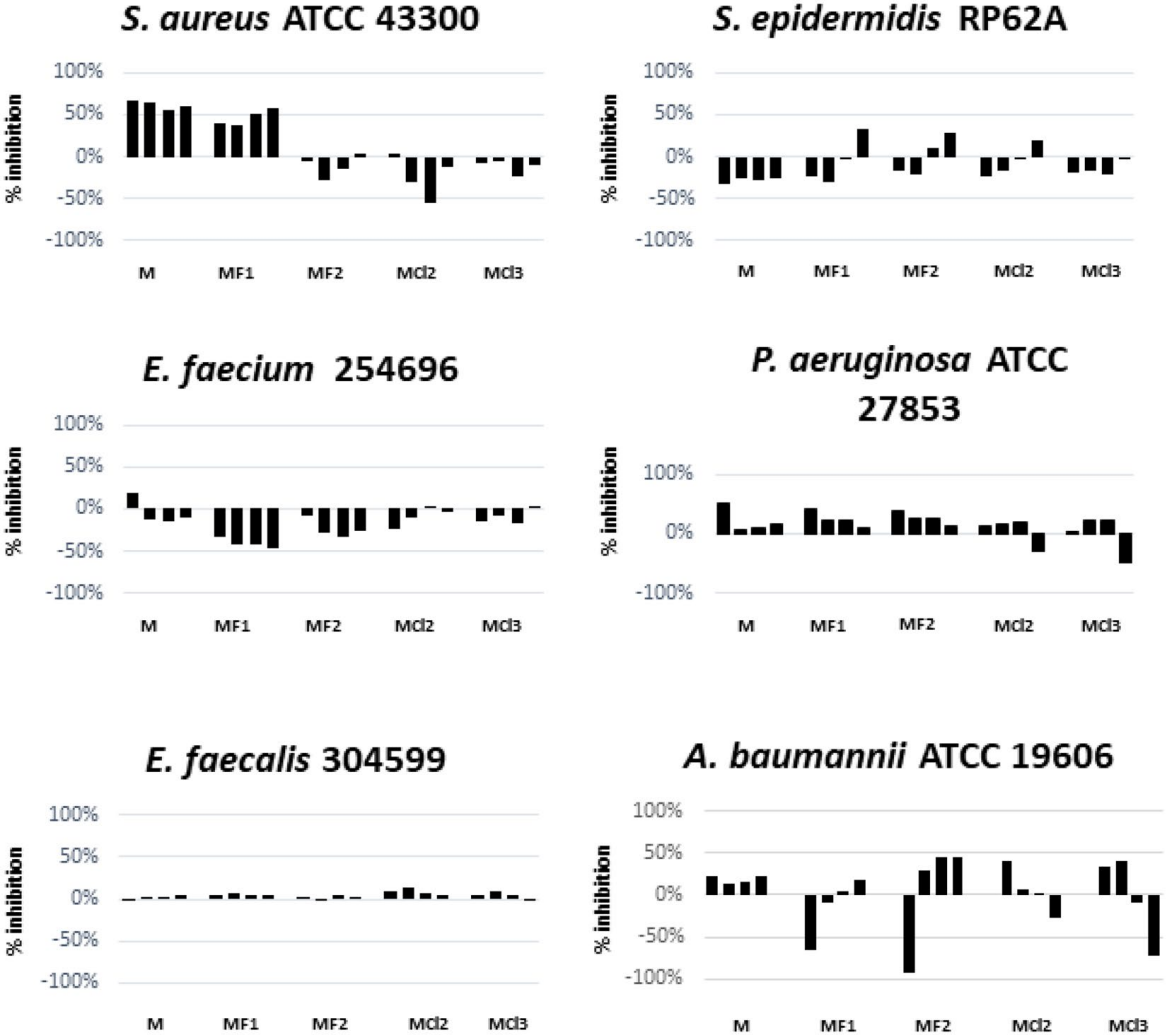
Biofilm inhibition activity of **MF1**, **MF2**, **MCl2**, and **MCl3** at subMICs concentrations (from 0.5 to 0.06 × MIC, respectively). Results are expressed as inhibition (%) of total biomass calculated as follows: % inhibition = (ODCTR-OD treated)/ODCTR x 100. Clinical and ATCC strains’ MIC values are indicated in the diagram below.

Based on these MIC results, **MF1**, **MF2**, **MCl2**, and **MCl3** were selected for further evaluation of their effects on biofilm biomass production by reference and clinical strains known to form biofilms (Figure 1). In the case of three Gram-positive species, the halogenated menthol derivatives exhibited species-specific effects. Notably, in *E. faecalis* 304599, no appreciable influence on biomass production was observed. In contrast, in *S. epidermidis* RP62A and *E. faecium* 254696, the derivatives showed their variable effects, including biofilm promotion, highlighting the complexity and the context dependency of their interactions with different bacterial species.

In *S. aureus* ATCC 43300, both menthol and **MF1** displayed notable biofilm-inhibitory activity, exceeding 50%, even at sub-inhibitory concentrations (subMIC). This important effect suggests the potential of these compounds to disrupt biofilm formation in *S. aureus*. Conversely, in *P. aeruginosa* ATCC 27853, **MF1** and **MF2** led to a progressive reduction in biomass with a dose-dependent effect. In *A. baumannii* ATCC 19606, responses varied considerably among compounds, with **MCl2** being the most effective, reducing biomass by up to 40% at the lowest subMIC concentrations tested (0.06 x MIC) (Figure 1). This heterogeneity in responses across bacterial species underscores the need for a tailored approach when considering the application of these menthol derivatives as antibiofilm agents.

**Figure 2.**
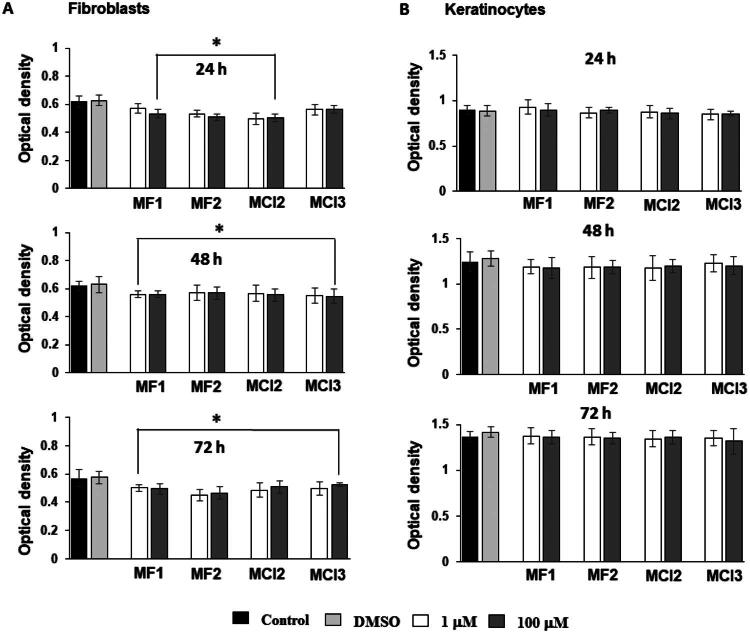
Effects of **MF1**, **MF2**, **MCl2**, and **MCl3** (1 μM and 100 μM) on human gingival fibroblasts and oral keratinocytes in cell viability. (A) Cell viability in fibroblasts assessed by MTT assay. (B) Cell viability in keratinocytes assessed by MTT assay. Control = non-treated cells; DMSO = cells treated with dimethyl sulfoxide (1 μL/mL). Data shown are representative of three separate experiments, and values are given as mean ± SD. Statistical analysis was performed using Student’s t-test. *p* > 0.05 vs. DMSO; *p < 0.05 vs. control DMSO.

Cytotoxicity assessment is essential for identifying cellular targets of novel menthol-based antimicrobials and guiding the development of agents that selectively target diseased cells while minimising effects on healthy ones^30^. Therefore, the cytotoxic potential of **MF1**, **MF2**, **MCl2**, and **MCl3** was assessed using human gingival fibroblasts and oral keratinocytes (Figure 2). According to MTT assay results, none of the four compounds induced significant changes in keratinocyte viability after 24, 48, and 72 h (*p* > 0.05) (Figure 2(B)). In contrast, a slight but statistically significant decrease in fibroblast viability was observed following treatment with **MF1**, **MF2**, and **MCl2** at concentrations of 1 and 100 µM at the same time point (*p* < 0.05) (Figure 2(A)). Notably, **MF1** at 1 µM did not cause a significant reduction in cell number (*p* > 0.05). **MCl3** induced a minor but statistically significant decrease in fibroblast viability after 48 and 72 h of exposure at both tested concentrations (Figure 2(A)).

**Figure 3.**
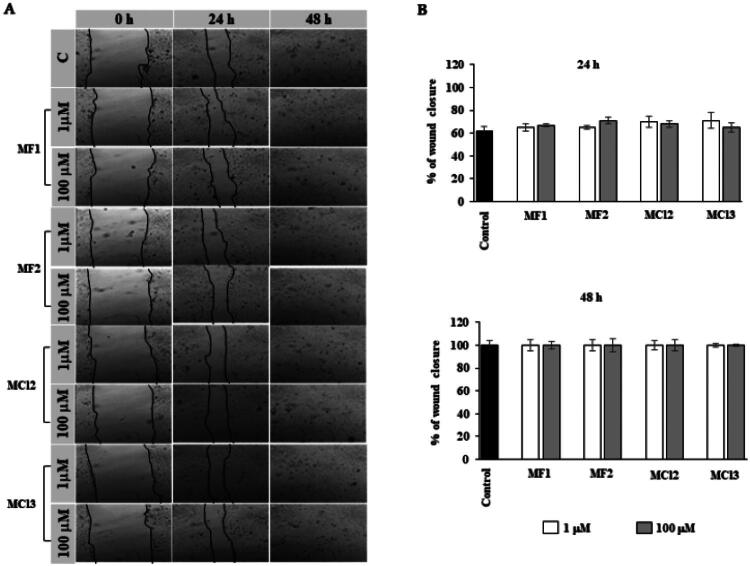
Effects of **MF1**, **MF2, MCl2**, and **MCl3** on human oral keratinocyte in the wound-healing migration assay. (A) Representative phase-contrast images of the wounds were taken at 0, 24, and 48 h (200X magnification). (B) Quantification of the percentage of closed wound area calculated by tracing the border of the wound using ImageJ software. Data represent the mean ± SD of three independent experiments.

**Figure 4.**
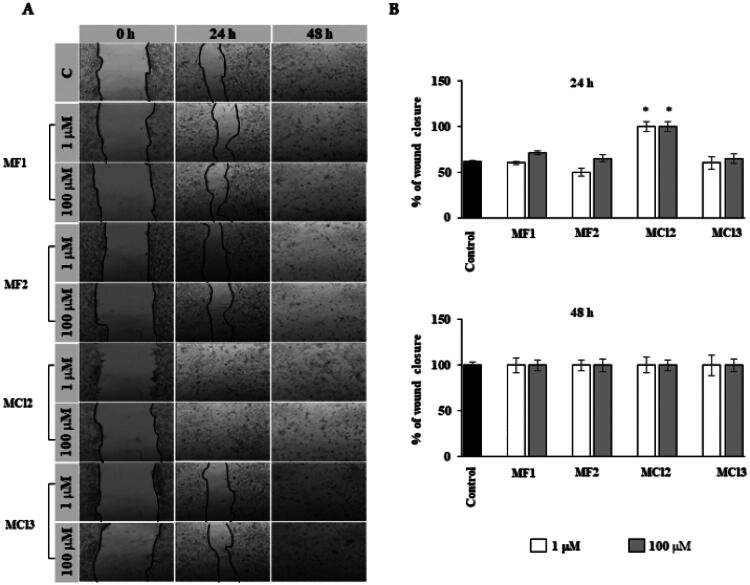
Effects of **MF1**, **MF2**, **MC12**, and **MC13** on human gingival fibroblasts in the wound-healing migration assay. (A) Representative phase-contrast images of the wounds were taken at 0, 24, and 48 h (200X magnification). (B) Quantification of the percentage of closed wound area calculated by tracing the border of the wound using ImageJ software. Data represent the mean ± SD of three independent experiments. *p < 0.001.

To investigate the effects of these compounds on wound healing, an *in vitro* scratch assay was performed using both human keratinocytes and fibroblasts (Figures 3 and 4, respectively)^31^. Wound area data were derived from scratch width measurements (in pixels) and converted to percentages to evaluate wound closure. Measurements at 24 and 48 h allowed for analysis of healing progression over time. Remarkably, all tested compounds (**MF1**, **MF2**, **MCl2**, and **MCl3**) at both concentrations (1 µM and 100 µM) significantly promoted cell migration and achieved complete wound closure (100%) within 48 h in both cell types, as depicted in Figures 3 and 4. These findings indicate a strong positive effect of the compounds on the wound-healing capacity of keratinocytes and fibroblasts.

Of particular note was the effect of **MCl2**, which achieved full wound closure in fibroblasts within just 24 h (*p* < 0.001), indicating a significantly accelerated healing response. These findings highlight the potential of **MCl2** as a promising agent for promoting fibroblast-mediated wound repair (Figure 4).

In summary, the *in vitro* scratch assay results confirmed that **MF1**, **MF2**, **MCl2**, and **MCl3** enhanced wound healing by stimulating cell migration and achieving full wound closure within the evaluated time frame. The better performance of **MCl2**, especially in accelerating wound closure in fibroblasts, highlights its potential as a promising candidate for further exploration in the context of wound healing applications, achieving complete closure within 24 h, whereas the other compounds require 48 h.

### Molecular docking analysis

**Figure 5.**
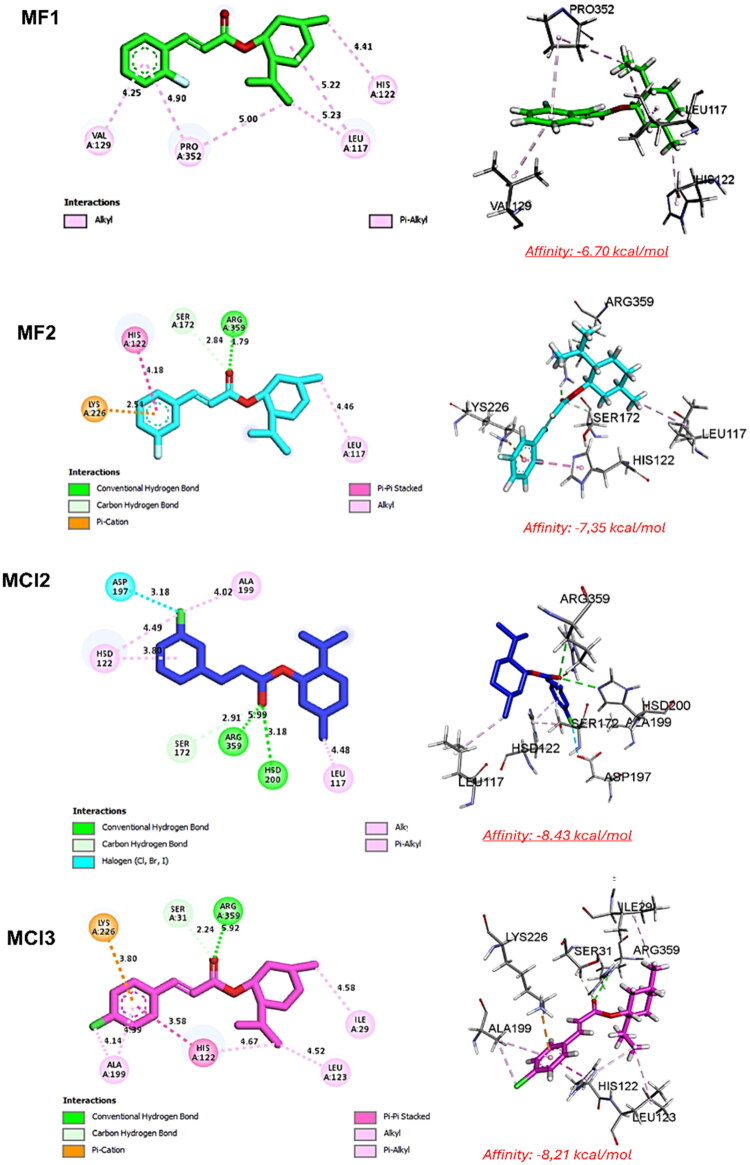
Docking plots for ligand–protein (7X5N) interactions.

Molecular docking was used to evaluate the interactions between the compounds under investigation and the selected protein. The Universal Force Field (UFF) was used to minimise energy, and Avogadro was used to generate the 3D structures for **MF1**, **MF2**, **MCl2**, and **MCl3**. Molecular docking against the 7X5N protein revealed key non-covalent interactions (see Table 1 and Figure 5). **MF1** and **MF2** formed alkyl and π-alkyl interactions with residues such as Leu117, Pro352, Val129, and His122. In contrast, **MCl2** and **MCl3** exhibited strong hydrogen bonds (notably with Hsd200 and Arg359), as well as π-cation, π-donor hydrogen bonds, and π-π stacking interactions involving residues like Lys226 and His122. The presence of stronger and more diverse interactions in **MCl2** and **MCl3** supports their higher binding affinity to 7X5N, in agreement with their superior inhibitory activity against *E. faecium*.

### Molecular dynamics analysis

**Figure 6.**
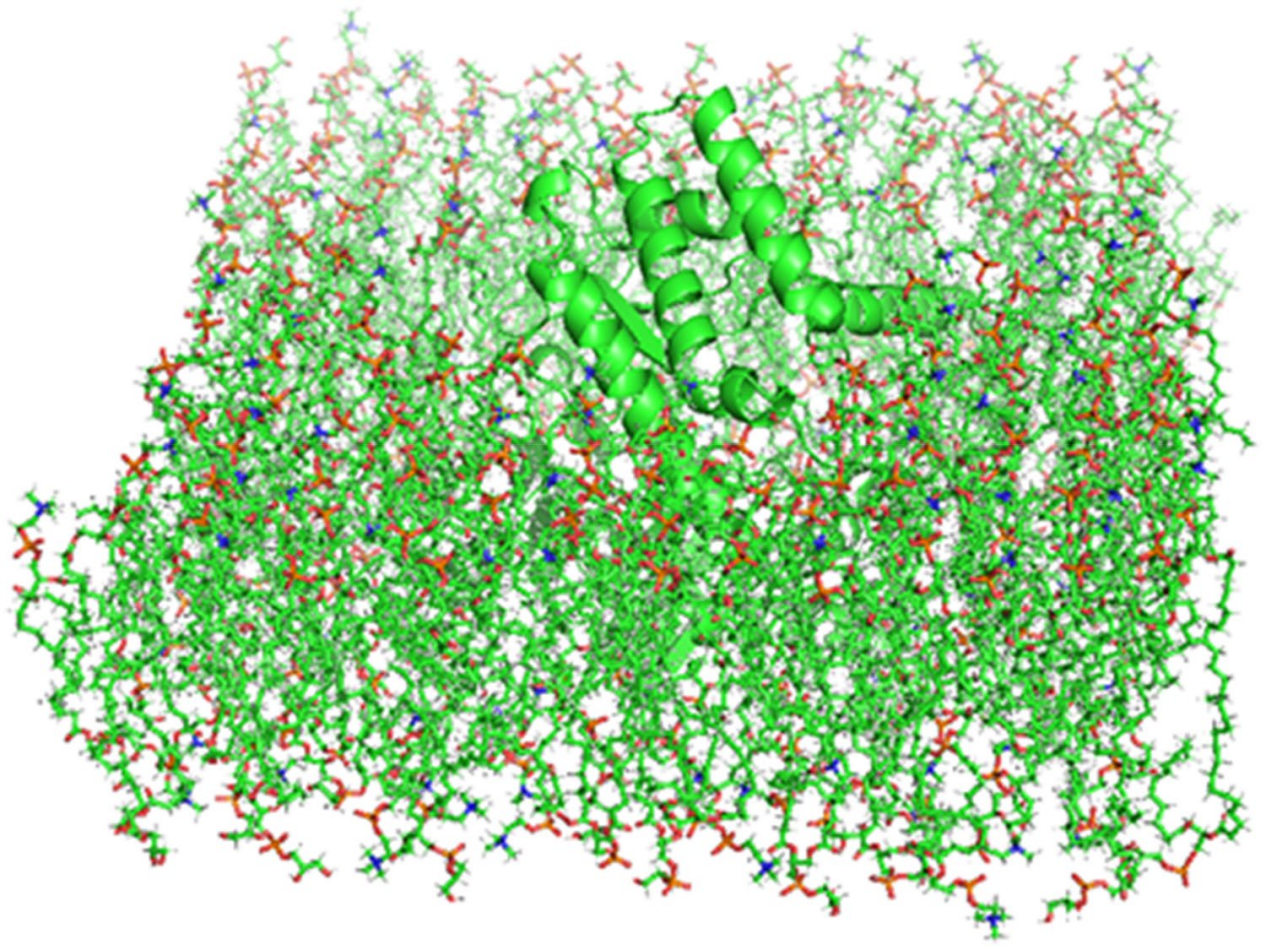
The membrane assembly includes the integration of the target protein.

**Figure 7.**
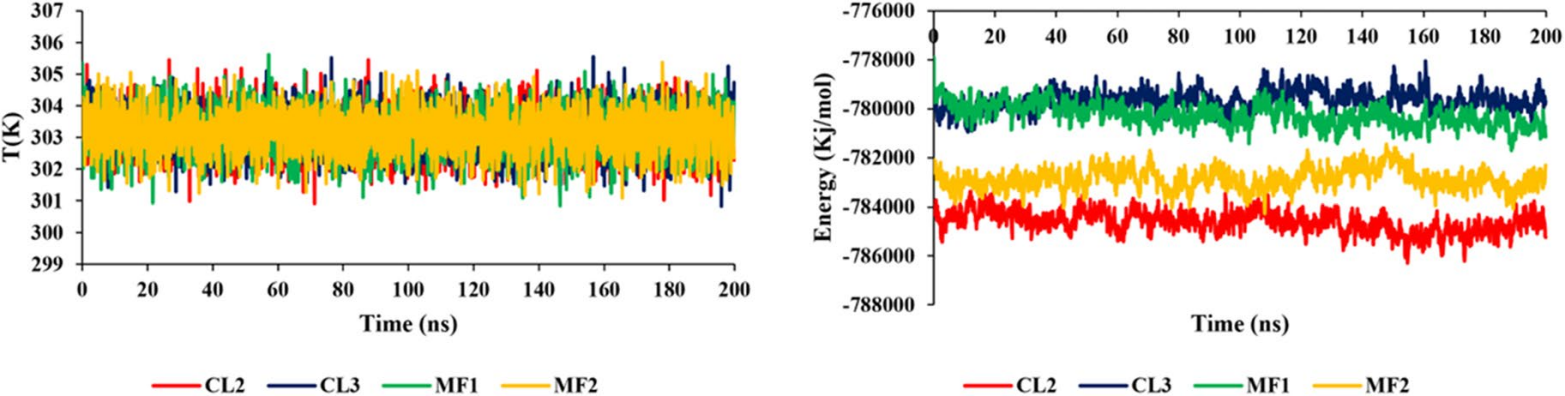
Temperature and energy variation during the simulation.

**Figure 8.**
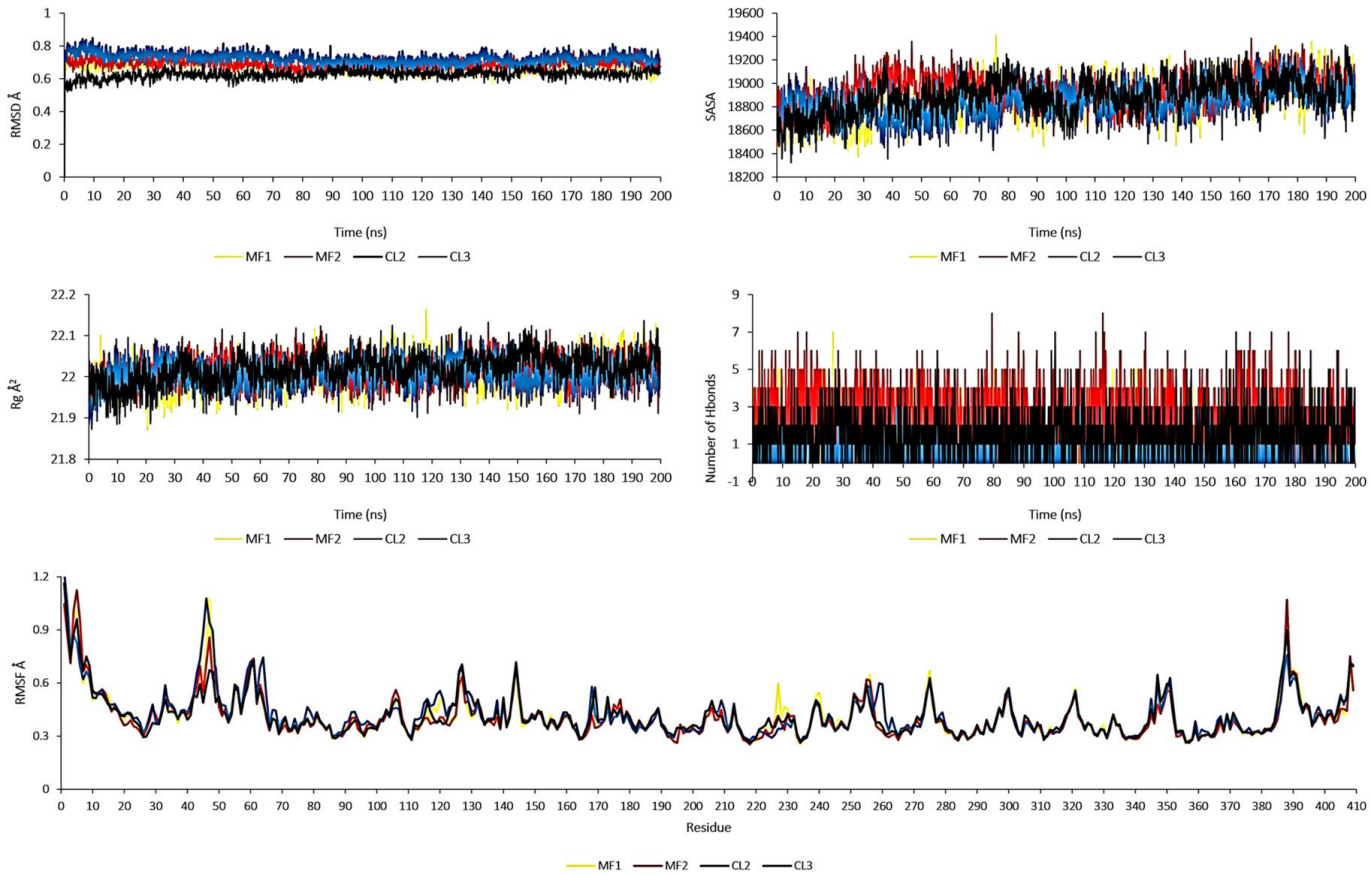
RMSD, SASA, Rg, and RMSF plots.

The simulations of molecular dynamics were used to assess how four chemicals (**MF1**, **MF2**, **MCl2**, and **MCl3**) interacted with the 7X5N protein, a crucial enzyme in the production of amino acids that is necessary for the survival and proliferation of *E. faecium*. To create a realistic biological environment, a phospholipid bilayer membrane model composed of DMPC and DMPG was constructed, incorporating the 7X5N protein within the membrane assembly (Figure 6). The simulations ran for 200 ns, providing insights into system stability through monitoring total energy and temperature variations. All compounds exhibited consistently negative total energy values throughout the simulation, indicating stable and favourable interactions with the protein-membrane complex. The temperature remained steady around 303 K, demonstrating thermal equilibrium during the simulation period (Figure 7). Root Mean Square Deviation (RMSD) analysis showed that all membrane-bound complexes were highly stable, with RMSD values consistently ranging from 0.6 to 0.8 Å, while Solvent Accessible Surface Area (SASA) values between 18,600 to 19,200 Å^2^ confirmed compactness and structural integrity in the solvent environment. Hydrogen bond analysis revealed ligand–protein interactions ranging from 1 to 8 bonds, indicating strong affinity within the membrane, and Root Mean Square Fluctuation (RMSF) analysis validated the stability of individual amino acid residues, corroborating the RMSD results. These comprehensive dynamics data (Figure 8) confirm that **MF1**, **MF2**, **MCl2**, and **MCl3** form stable, energetically favourable complexes with 7X5N in a lipid bilayer context, supporting their potential as effective antimicrobial agents targeting *E. faecium* through specific membrane and protein interactions.

### Free energy landscape (FeL) and principal component analysis (PCA)

**Figure 9.**
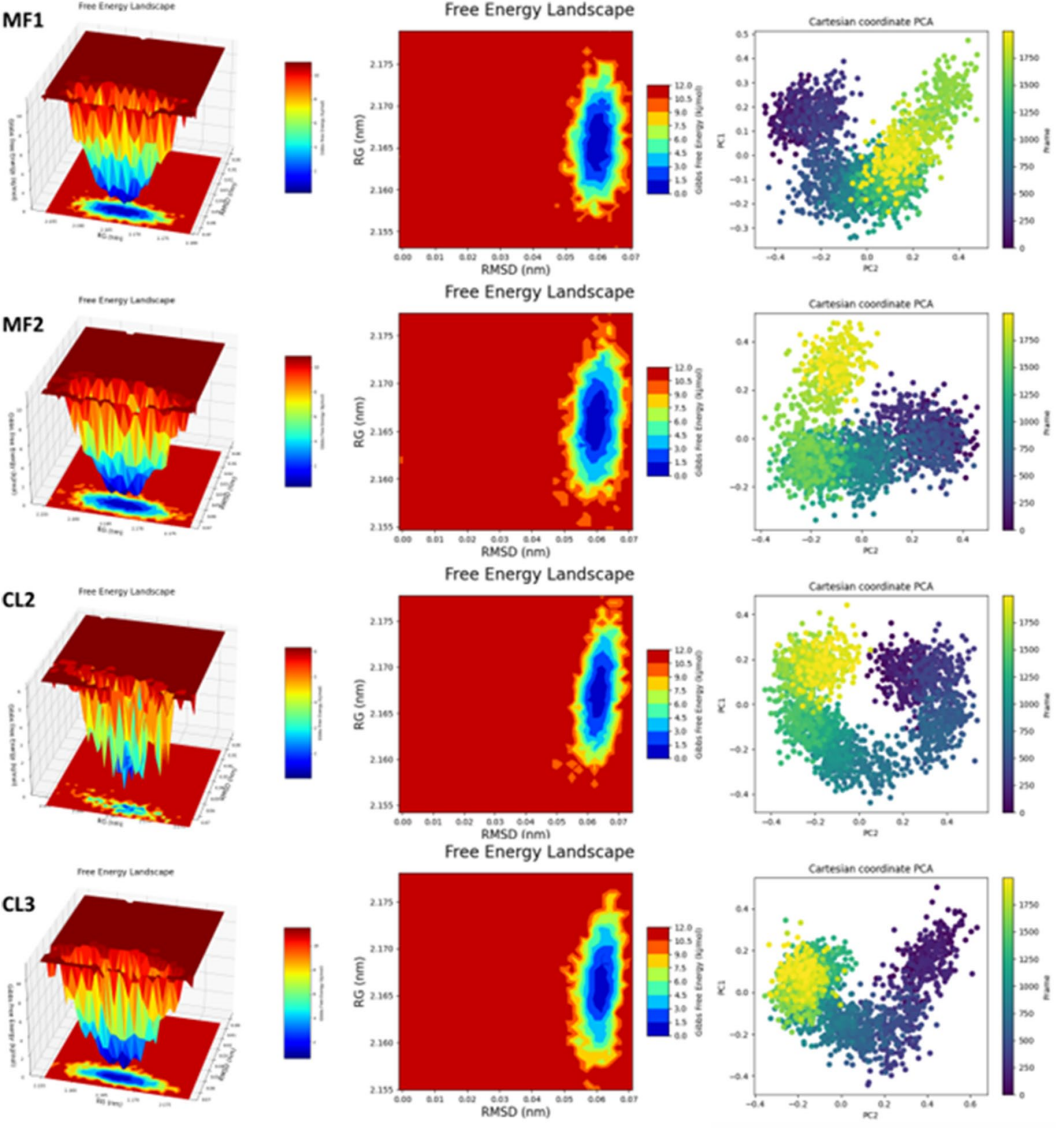
PCA and Fel plots.

**Figure 10.**
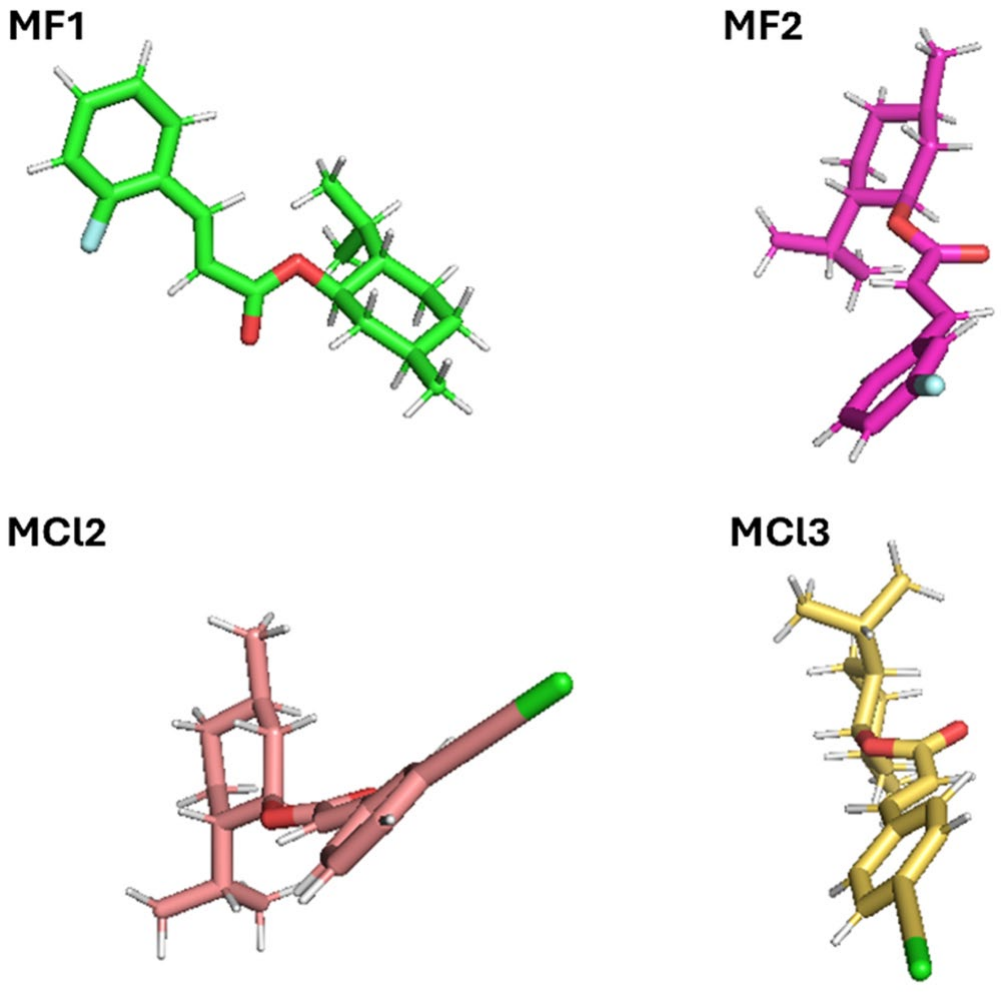
The best conformation corresponds to the minimum energy.

The FeLand PCA analyses provided in-depth insights into the conformational dynamics of the protein–ligand complexes (Table 2). PCA results (Figure 9) revealed distinct diffusion patterns for **MF1**, **MF2**, **MCl2**, and **MCl3** along principal components PC1 and PC2, indicating unique dynamic behaviours for each complex. FeL plots (Figure 9) showed well-defined energy minima corresponding to stable conformations. Among them, **MF1** exhibited high structural stability (RMSD: 0.06 nm; Rg: 2.165 nm). **MCl2** and **MCl3** demonstrated more constrained motions and deeper energy basins, supporting their stronger binding affinities and higher biological activities. As depicted in Figure 10, these stable conformations correspond to the lowest-energy states observed during molecular dynamics simulations.

### Free binding energy (MM/GBSA)

In computational chemistry, the Molecular Mechanics/Generalized Born Surface Area (MM/GBSA) approach is frequently employed to estimate the binding free energy of ligands to their target proteins. Analysis of the MM/GBSA results presented in Table 3 (Supporting Information). MM/GBSA calculations were performed to estimate the binding free energies of **MF1**, **MF2**, **MCl2**, and **MCl3** with the *E. faecium* target (Table 3, Figure 10). **MCl2** and **MCl3** displayed the most favourable total binding energies (ΔTOTAL: −26.58 and −26.52 kcal/mol, respectively), followed by **MF2** (–25.99 kcal/mol), while **MF1** showed the least favourable profile (–21.19 kcal/mol). **MCl3** and **MCl2** exhibited the strongest van der Waals interactions, supporting their stable fit in the binding pocket. **MF2** demonstrated the most favourable electrostatic interactions (ΔEEL: −18.16 kcal/mol), although it also had the highest solvation penalty. Across all ligands, non-polar solvation energy (ΔESURF) contributed modestly, while gas-phase interaction energy (ΔGGAS) was most favourable for **MF2**. Overall, the data confirm that **MCl2** and **MCl3** exhibit the strongest binding affinities, which is consistent with the docking results and further supports their potential as potent *E. faecium* inhibitors.

## Discussion

In this paper, we report the synthesis of seventeen menthol derivatives containing the cinnamoyl scaffold. Antibacterial assays performed on these esters identified **MF1** – bearing a 2-fluoro cinnamic acid moiety - as the most promising candidate. It showed selective antimicrobial activity against *E. faecium* (MIC_50%_ = 16 mg/L) and no cytotoxicity on human gingival fibroblasts and oral keratinocytes at both tested concentrations (1 and 100 µM). However, increased lipophilicity due to the incorporation of chlorine or bromine led to a reduced activity (MIC_50%_ = 128 mg/L), though still superior to menthol (MIC_50%_ > 512 mg/L*).* Historically, *E. faecium* has been considered less pathogenic than *E. faecalis*, but its clinical significance has increased in recent years due to its involvement in healthcare-associated infections^32^. Infections caused by this bacterium can occur in the urinary tract, bloodstream, intra-abdominal areas, and wounds. Recently, *E. faecium* has developed resistance to multiple antibiotics, including vancomycin, which is often considered the drug of last resort for treating enterococcal infections^33^.

Literature reports indicate that fluorinated cinnamic acid derivatives possess cytoprotective, fungitoxic, and anti-convulsant agents in mammals^34^^,^^35^. This study demonstrated that introducing fluorine into the cinnamic scaffold enhances both antimicrobial and antibiofilm activity. Increased hydrophobicity resulting from fluorination likely enhances membrane permeability and binding to microbial targets. Fluorine is widely employed in the pharmaceutical industry to enhance the pharmacokinetic profiles of drugs, with over 150 fluorinated drugs currently approved for clinical use. Beyond small molecules, fluorination has been utilised to modulate the physical and biological properties of peptides and proteins^36^. Fluorination strategies have also been applied to optimise the biological activity of therapeutic peptides, including glucagon-like peptide-1 (GLP-1) and antimicrobial peptides like buforin-2 and Jelleine-I^37^.

**MF1** also effectively reduced biomass production in two clinically relevant strains: *Staphylococcus aureus* ATCC 43300 and *Pseudomonas aeruginosa* ATCC 27853. Specifically, **MF1** decreased biomass by 50% in *S. aureus* and by 20% in *P. aeruginosa* at sub-MIC concentrations. This is highly relevant, as biofilms - which are communities of bacteria adhering to surfaces and surrounded by a protective matrix - are notoriously resistant to treatment and are common in persistent hospital-acquired infections^38^. The ability of **MF1** to reduce biofilm biomass in both *S. aureus* and *P. aeruginosa* highlights its potential as a broad-spectrum antimicrobial agent, particularly in clinical settings where these bacteria are prevalent. These pathogens are major causes of healthcare-associated infections, including surgical site infections, ventilator-associated pneumonia, and catheter-associated urinary tract infections^39^.

Biological data demonstrated that fluorinated cinnamic acid derivatives exhibited lower minimum inhibitory concentrations (MICs) and higher biofilm inhibition compared to their non-fluorinated counterpart. Mechanistic insights suggest that the electronegativity of fluorine and its influence on molecular electron distribution may enhance binding to microbial enzymes and structural components, thereby disrupting critical biological processes within the microorganisms.

Conversely, **MCl2** - obtained via esterification of menthol with 2-chloro-cinnamic – demonstrated notable wound-healing properties, especially on human gingival fibroblasts, where it achieved full wound closure within 24 h, in contrast to 48 h for other menthol-based antimicrobials. This rapid healing effect represents an additional therapeutic advantage, particularly for infected wounds requiring both antimicrobial treatment and tissue regeneration. The ability of **MCl2** to accelerate wound closure may reduce recovery times and the risk of secondary infections, making it an attractive candidate for wound care formulations. It is well-known that chlorinated compounds (hypochlorous acid, sodium hypochlorite, or N-chloroaurine) are widely utilised in clinical settings for their potent antimicrobial properties, especially in managing acute and chronic wounds. These agents offer broad-spectrum activity against bacteria, fungi, and viruses, and are particularly effective in disrupting biofilms—a major challenge in chronic wound infections.

In both menthol-based antimicrobials **MF1** and **MCl2**, the hydroxyl group of menthol was protected through derivatization with halogenated cinnamoyl moieties, which may reduce hepatic metabolism and enhance pharmacological stability.

To elucidate the mechanism underlying the enhanced antibacterial and antibiofilm properties of **MF1** and **MCl2**, molecular docking and dynamics simulations were performed. Docking studies revealed strong binding affinities to the *E. faecium* target protein (7X5N), with **MCl2** and its homolog **MCl3** forming robust hydrogen bonds that correlated with their greater inhibitory activity. The stability of these complexes was further supported by molecular dynamics simulations, which revealed minimal conformational variations and persistent interactions. **MF1** exhibited the lowest MIC values (8–64 mg/L) against *E. faecium*, whereas **MCl2** displayed dual efficacy by reducing biofilm formation (40% in *A. baumannii*) and accelerating wound healing. These computational results are consistent with the experimental observations. The simulations further clarified why brominated derivatives exhibited reduced activity, as the bulkier halogens interfered with optimal binding. By demonstrating how fluorination and chlorination enhance antibacterial potency, biofilm disruption, and treatment stability, the combined computational and experimental findings collectively validate the structure–activity relationship.

## Conclusions

The findings of this study highlight the potential of halogenated cinnamic acid–menthol derivatives as promising antimicrobial agents, offering additional therapeutic benefits, including biofilm disruption and wound healing. The introduction of fluorine into the cinnamic scaffold enhances the antimicrobial efficacy of derivative **MF1**, making it a promising candidate for further development in the fight against antibiotic-resistant infections and healthcare-associated biofilms. The introduction of chlorine in the cinnamic scaffold **MCl2** represents an important advancement in developing multifunctional antimicrobial agents capable of addressing the challenges posed by resistant pathogens and promoting efficient wound healing. Overall, this research highlights the potential of these novel antimicrobials as promising candidates for innovative strategies aimed at combating common infections that have become difficult to eradicate due to antimicrobial resistance. Further studies are necessary to investigate these effects on animal models.

## Supplementary Material

ANONYMOUS SUPPORTING INFORMATION.pdf

Highlights JEIMC.docx

SI_Editable_Tables (1).docx

## Data Availability

The data are available upon request from the corresponding author.
