# Supplementary material for: Advancing infection therapy: the role of novel menthol-based antimicrobials
Source: J Enzyme Inhib Med Chem. 2026 Jan 6;41(1):2596488. doi: 10.1080/14756366.2025.2596488 (PMC12777765; doi:10.1080/14756366.2025.2596488)
Supplement: ANONYMOUS SUPPORTING INFORMATION.pdf [file IENZ_A_2596488_SM9674.pdf]

SUPPORTING INFORMATION

Advancing infection therapy: the role of novel menthol-based antimicrobials

INDEX

A)	ADME STUDIES	p. 2
B)	BIOLOGICAL STUDIES	p. 3
C)	COMPUTATIONAL STUDIES	p. 5
D)	NMR and HR-MS SPECTRA, and HPLC CHROMATOGRAMS OF FINAL COMPOUNDS	p. 7

A) ADME STUDY

Table 1 SI. Drug likeness properties[a]

Cpd	Water Solubility	Lipinski rule				Lipinski Violations	TSPA	MR	Rb
		MW	Log P	HBA	HBD				
MC1	$1.26*10^{-2}$	286.41	4.60	2	0	0	26.30	88.58	5
MC2	$2.70*10^{-2}$	330.42	4.30	4	0	0	44.76	94.64	5
MC3	$9.11*10^{-3}$	346.46	4.50	4	0	0	44.76	101.57	7
MC4	$1.07*10^{-2}$	316.43	4.60	3	0	0	35.53	95.07	6
MC5	$1.07*10^{-2}$	316.43	4.51	3	0	0	35.53	95.07	6
MC6	$1.07*10^{-2}$	316.43	4.60	3	0	0	35.53	95.07	6
MC7	$7.76*10^{-3}$	376.49	4.57	5	0	0	53.99	108.06	8
MBr1	$2.51*10^{-3}$	365.30	5.08	2	0	1	26.30	96.28	5
MBr2	$2.51*10^{-3}$	365.30	5.08	2	0	1	26.30	96.28	5
MF1	$7.17*10^{-3}$	304.40	4.77	3	0	1	26.30	88.54	5
MF2	$7.17*10^{-3}$	304.40	4.86	3	0	1	26.30	88.54	5
MF3	$7.17*10^{-3}$	304.40	4.86	3	0	1	26.30	88.54	5
MF4	$4.08*10^{-3}$	322.39	5.08	4	0	1	26.30	88.50	5
MCl1	$3.55*10^{-3}$	320.85	4.98	2	0	1	26.30	93.59	5
MCl2	$3.55*10^{-3}$	320.85	5.08	2	0	1	26.30	93.59	5
MCl3	$3.55*10^{-3}$	320.85	5.08	2	0	1	26.30	93.59	5
MCl4	$9.99*10^{-4}$	355.30	5.52	2	0	1	26.30	98.60	5

[a] Prediction SwissADME platforms.

B) BIOLOGICAL STUDIES

Table 2 SI. Antimicrobial activity of menthol-based antimicrobials against Gram-positive and Gram-negative species.

Strain (n) [a]	MIC (mg/L) [b]	Menthol	MC1	MC2	MC3	MC4	MC5	MC6	MC7
S. aureus	range	>512	>512	>512	>512	>512	>512	>512	>512
(10)	50%	>512	>512	>512	>512	>512	>512	>512	>512
	90%	>512	>512	>512	>512	>512	>512	>512	>512
S. epidermidis	range	>512	>512	512->512	>512	512->512	512->512	512->512	>512
(8)	50%	>512	>512	512	>512	512	512	512	>512
	90%	>512	>512	>512	>512	>512	>512	>512	>512
E. faecalis	range	>512	512->512	>512	>512	512->512	>512	>512	>512
(8)	50%	>512	512	>512	>512	512	>512	>512	>512
	90%	>512	>512	>512	>512	>512	>512	>512	>512
E. faecium	range	>512	64->512	16-512	512	16-512	64->512	128->512	512
(10)	50%	>512	512	256	512	256	256	512	512
	90%	>512	>512	512	512	512	512	512	512
E. coli	range	>512	>512	>512	>512	>512	>512	>512	>512
(8)	50%	>512	>512	>512	>512	>512	>512	>512	>512
	90%	>512	>512	>512	>512	>512	>512	>512	>512
K. pneumoniae	range	>512	>512	>512	>512	>512	>512	>512	>512
(8)	50%	>512	>512	>512	>512	>512	>512	>512	>512
	90%	>512	>512	>512	>512	>512	>512	>512	>512
P. aeruginosa	range	>512	>512	>512	>512	>512	>512	>512	>512
(8)	50%	>512	>512	>512	>512	>512	>512	>512	>512
	90%	>512	>512	>512	>512	>512	>512	>512	>512
A. baumannii	range	512->512	>512	>512	>512	>512	>512	>512	>512
(10)	50%	512	>512	>512	>512	>512	>512	>512	>512
	90%	>512	>512	>512	>512	>512	>512	>512	>512
Enterobacter spp.	range	>512	>512	>512	>512	>512	>512	>512	>512
(10)	50%	>512	>512	>512	>512	>512	>512	>512	>512
	90%	>512	>512	>512	>512	>512	>512	>512	>512

[a] Including ATCC reference strains (n. 10); [b] 50% and 90%, MICs at which 50% and 90% of isolates are inhibited, respectively.

Table 3 SI. Antimicrobial activity of halogenated menthol derivatives.

Strain (n)[a]	MIC (mg/L) [b]	Menthol	MF1	MF2	MF3	MF4	MBr1	MBr2	MCl1	MCl2	MCl3	MCl4
S. aureus	range	>512	>512	>512	>512	>512	>512	>512	>512	>512	>512	>512
(10)	50%	>512	>512	>512	>512	>512	>512	>512	>512	>512	>512	>512
	90%	>512	>512	>512	>512	>512	>512	>512	>512	>512	>512	>512
S. epidermidis	range	>512	>512	>512	>512	>512	>512	>512	>512	>512	>512	>512
(8)	50%	>512	>512	>512	>512	>512	>512	>512	>512	>512	>512	>512
	90%	>512	>512	>512	>512	>512	>512	>512	>512	>512	>512	>512
E. faecalis	range	>512	64->512	64->512	64->512	64->512	64->512	128->512	64->512	64->512	64->512	64->512
(8)	50%	>512	>512	>512	>512	>512	512	512	512	>512	>512	>512
	90%	>512	>512	>512	>512	>512	>512	>512	>512	>512	>512	>512
E. faecium	range	>512	8-64	128-256	64-256	64-256	32-512	256-512	64-256	32-128	32-128	256-512
(10)	50%	>512	16	128	128	128	256	256	128	128	128	256
	90%	>512	32	128	128	128	512	256	256	128	128	256
E. coli	range	>512	>512	>512	>512	>512	>512	>512	>512	>512	>512	>512
(8)	50%	>512	>512	>512	>512	>512	>512	>512	>512	>512	>512	>512
	90%	>512	>512	>512	>512	>512	>512	>512	>512	>512	>512	>512
K. pneumoniae	range	>512	>512	>512	>512	>512	>512	>512	>512	>512	>512	>512
(8)	50%	>512	>512	>512	>512	>512	>512	>512	>512	>512	>512	>512
	90%	>512	>512	>512	>512	>512	>512	>512	>512	>512	>512	>512
P. aeruginosa	range	>512	512->512	>512	>512	>512	512->512	>512	512->512	>512	>512	>512
(8)	50%	>512	>512	>512	>512	>512	>512	>512	>512	>512	>512	>512
	90%	>512	>512	>512	>512	>512	>512	>512	>512	>512	>512	>512
A. baumannii	range	512->512	512->512	512->512	512->512	512->512	512->512	>512	512->512	512->512	512->512	512->512
(10)	50%	512	512	512	512	512	512	>512	512	512	512	512
	90%	>512	>512	>512	>512	>512	>512	>512	>512	>512	>512	>512
Enterobacter spp.	range	>512	>512	>512	>512	>512	>512	>512	>512	>512	>512	>512
(10)	50%	>512	>512	>512	>512	>512	>512	>512	>512	>512	>512	>512

90%	>512	>512	>512	>512	>512	>512	>512	>512	>512	>512	>512

[a]Including ATCC reference strains (n. 10); [b]50% and 90%, MICs at which 50% and 90% of isolates are inhibited, respectively.

C) COMPUTATIONAL STUDIES

Table 4 SI. Binding affinities in Kcal/mol.

MF1	MF2	MCl2	MCl3
-6.70	-7,35	-8.43	-8.21

Table 5 SI. Average Energy and Temperature for **MF1**, **MF2**, **MCl2**, and **MCl3.**

Average	Energy	Temperature
MF1	-782671	303.182
MF2	-783064	303.181
MCl2	-779848	303.181
MCl3	-784730	303.182

Molecular Mechanics (MM)/Generalized Born Surface Area (GSBA) analysis

The MM/GBSA analysis aims to estimate the overall binding free energy of a biomolecular complex. The MM/GBSA results for the **MF1**, **MF2**, **MCl2**, and **MCl3** provided crucial insights into binding and solvation energies, which are vital for understanding the interaction between these molecules and a protein (Table 6 Supporting Information).

ΔVDWAALS (Van der Waals Energy): This component measures the interaction between protein and ligand atoms due to Van der Waals forces. A trend of decreasing ΔVDWAALS values is observed when moving from **MF1** to **MCl3**, suggesting that Van der Waals interactions become more favorable from **MF1** to **MCl3**.

ΔEEL (Electrostatic Energy): This component quantifies the electrostatic interaction between partial charges of protein and ligand atoms. MF1 exhibits a positive contribution to binding energy, indicating a favorable electrostatic interaction, while for MF2, MCl2, and MCl3, the values are negative, suggesting an unfavorable contribution to binding energy.

ΔEGB (Born Solvated Energy): This represents the solvation energy calculated using the Born model to estimate the solvent effect. ΔEGB values increase from MF1 to MCl3, indicating a progressively positive contribution to binding energy due to solvation.

ΔESURF (Surface Energy): This component accounts for interactions with the solvent associated with the protein and ligand surfaces. Values are relatively consistent across different molecules, indicating that surface energy has minimal impact on binding energy variations.

ΔGGAS (Gas Phase Free Energy): It is the sum of Van der Waals and electrostatic energies in the gas phase (without solvent). ΔGGAS values decrease from MF1 to MCl3, suggesting that gas-phase energy becomes more favorable.

ΔGSOLV (Solvation Energy): This is the sum of Born and surface solvation energies, representing the solvent's effect on binding energy. Values increase from MF1 to MCl3, indicating a progressively positive contribution due to solvation.

ΔTOTAL (Total Energy): It is the sum of all the previous components. Values are negative for all molecules, indicating that the total energy favors the formation of the bond between the protein and ligands. However, ΔTOTAL values are more negative for MCl2 and MCl3 compared to MF1 and MF2, suggesting that MCl2 and MCl3 have an overall more favorable binding energy, this is consistent with the affinities obtained through molecular docking.

Table 6 SI. Energy components (Kcal/mol/protein-ligand) for MF1, MF2, MCl2, and MCl3.

**Delta Energy (Kcal/mol)**	MF1	MF2	MCl2	MCl3
ΔVDWAALS	-29.69	-32.71	-35.94	-37.28
ΔEEL	0.21	-18.16	-3.03	-4.58
ΔEGB	12.88	29.94	17.45	20.41
ΔESURF	-4.59	-5.06	-5.06	-5.07
ΔGGAS	-29.47	-50.88	-38.97	-41.86
ΔGSOLV	8.28	24.88	12.39	15.34
ΔTOTAL	-21.19	-25.99	-26.58	-26.52

HC1

Agilent Technologies

Sample Name:
MC1
Data Collected on:
m300-mercury300
Archive directory:
/home/caccia/vnmrsys/data
Sample directory:
OLP-RA_MARI1_20211021_01
FidFile: PROTON

Pulse Sequence: PROTON (s2pul)
Solvent: cdcl3
Data collected on: Mar 11 2022

Operator: caccia

Relax. delay 1.000 sec
Pulse 45.0 degrees
Acq. time 1.706 sec
Width 4803.1 Hz
32 repetitions
OBSERVE H1, 300.1976543 MHz
DATA PROCESSING
FT size 16384
Total time 1 min 29 sec

7

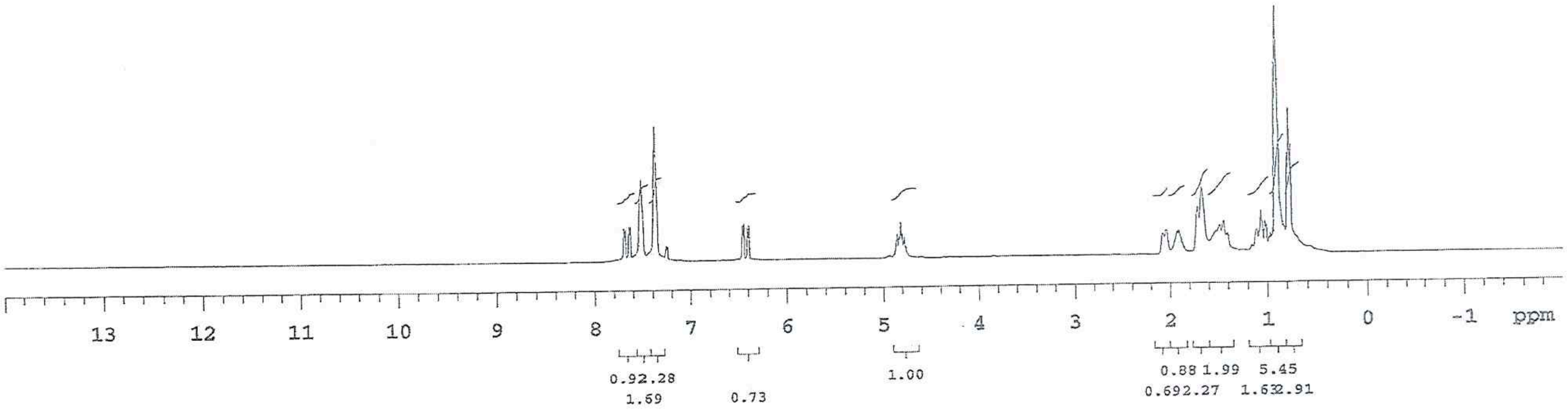

MC1

Gradient Shimming

Sample Name:
mc1_25-35
Data Collected on:
m300-mercury300
Archive directory:
/export/home/chempack/vnmrsys/data
Sample directory:

FidFile: CARBON

Pulse Sequence: CARBON (s2pul)
Solvent: cdcl3
Data collected on: Mar 2 2021

Operator: caccia

Relax. delay 1.000 sec
Pulse 45.0 degrees
Acq. time 0.868 sec
Width 18867.9 Hz
2000 repetitions
OBSERVE C13, 75.4847602 MHz
DECOUPLE H1, 300.1991980 MHz
Power 38 dB
continuously on
WALTZ-16 modulated
DATA PROCESSING
Line broadening 0.5 Hz
FT size 32768
Total time 1 hr, 4 min

Agilent Technologies

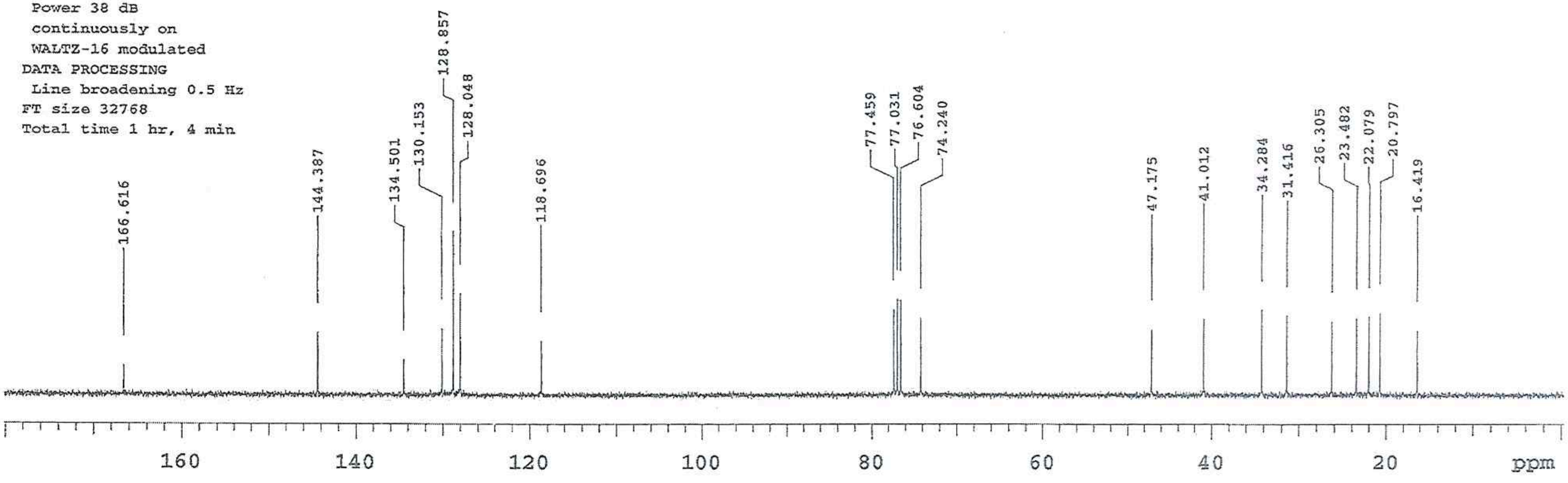

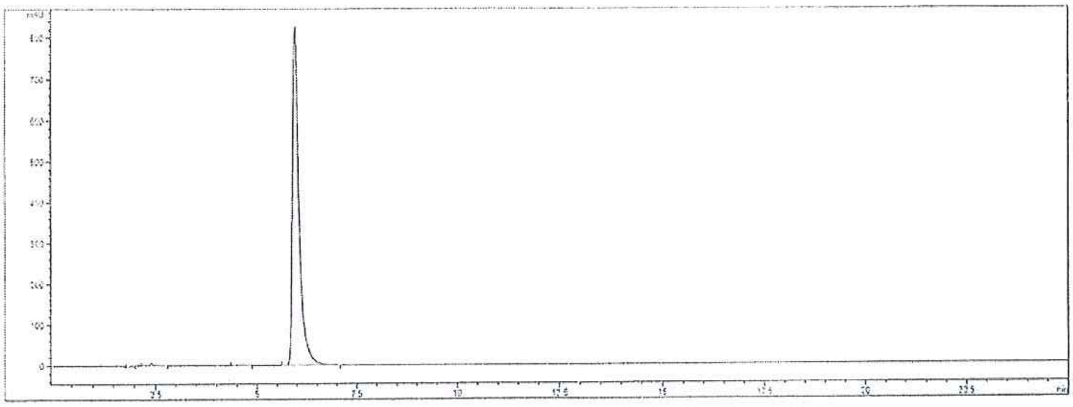

Compound	Retention time	Area%
MC1	5.96	>99

High Resolution Mass Spectrometry Analysis

MC1 (286.19)

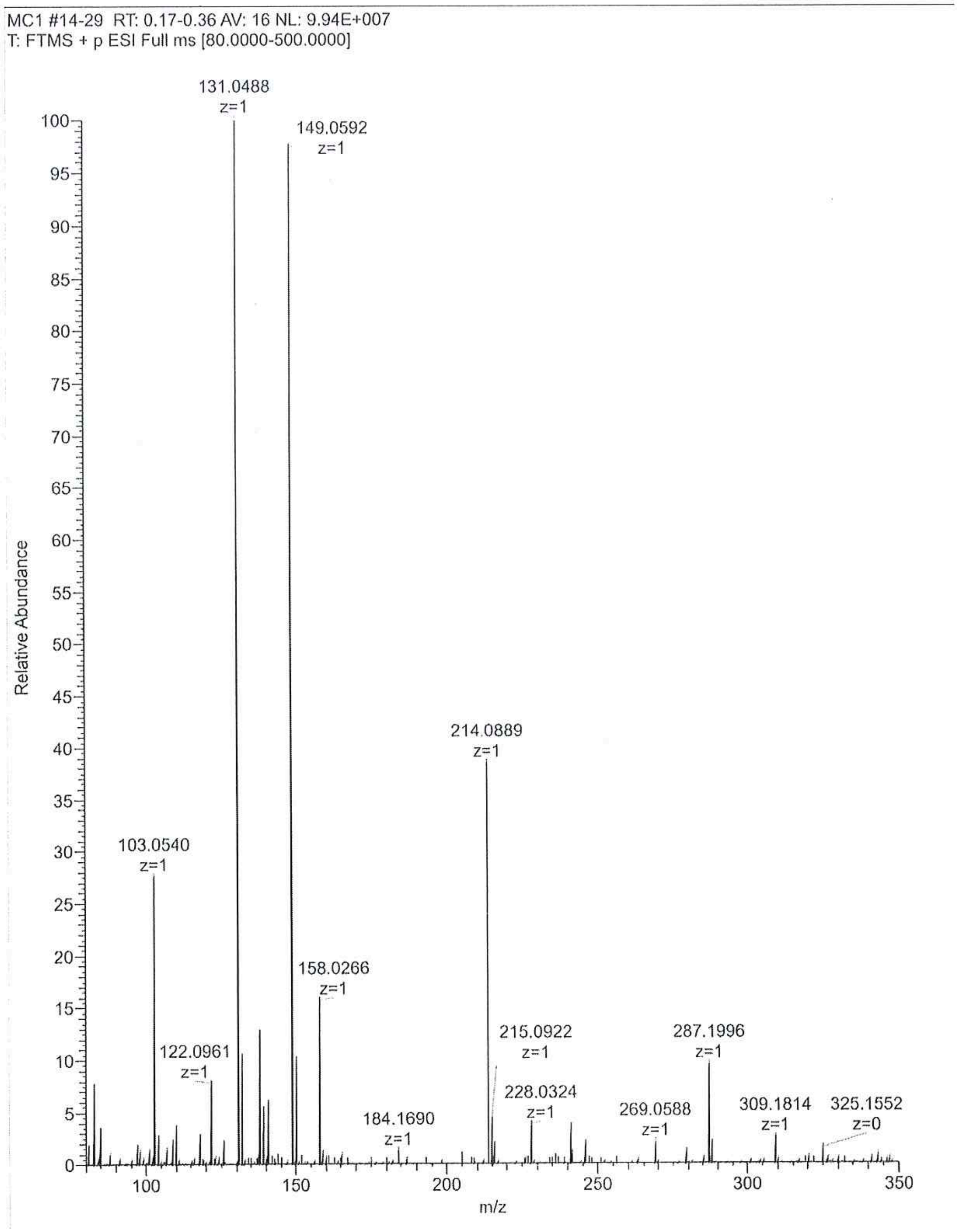

MC2

Agilent Technologies

Sample Name:
MC2
Data Collected on:
m300-mercury300
Archive directory:

Sample directory:

FidFile: PROTON

Pulse Sequence: PROTON (s2pul)
Solvent: cdcl3
Data collected on: Sep 13 2022

Operator: caccia

Relax. delay 1.000 sec
Pulse 45.0 degrees
Acq. time 1.706 sec
Width 4803.1 Hz
64 repetitions
OBSERVE H1, 300.1976543 MHz
DATA PROCESSING
FT size 16384
Total time 2 min 58 sec

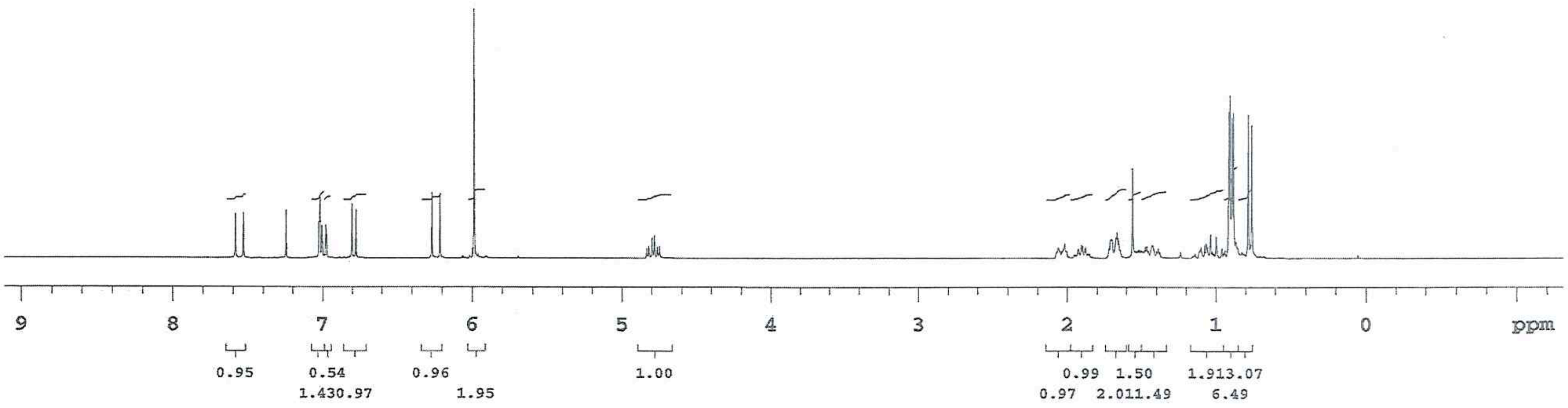

HC2

Gradient Shimming

Sample Name:
MC2_FR_12-53
Data Collected on:
m300-mercury300
Archive directory:
/export/home/chempack/vnmrsys/data
Sample directory:

FidFile: CARBON

Pulse Sequence: CARBON (s2pul)
Solvent: cdcl3
Data collected on: Mar 19 2021

Temp. 17.7 C / 290.9 K
Operator: caccia

Relax. delay 1.000 sec
Pulse 45.0 degrees
Acq. time 0.868 sec
Width 18867.9 Hz
448 repetitions
OBSERVE C13, 75.4847602 MHz
DECOUPLE H1, 300.1991980 MHz
Power 38 dB
continuously on
WALTZ-16 modulated
DATA PROCESSING
Line broadening 0.5 Hz
FT size 32768
Total time 16 min

Agilent Technologies

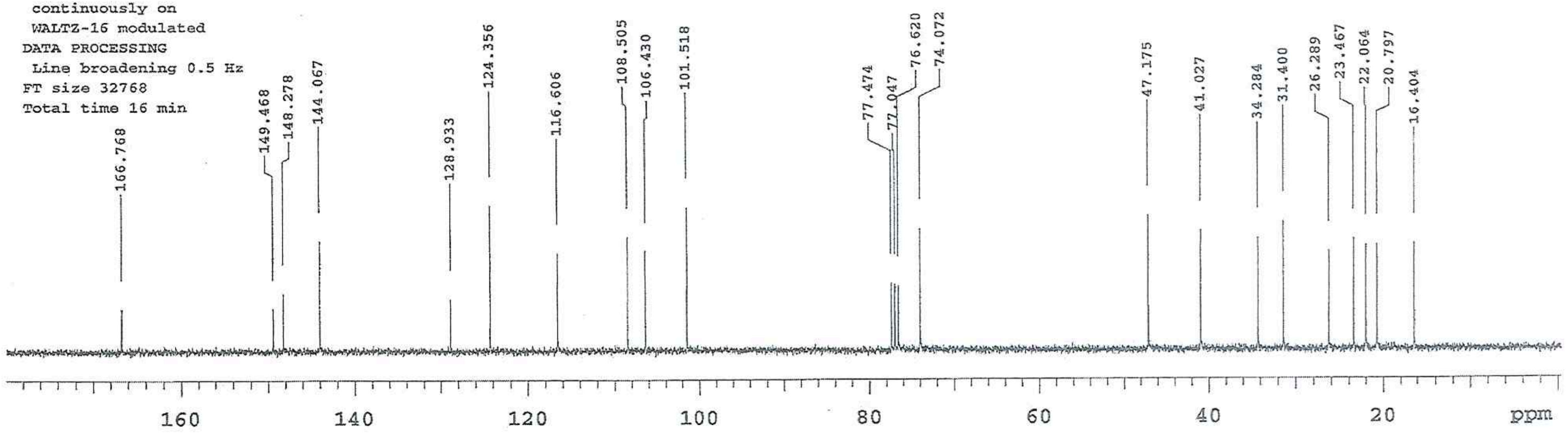

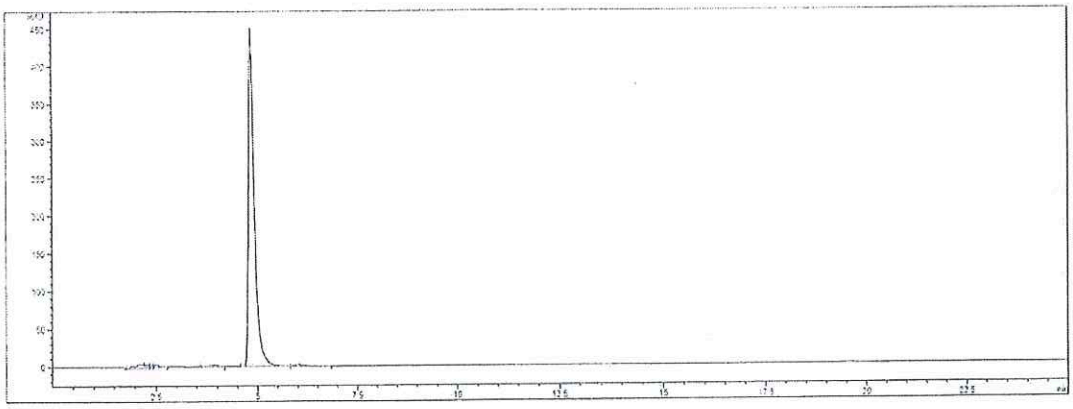

Compound	Retention time	Area%
MC2	4.84	>95

MC2 (330.18)

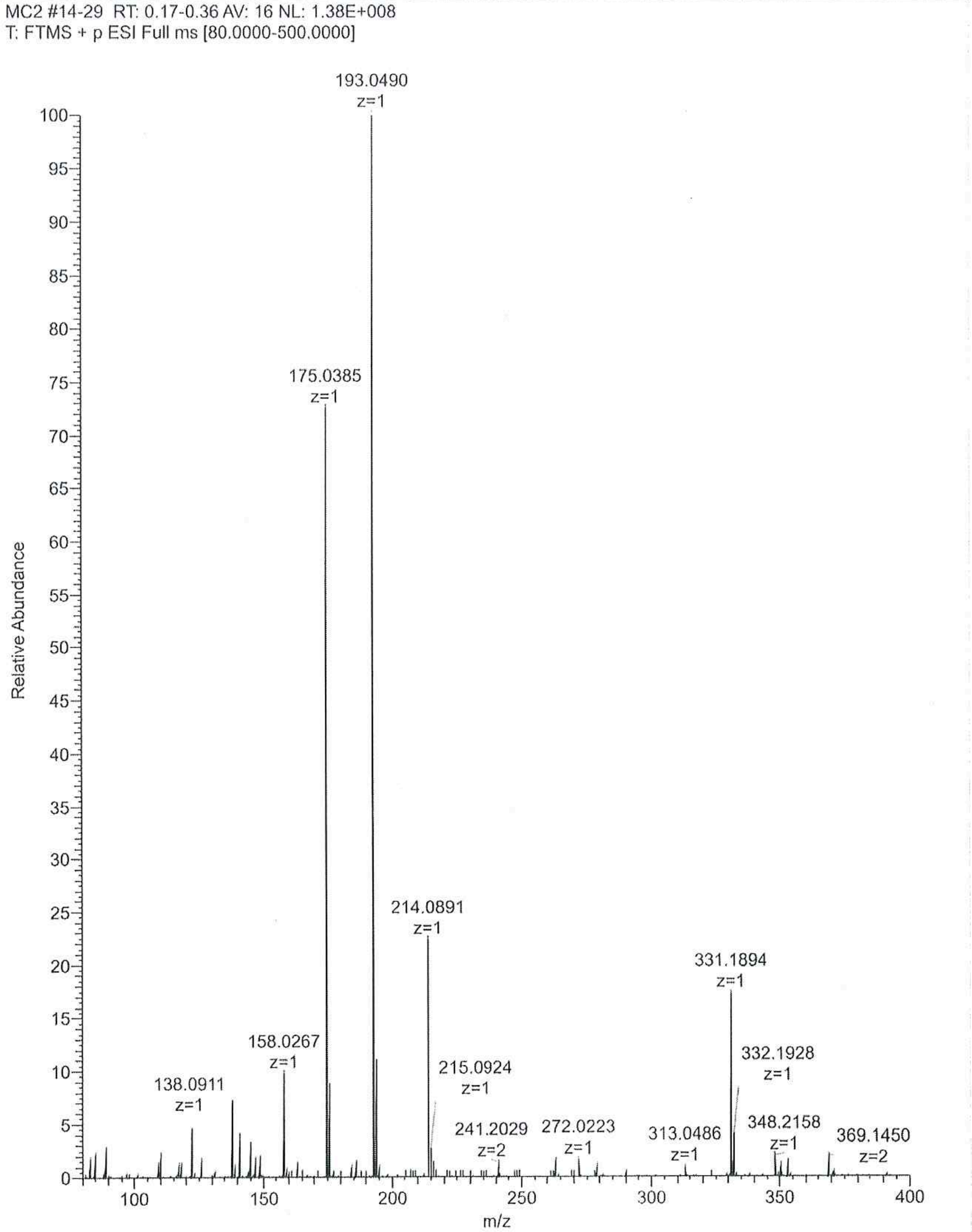

MC2 #14-29 RT: 0.17-0.36 AV: 16 NL: 1.38E+008
T: FTMS + p ESI Full ms [80.0000-500.0000]
193.0490
z=1
175.0385
z=1
214.0891
z=1
331.1894
z=1
158.0267
z=1
332.1928
z=1
215.0924
z=1
138.0911
z=1
241.2029
z=2
272.0223
z=1
313.0486
z=1
348.2158
z=1
369.1450
z=2
Relative Abundance
m/z

MC3

Agilent Technologies

Sample Name:
MC3
Data Collected on:
m300-mercury300
Archive directory:

Sample directory:

FidFile: PROTON

Pulse Sequence: PROTON (s2pul)
Solvent: cdcl3
Data collected on: Sep 13 2022

Operator: caccia

Relax. delay 1.000 sec
Pulse 45.0 degrees
Acq. time 1.706 sec
Width 4803.1 Hz
64 repetitions
OBSERVE H1, 300.1976543 MHz
DATA PROCESSING
FT size 16384
Total time 2 min 58 sec

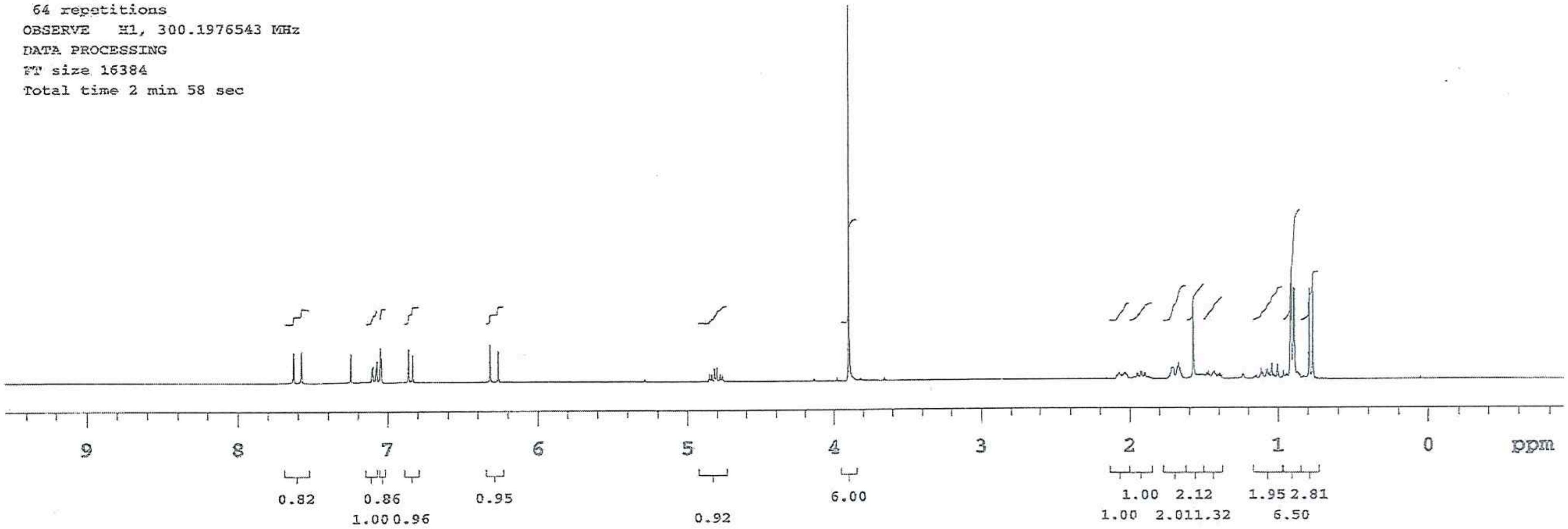

MC3

Gradient Shimming

Sample Name:
mc3
Data Collected on:
m300-mercury300
Archive directory:
/export/home/chempack/vnmrsys/data
Sample directory:

FidFile: CARBON

Pulse Sequence: CARBON (s2pul)
Solvent: cdcl3
Data collected on: Mar 23 2021

Operator: caccia

Relax. delay 1.000 sec
Pulse 45.0 degrees
Acq. time 0.868 sec
Width 18867.9 Hz
2000 repetitions
OBSERVE C13, 75.4847602 MHz
DECOUPLE H1, 300.1991980 MHz
Power 38 dB
continuously on
WALTZ-16 modulated
DATA PROCESSING
Line broadening 0.5 Hz
FT size 32768
Total time 1 hr, 4 min

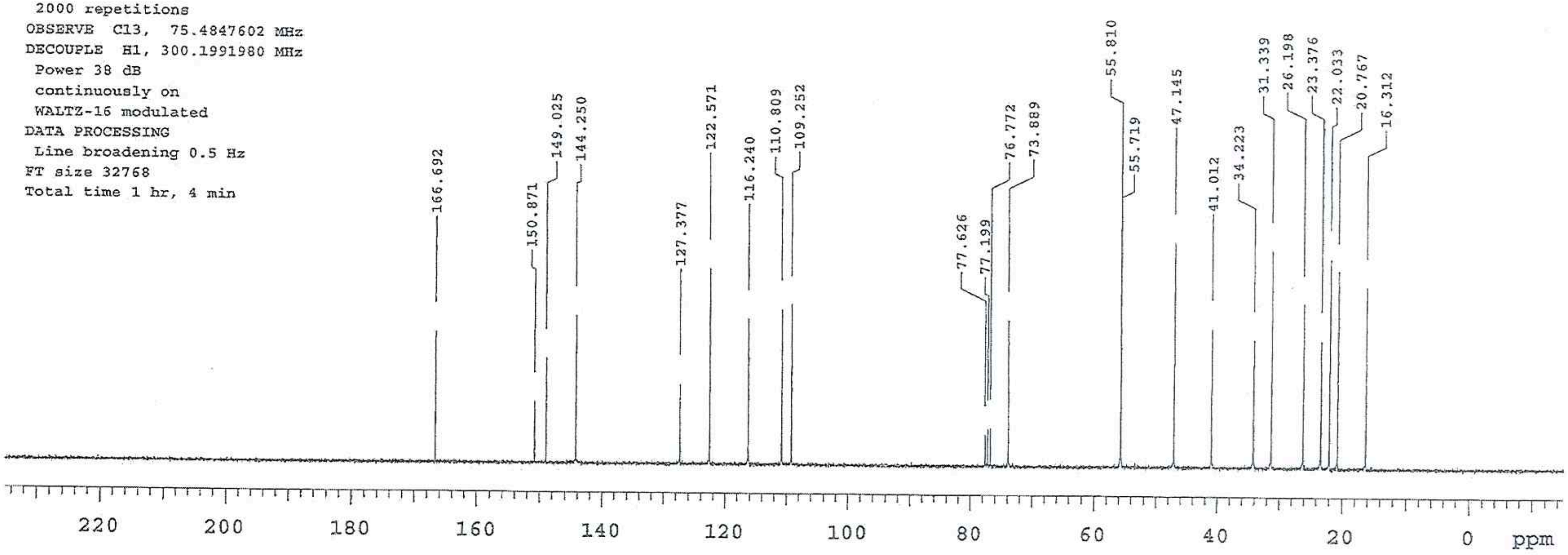

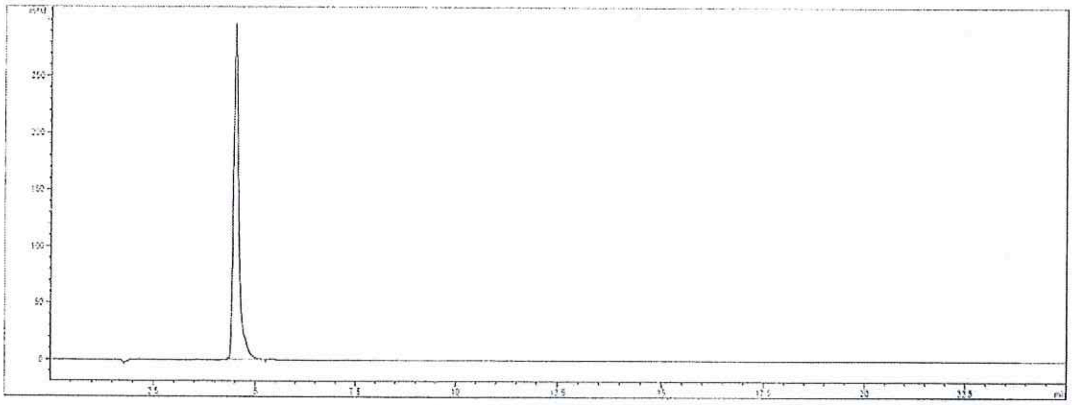

Compound	Retention time	Area%
MC3	4.49	>99

MC3 (346.21)

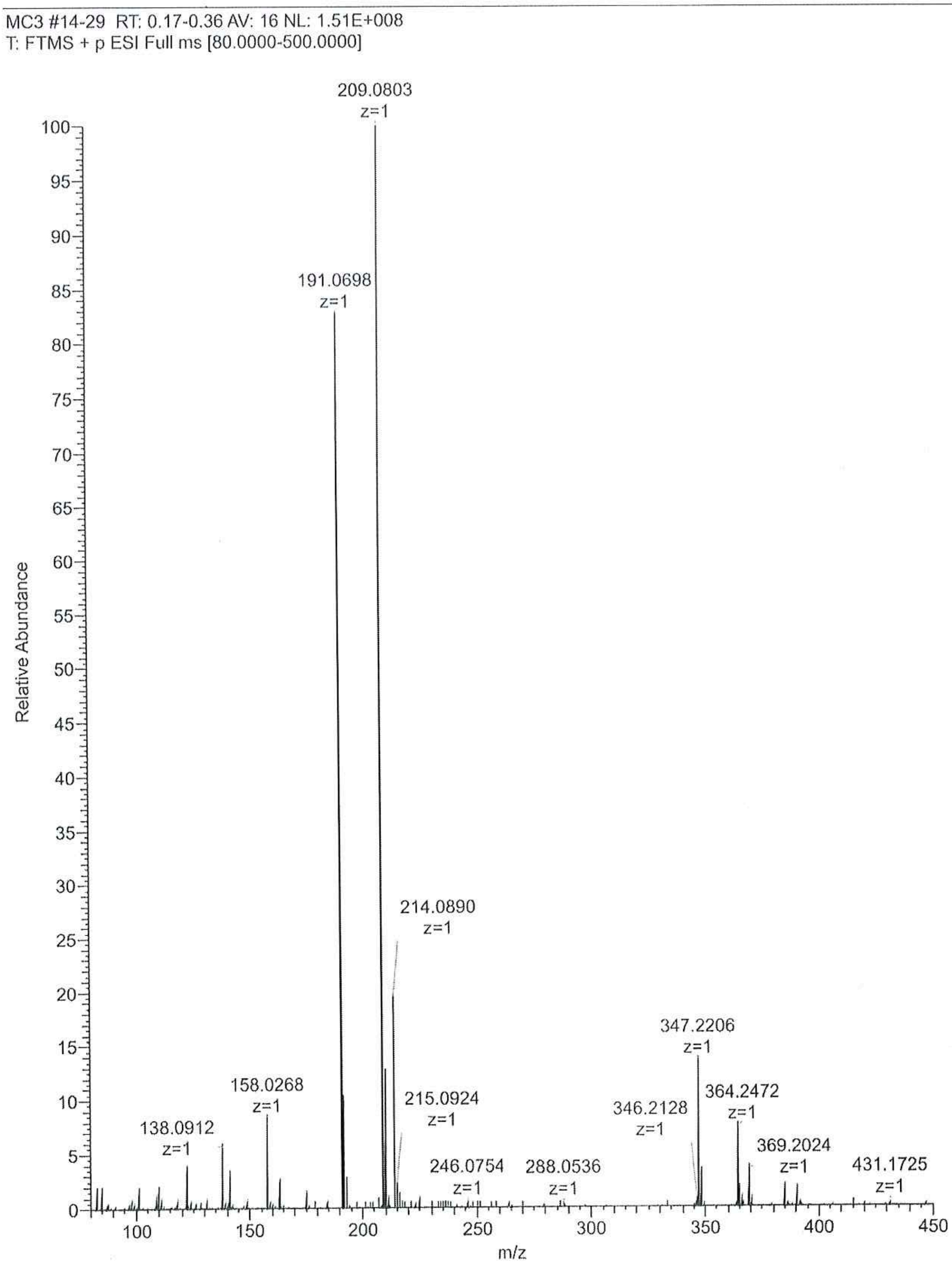
MC3 #14-29 RT: 0.17-0.36 AV: 16 NL: 1.51E+008
T: FTMS + p ESI Full ms [80.0000-500.0000]
209.0803 z=1
191.0698 z=1
214.0890 z=1
215.0924 z=1
158.0268 z=1
138.0912 z=1
246.0754 z=1
288.0536 z=1
347.2206 z=1
346.2128 z=1
364.2472 z=1
369.2024 z=1
431.1725 z=1
Relative Abundance
m/z

MC4

Gradient Shimming

Sample Name:
MC4
Data Collected on:
m300-mercury300
Archive directory:
/export/home/chempack/vnmrsys/data
Sample directory:

FidFile: PROTON

Pulse Sequence: PROTON (s2pul)
Solvent: cdcl3
Data collected on: Mar 11 2022

Operator: caccia

Relax. delay 1.000 sec
Pulse 45.0 degrees
Acq. time 1.706 sec
Width 4803.1 Hz
32 repetitions
OBSERVE H1, 300.1976543 MHz
DATA PROCESSING
FT size 16384
Total time 1 min 29 sec

Agilent Technologies

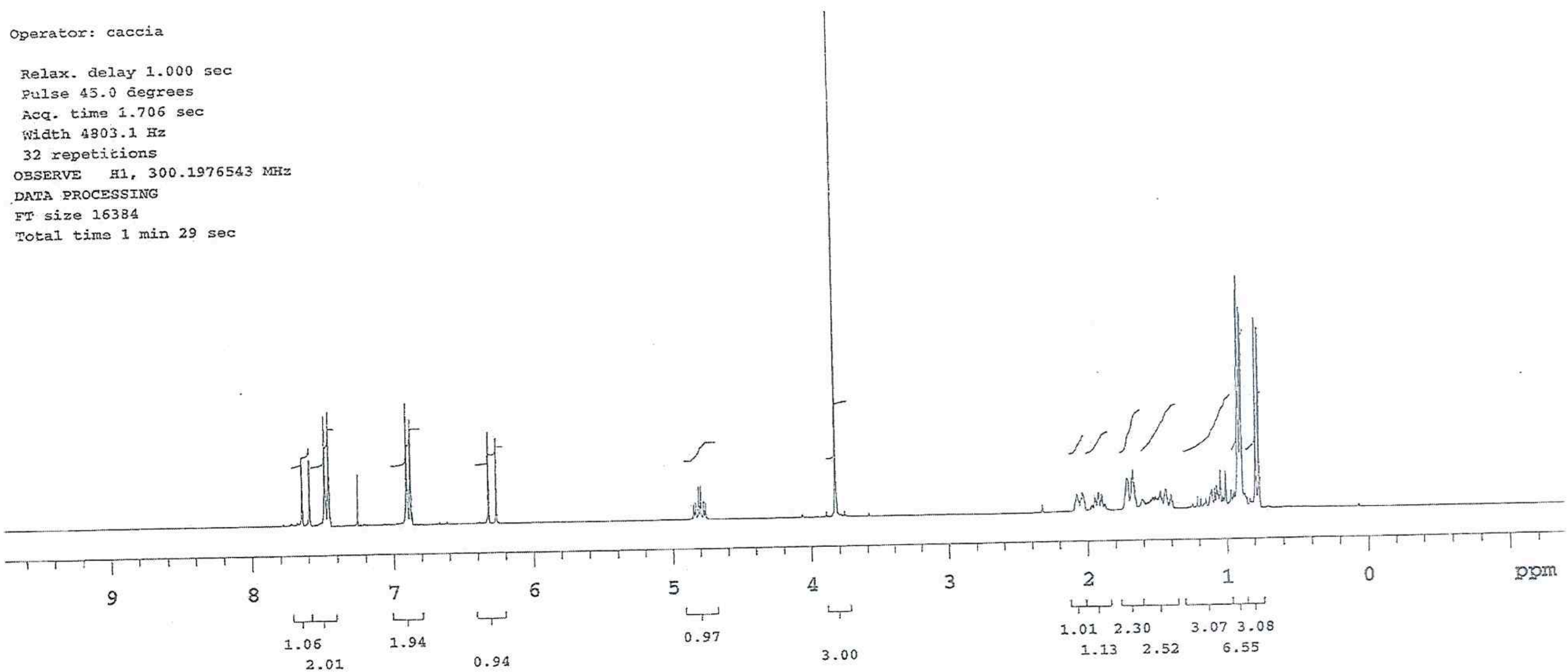

MC4

Gradient Shimming

Sample Name:
MC4_19-79
Data Collected on:
m300-mercury300
Archive directory:
/export/home/chempack/vnmrsys/data
Sample directory:

FidFile: CARBON

Pulse Sequence: CARBON (s2pul)
Solvent: cdcl3
Data collected on: Mar 26 2021

Operator: caccia

Relax. delay 1.000 sec
Pulse 45.0 degrees
Acq. time 0.868 sec
Width 18867.9 Hz
128 repetitions
OBSERVE C13, 75.4847602 MHz
DECOUPLE H1, 300.1991980 MHz
Power 38 dB
continuously on
WALTZ-16 modulated
DATA PROCESSING
Line broadening 0.5 Hz
FT size 32768
Total time 8 min 15 sec

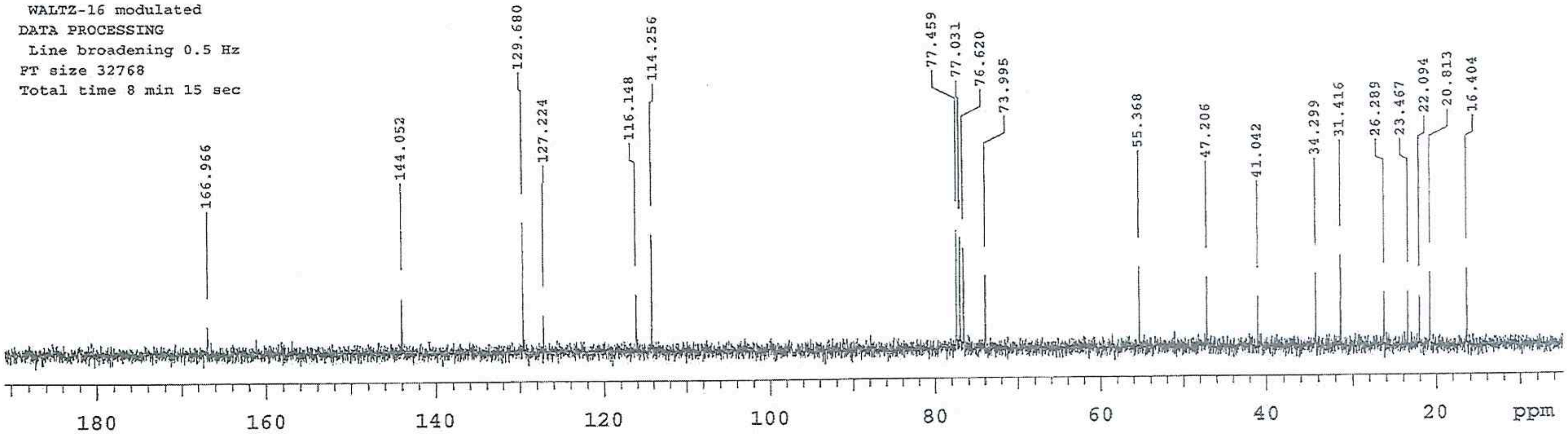

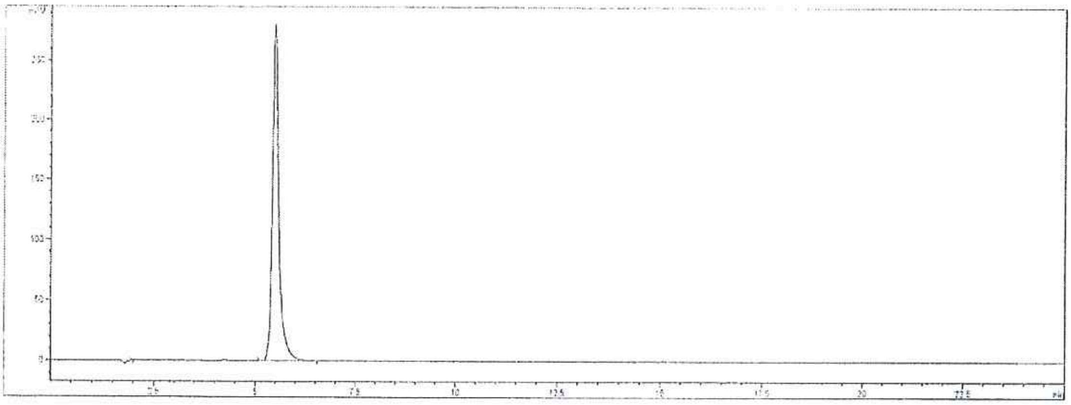

Compound	Retention time	Area%
MC4	5.48	>99

MC4 (316.20)

MC4 #13-29 RT: 0.17-0.36 AV: 17 NL: 1.85E+008
T: FTMS + p ESI Full ms [80.0000-500.0000]

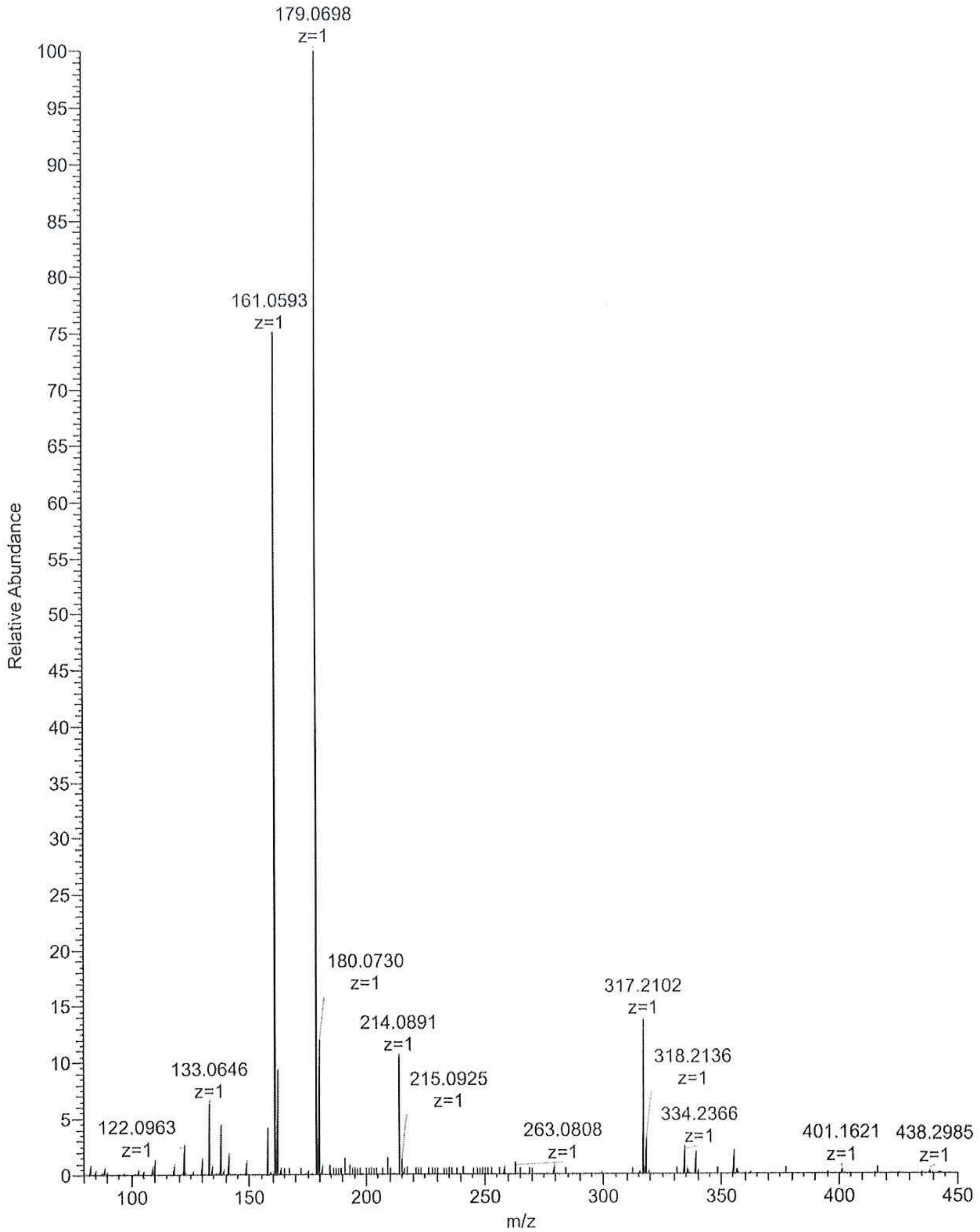

MC5

Gradient Shimming

Sample Name:
MC5
Data Collected on:
m300-mercury300
Archive directory:
/export/home/chempack/vnmrsys/data
Sample directory:

FidFile: PROTON

Pulse Sequence: PROTON (s2pul)
Solvent: cdcl3
Data collected on: Mar 11 2022

Operator: caccia

Relax. delay 1.000 sec
Pulse 45.0 degrees
Acq. time 1.706 sec
Width 4803.1 Hz
32 repetitions
OBSERVE H1, 300.1976543 MHz
DATA PROCESSING
FT size 16384
Total time 1 min 29 sec

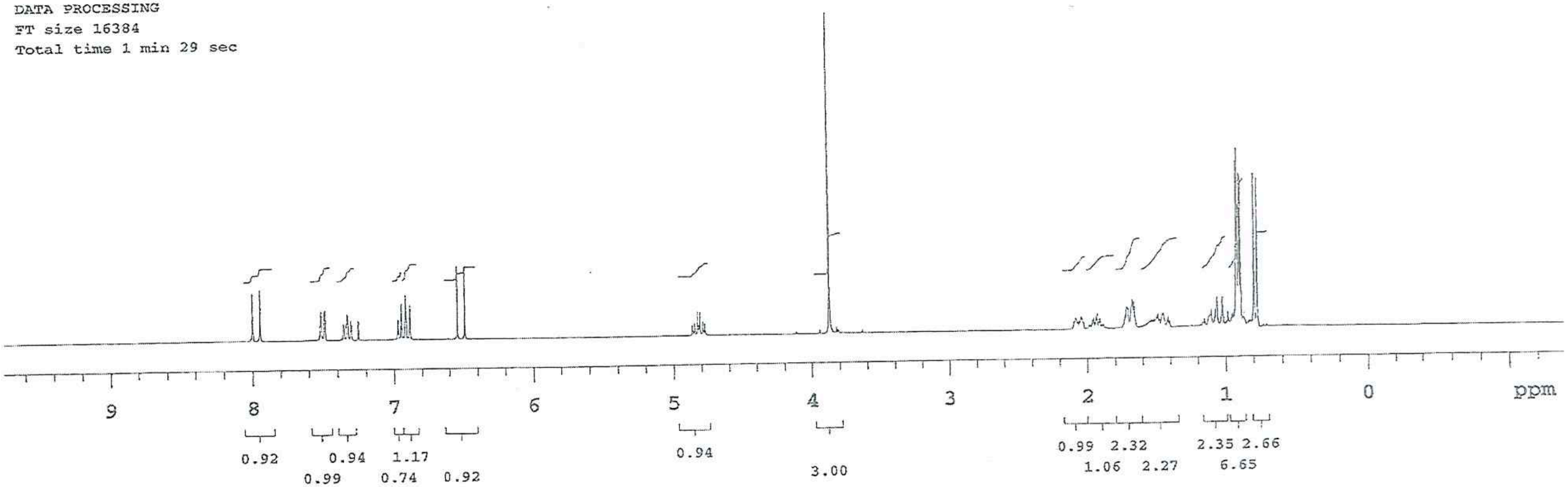

MC5

Gradient Shimming

Sample Name:
MC5_1-21
Data Collected on:
m300-mercury300
Archive directory:
/export/home/chempack/vnmrsys/data
Sample directory:

FidFile: CARBON

Pulse Sequence: CARBON (s2pul)
Solvent: cdcl3
Data collected on: Mar 31 2021

Operator: caccia

Relax. delay 1.000 sec
Pulse 45.0 degrees
Acq. time 0.868 sec
Width 18867.9 Hz
1000 repetitions
OBSERVE C13, 75.4847602 MHz
DECOUPLE H1, 300.1991980 MHz
Power 38 dB
continuously on
WALTZ-16 modulated
DATA PROCESSING
Line broadening 0.5 Hz
FT size 32768
Total time 32 min

Agilent Technologies

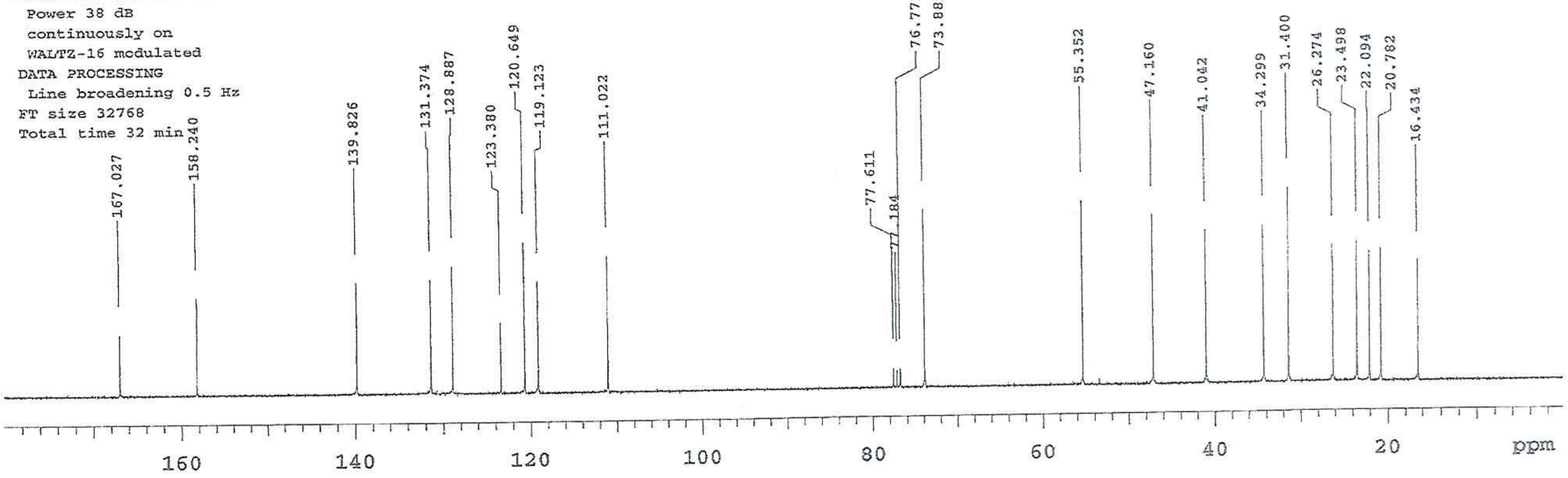

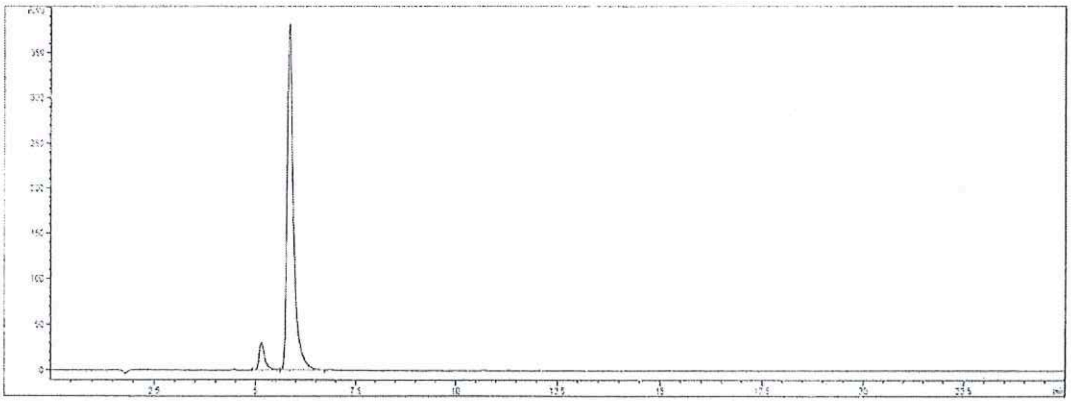

Compound	Retention time	Area%
MC5	5.84	>94

MC5 (316.20)

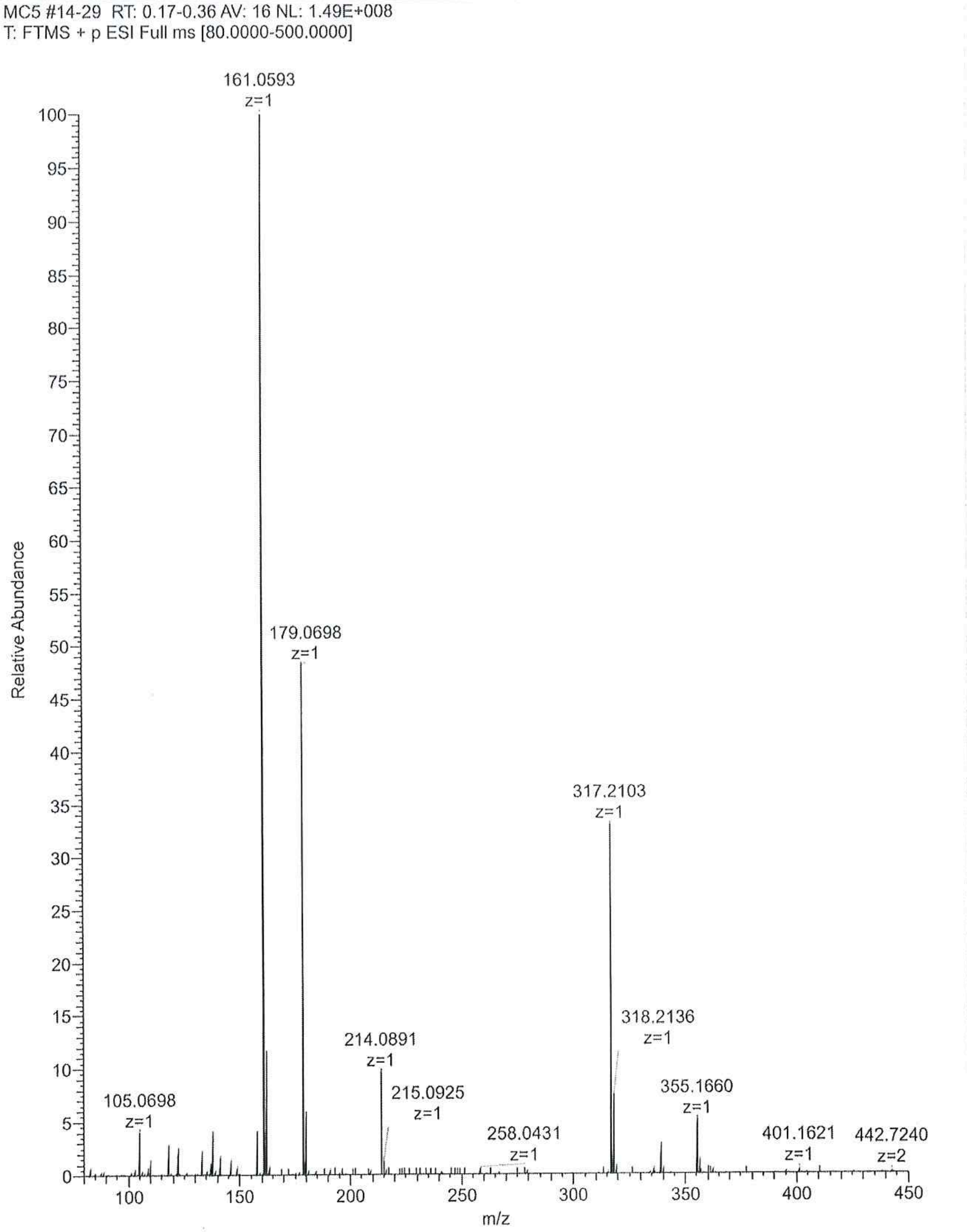

MC5 #14-29 RT: 0.17-0.36 AV: 16 NL: 1.49E+008
T: FTMS + p ESI Full ms [80.0000-500.0000]
161.0593
z=1
179.0698
z=1
317.2103
z=1
318.2136
z=1
214.0891
z=1
215.0925
z=1
355.1660
z=1
105.0698
z=1
258.0431
z=1
401.1621
z=1
442.7240
z=2
Relative Abundance
m/z

MC6

Gradient Shimming

Sample Name:
MC6
Data Collected on:
m300-mercury300
Archive directory:
/export/home/chempack/vnmrsys/data
Sample directory:

FidFile: PROTON

Pulse Sequence: PROTON (s2pul)
Solvent: cdcl3
Data collected on: Mar 11 2022

Operator: caccia

Relax. delay 1.000 sec
Pulse 45.0 degrees
Acq. time 1.706 sec
Width 4803.1 Hz
32 repetitions
OBSERVE H1, 300.1976543 MHz
DATA PROCESSING
FT size 16384
Total time 1 min 29 sec

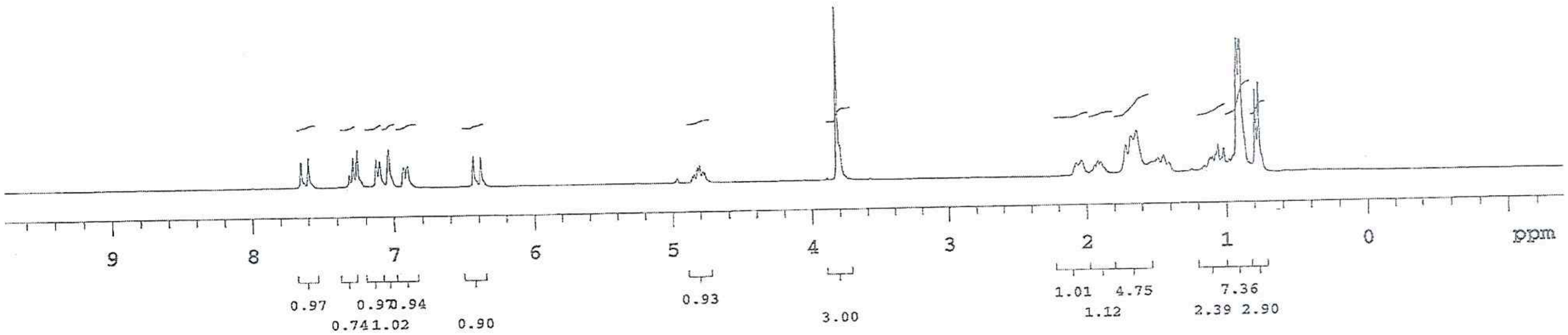

MC6

Gradient Shimming

Agilent Technologies

Sample Name:
MC5
Data collected on:
m300-mercury300
Archive directory:
/export/home/chempack/vnmrsys/data
Sample directory:

FidFile: CARBON

Pulse Sequence: CARBON (s2pul)
Solvent: cdcl3
Data collected on: Apr 9 2021

Operator: caccia

Relax. delay 1.000 sec
Pulse 45.0 degrees
Acq. time 0.868 sec
Width 18867.9 Hz
2000 repetitions
OBSERVE C13, 75.4847602 MHz
DECOUPLE H1, 300.1991980 MHz
Power 38 dB
continuously on
WALTZ-16 modulated
DATA PROCESSING
Line broadening 0.5 Hz
FT size 32768
Total time 1 hr, 4 min

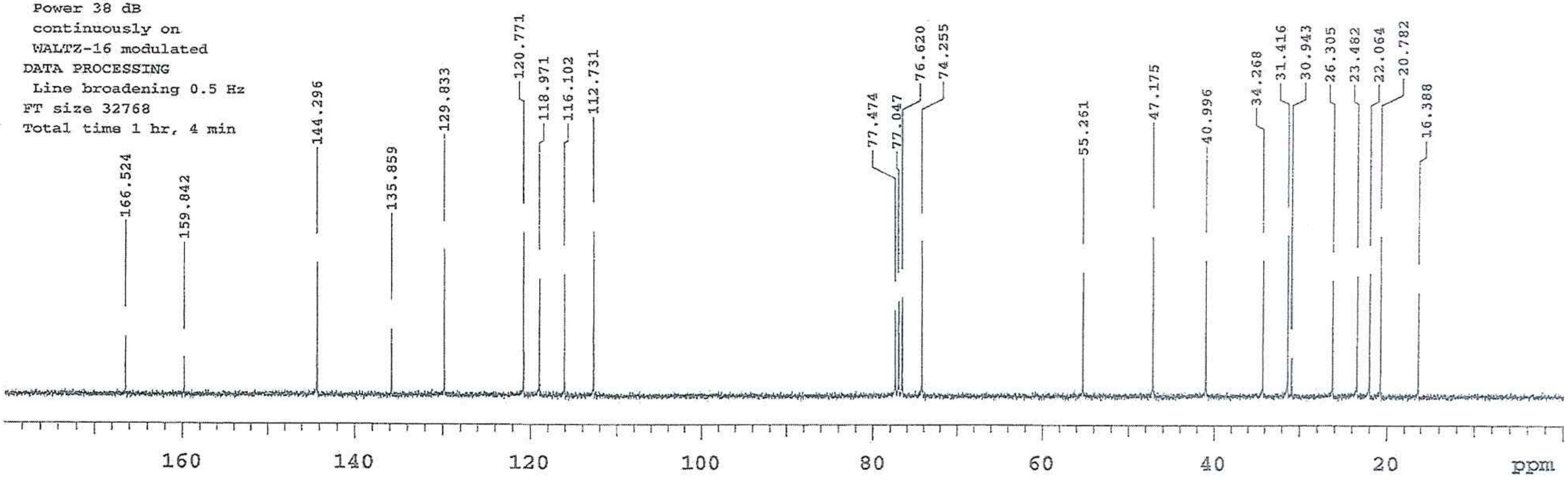

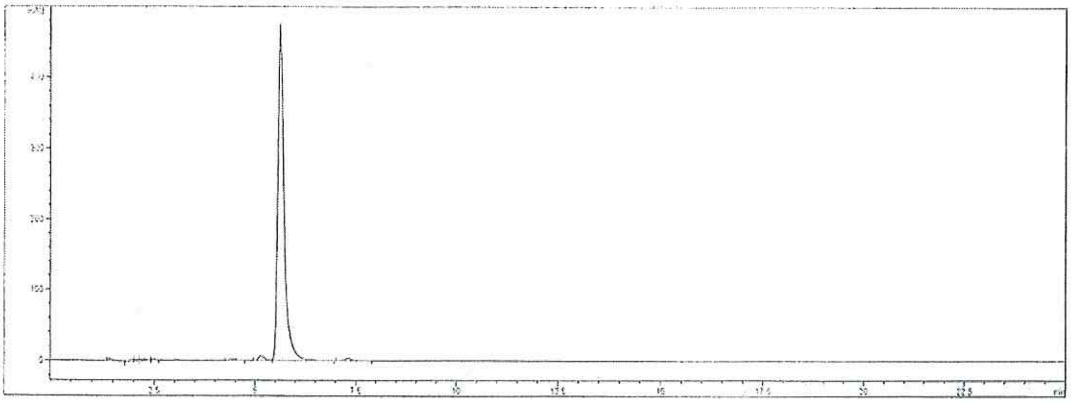

Compound	Retention time	Area%
MC6	5.61	>95

MC6 (316.20)

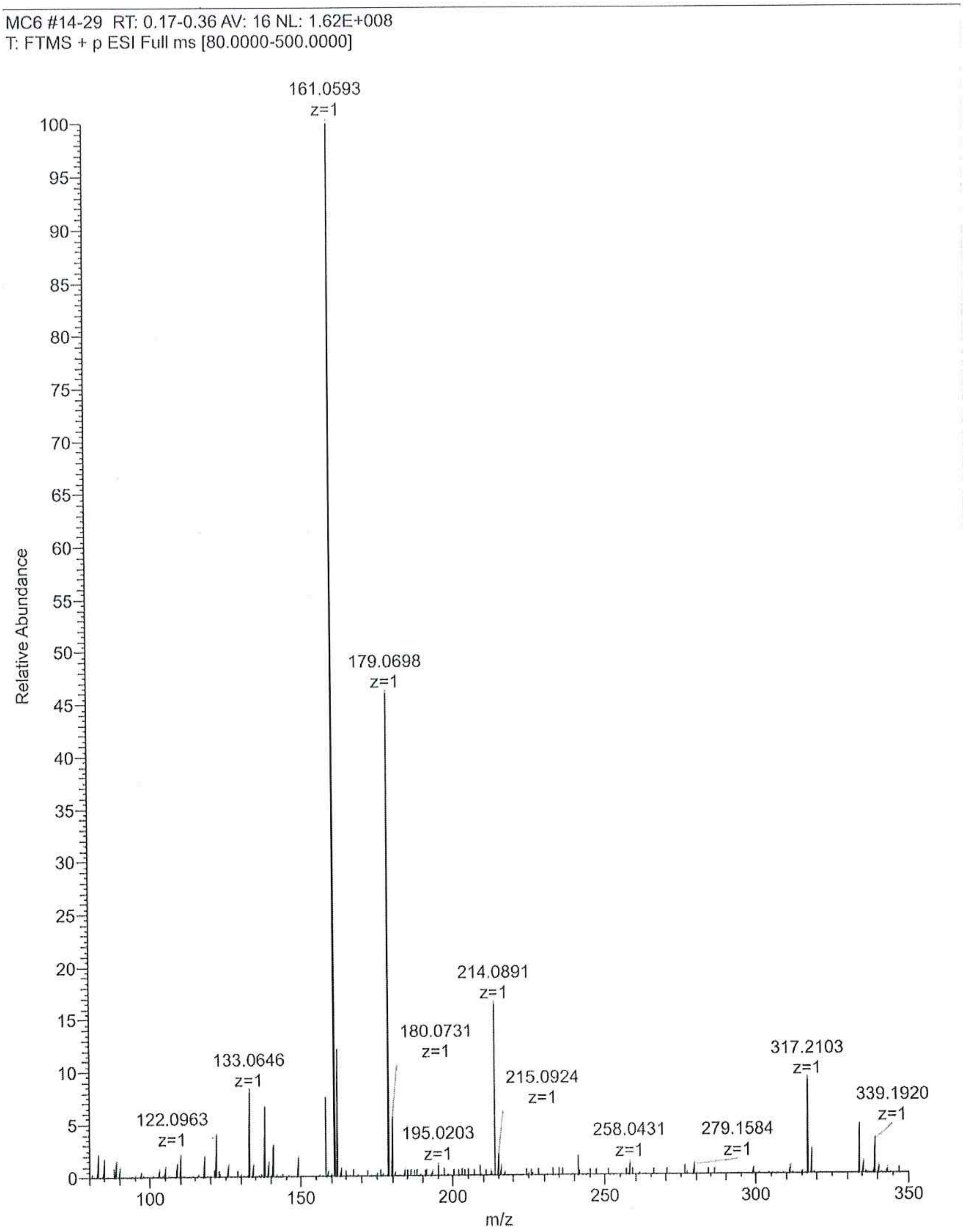
MC6 #14-29 RT: 0.17-0.36 AV: 16 NL: 1.62E+008
T: FTMS + p ESI Full ms [80.0000-500.0000]
161.0593
z=1
179.0698
z=1
214.0891
z=1
180.0731
z=1
133.0646
z=1
215.0924
z=1
317.2103
z=1
339.1920
z=1
122.0963
z=1
195.0203
z=1
258.0431
z=1
279.1584
z=1
Relative Abundance
m/z

MC7

Gradient Shimming

Sample Name:
MC7
Data Collected on:
m300-mercury300
Archive directory:
/export/home/chempack/vnmrsys/data
Sample directory:

FidFile: PROTON

Pulse Sequence: PROTON (s2pul)
Solvent: cdcl3
Data collected on: Mar 11 2022

Operator: caccia

Relax. delay 1.000 sec
Pulse 45.0 degrees
Acq. time 1.706 sec
Width 4803.1 Hz
32 repetitions
OBSERVE H1, 300.1976543 MHz
DATA PROCESSING
FT size 16384
Total time 1 min 29 sec

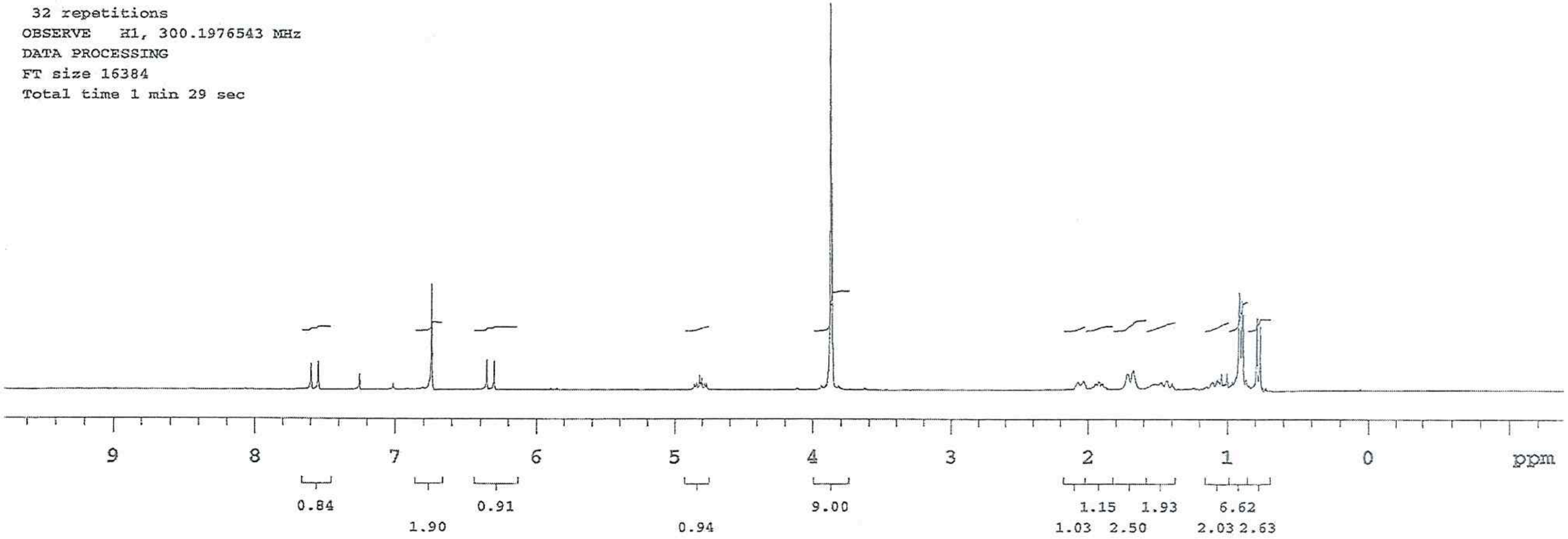

MC7

Agilent Technologies

Gradient Shimming

Sample Name:
MC7
Data Collected on:
m300-mercury300
Archive directory:
/export/home/chempack/vnmrsys/data
Sample directory:

FidFile: CARBON

Pulse Sequence: CARBON (s2pul)
Solvent: cdcl3
Data collected on: Apr 27 2021

Operator: caccia

Relax. delay 1.000 sec
Pulse 45.0 degrees
Acq. time 0.868 sec
Width 18867.9 Hz
2000 repetitions
OBSERVE C13, 75.4847602 MHz
DECOUPLE H1, 300.1991980 MHz
Power 38 dB
continuously on
WALTZ-15 modulated
DATA PROCESSING
Line broadening 0.5 Hz
FT size 32768
Total time 1 hr, 4 min

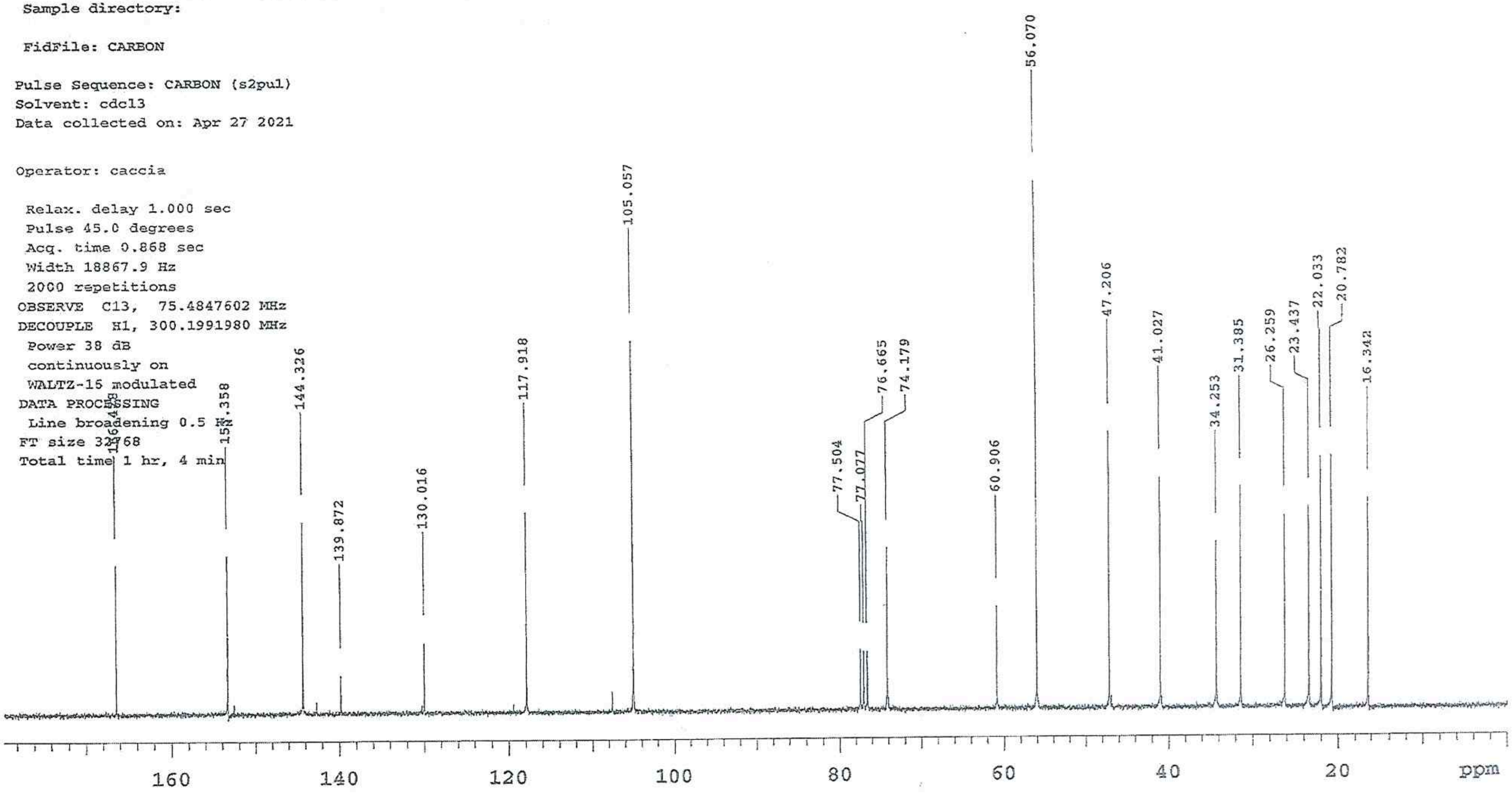

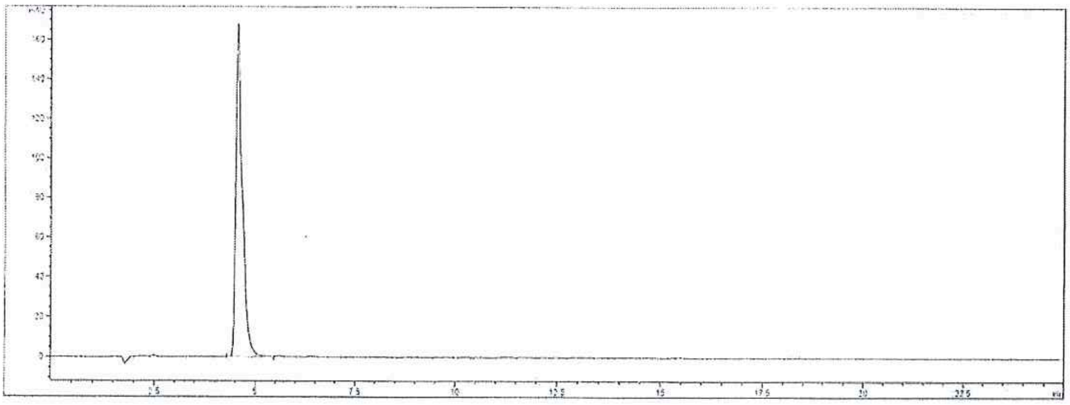

Compound	Retention time	Area%
MC7	4.55	>99

MC7 (376.22)

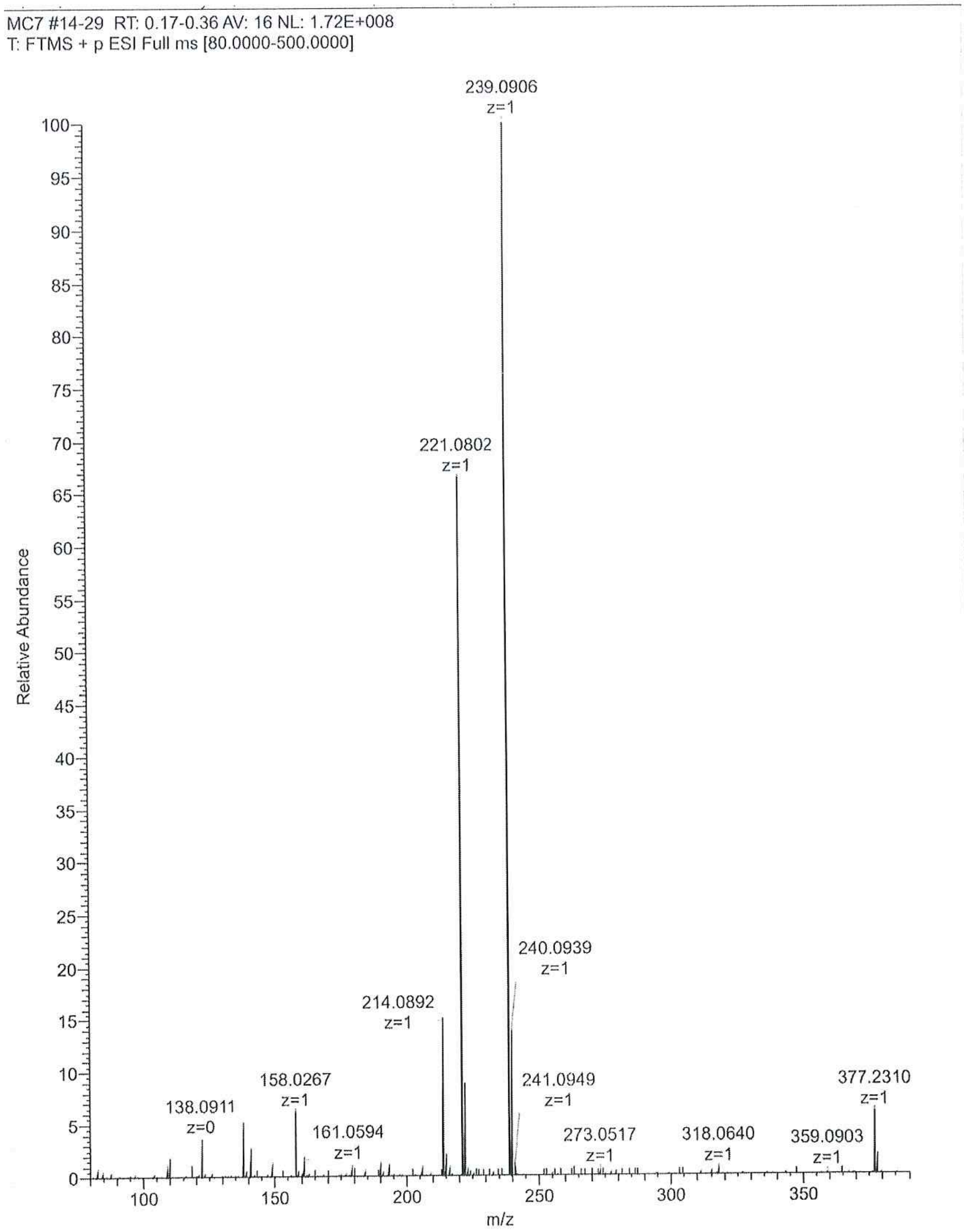
MC7 #14-29 RT: 0.17-0.36 AV: 16 NL: 1.72E+008
T: FTMS + p ESI Full ms [80.0000-500.0000]
239.0906 z=1
221.0802 z=1
240.0939 z=1
214.0892 z=1
158.0267 z=1
241.0949 z=1
377.2310 z=1
138.0911 z=0
161.0594 z=1
273.0517 z=1
318.0640 z=1
359.0903 z=1
Relative Abundance
m/z

MF1

Gradient Shimming

Sample Name:
MF1-69-113
Data Collected on:
m300-mercury300
Archive directory:
/export/home/chempack/vnmrsys/data
Sample directory:

FidFile: PROTON

Pulse Sequence: PROTON (s2pul)
Solvent: cdcl3
Data collected on: Jun 24 2021

Operator: caccia

Relax. delay 1.000 sec
Pulse 45.0 degrees
Acq. time 1.706 sec
Width 4803.1 Hz
64 repetitions
OBSERVE H1, 300.1976543 MHz
DATA PROCESSING
FT size 16384
Total time 2 min 58 sec

Agilent Technologies

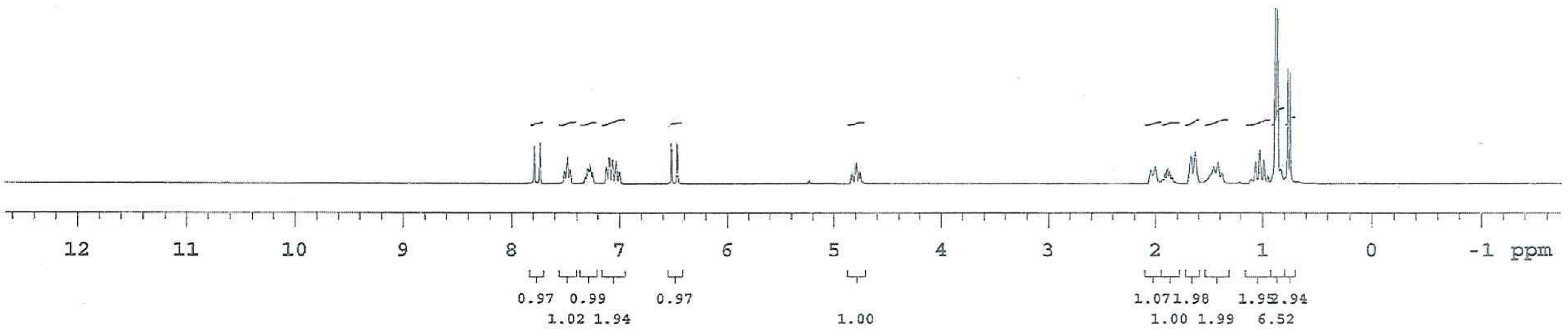

MF1

Agilent Technologies

Gradient Shimming

Sample Name:
MF1_69-113
Data Collected on:
m300-mercury300
Archive directory:
/export/home/chempack/vnmrsys/data
Sample directory:

FidFile: CARBON

Pulse Sequence: CARBON (s2pul)
Solvent: cdcl3
Data collected on: Jun 25 2021

Operator: caccia

Relax. delay 1.000 sec
Pulse 45.0 degrees
Acq. time 0.868 sec
Width 18867.9 Hz
2000 repetitions
OBSERVE C13, 75.4847602 MHz
DECOUPLE H1, 300.1991980 MHz
Power 38 dB
continuously on
WALTZ-16 modulated
DATA PROCESSING
Line broadening 0.5 Hz
FT size 32768
Total time 1 hr, 4 min

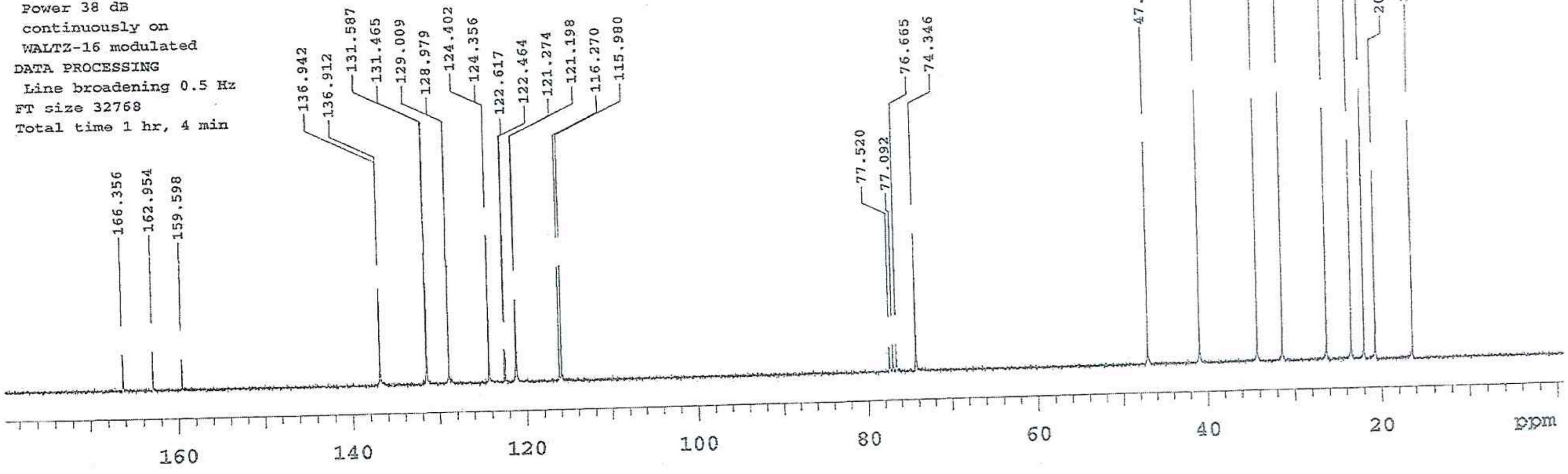

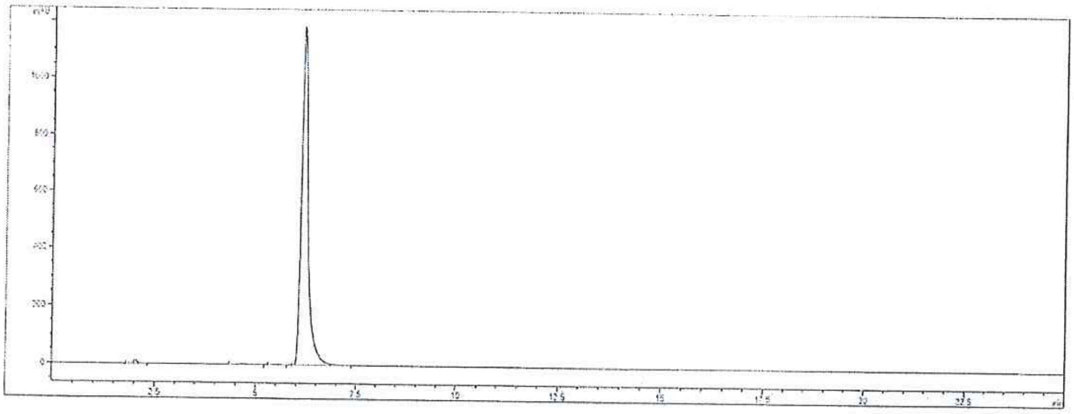

Compound	Retention time	Area%
MF1	6.16	>98

MF2

Gradient Shimming

Sample Name:
MF2_35-55
Data Collected on:
m300-mercury300
Archive directory:
/export/home/chempack/vnmrsys/data
Sample directory:

FidFile: PROTON

Pulse Sequence: PROTON (s2pul)
Solvent: cdcl3
Data collected on: Jun 29 2021

Operator: caccia

Relax. delay 1.000 sec
Pulse 45.0 degrees
Acq. time 1.706 sec
Width 4803.1 Hz
128 repetitions
OBSERVE H1, 300.1976543 MHz
DATA PROCESSING
FT size 16384
Total time 5 min 55 sec

Agilent Technologies

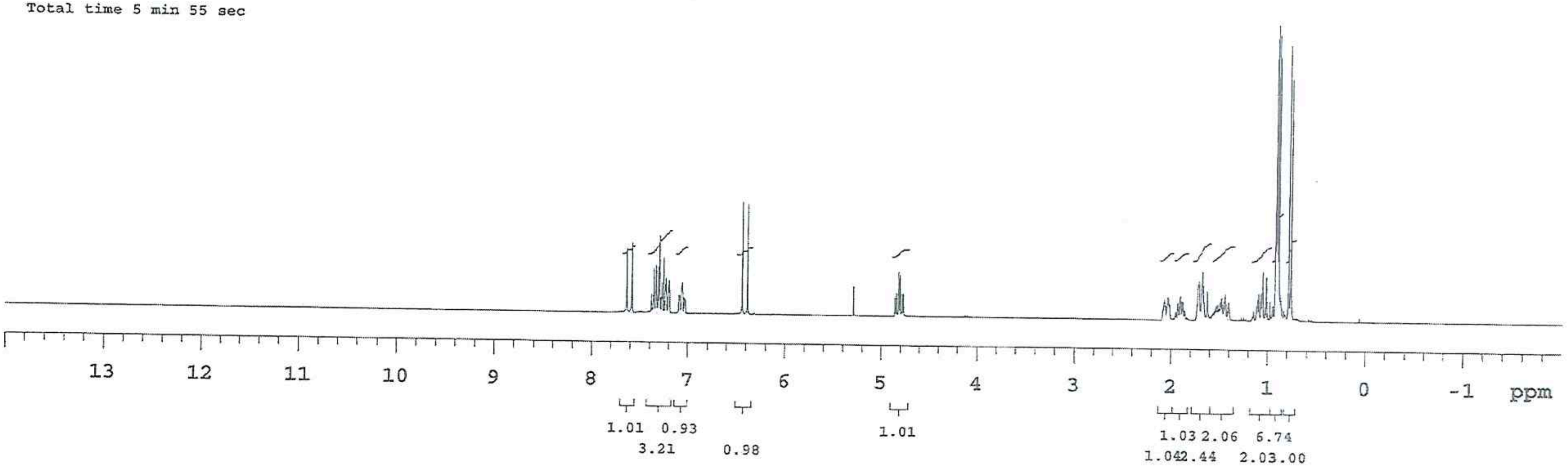

MF2

Gradient Shimming

Sample Name:
MF2
Data Collected on:
m300-mercury300
Archive directory:
/export/home/chempack/vnmrsys/data
Sample directory:

FidFile: CARBON

Pulse Sequence: CARBON (s2pul)
Solvent: cdcl3
Data collected on: Jul 12 2021

Operator: caccia

Relax. delay 1.000 sec
Pulse 45.0 degrees
Acq. time 0.868 sec
Width 18867.9 Hz
2000 repetitions
OBSERVE C13, 75.4847602 MHz
DECOUPLE H1, 300.1991980 MHz
Power 38 dB
continuously on
WALTZ-16 modulated
DATA PROCESSING
Line broadening 0.5 Hz
FT size 32768
Total time 1 hr, 4 min

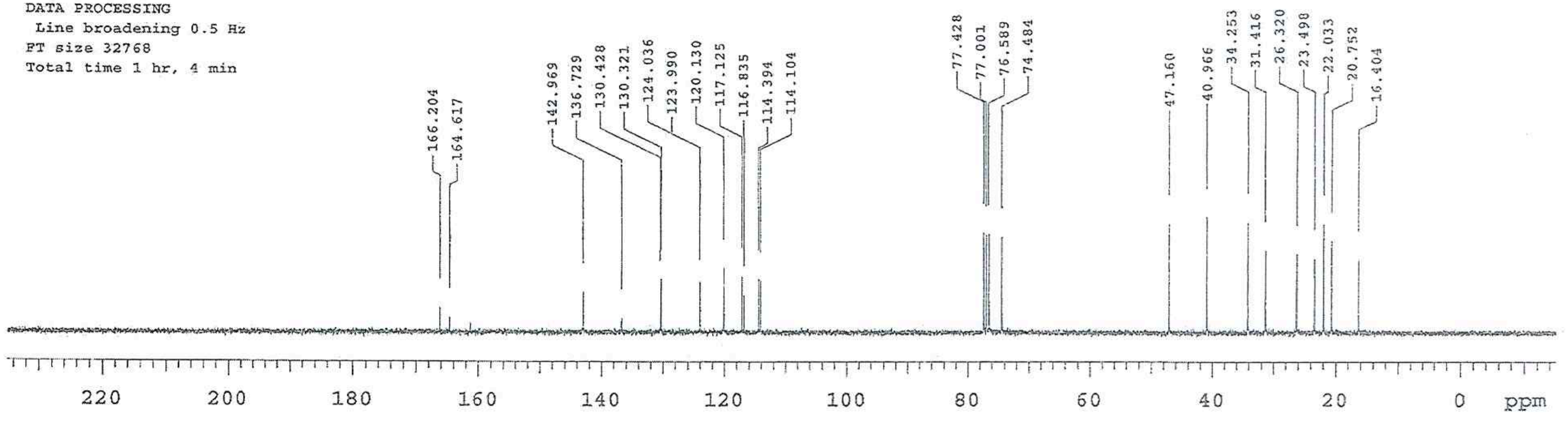

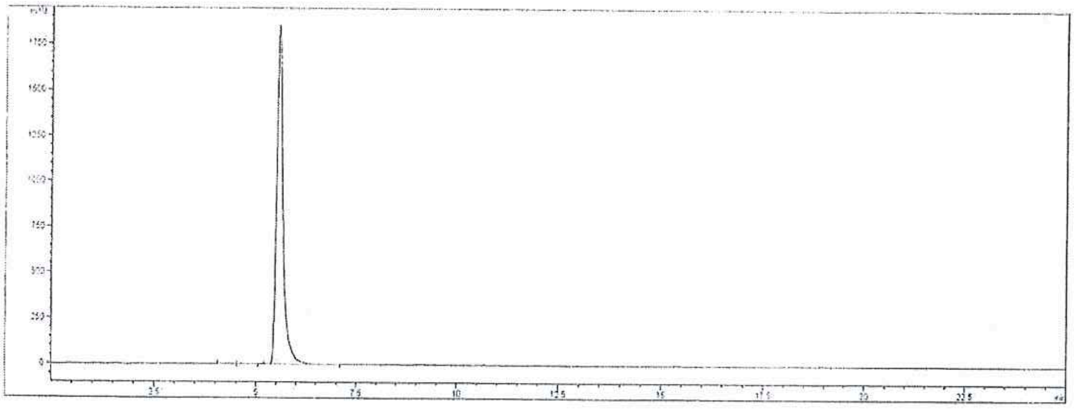

Compound	Retention time	Area%
MF2	5.55	>99

MF3

Gradient Shimming

Sample Name:
MF3_39-71
Data Collected on:
m300-mercury300
Archive directory:
/export/home/chempack/vnmrsys/data
Sample directory:

FidFile: PROTON

Pulse Sequence: PROTON (s2pul)
Solvent: cdcl3
Data collected on: Jul 7 2021

Operator: caccia

Relax. delay 1.000 sec
Pulse 45.0 degrees
Acq. time 1.706 sec
Width 4803.1 Hz
32 repetitions
OBSERVE H1, 300.1976543 MHz
DATA PROCESSING
FT size 16384
Total time 1 min 29 sec

Agilent Technologies

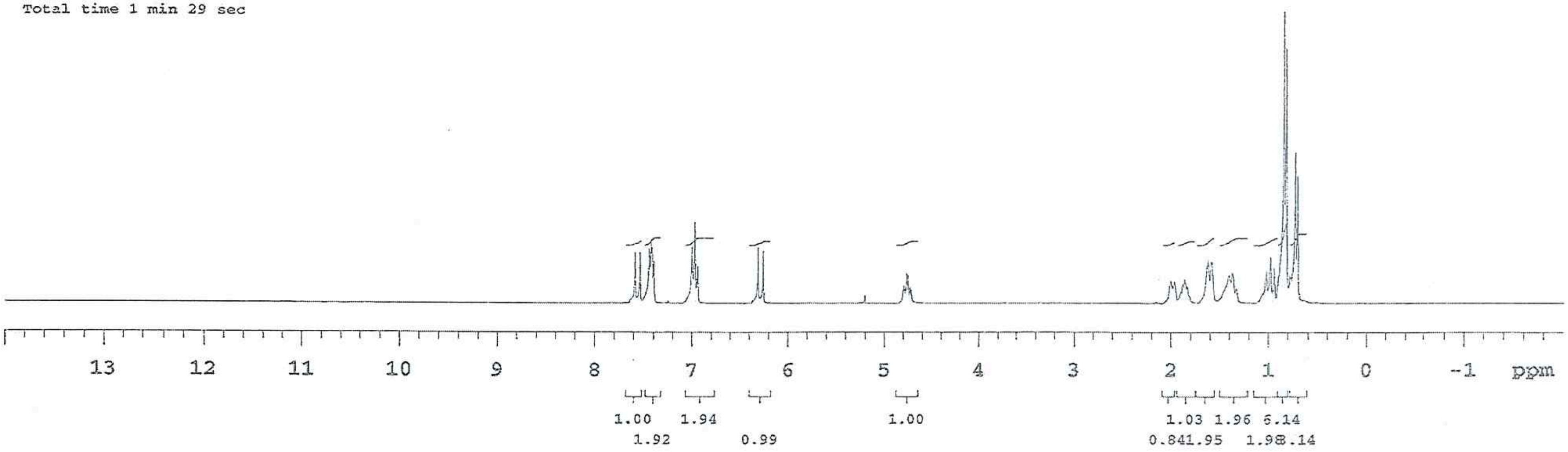

MF3

Gradient Shimming

Sample Name:
MF3_39-71
Data Collected on:
m300-mercury300
Archive directory:
/export/home/chempack/vnmrsys/data
Sample directory:

FidFile: CARBON

Pulse Sequence: CARBON (s2pul)
Solvent: cdcl3
Data collected on: Jul 8 2021

Operator: caccia

Relax. delay 1.000 sec
Pulse 45.0 degrees
Acq. time 0.868 sec
Width 18867.9 Hz
2000 repetitions
OBSERVE C13, 75.4847602 MHz
DECOUPLE H1, 300.1991980 MHz
Power 38 dB
continuously on
WALTZ-16 modulated
DATA PROCESSING
Line broadening 0.5 Hz
FT size 32768
Total time 1 hr,. 4 min

Agilent Technologies

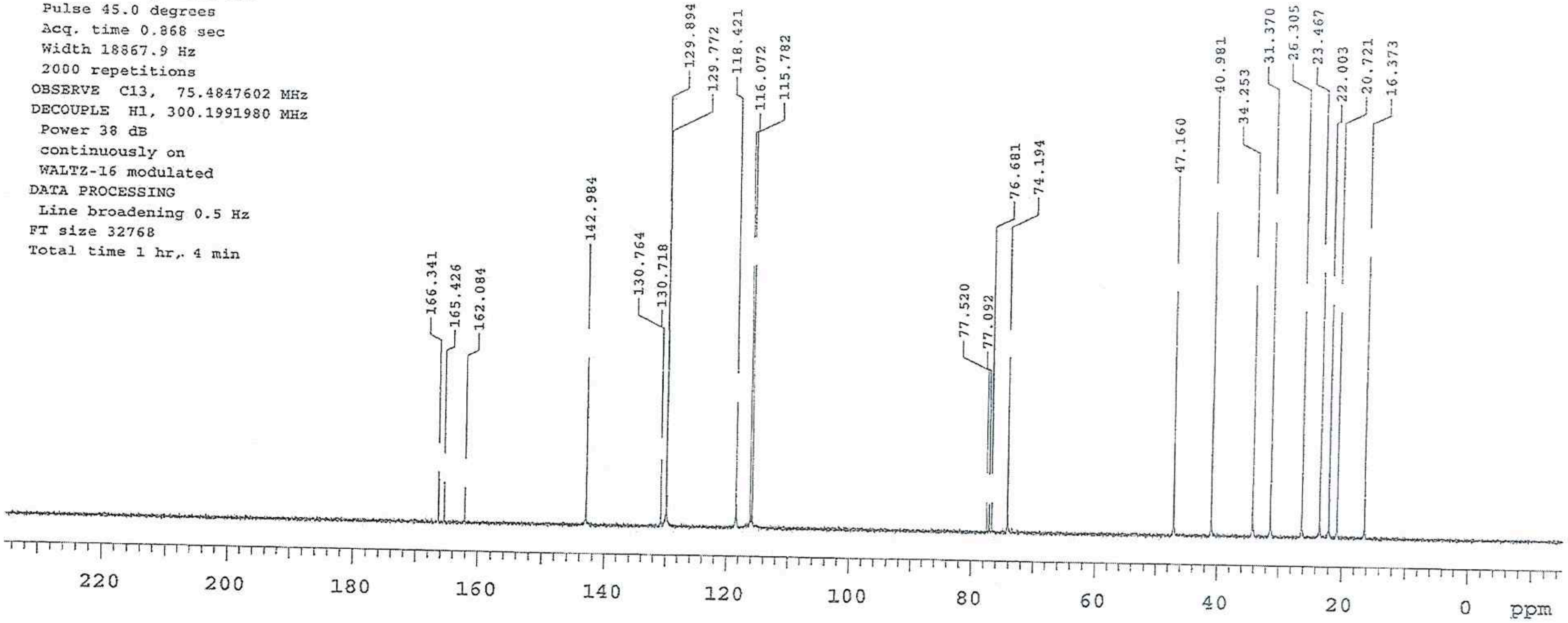

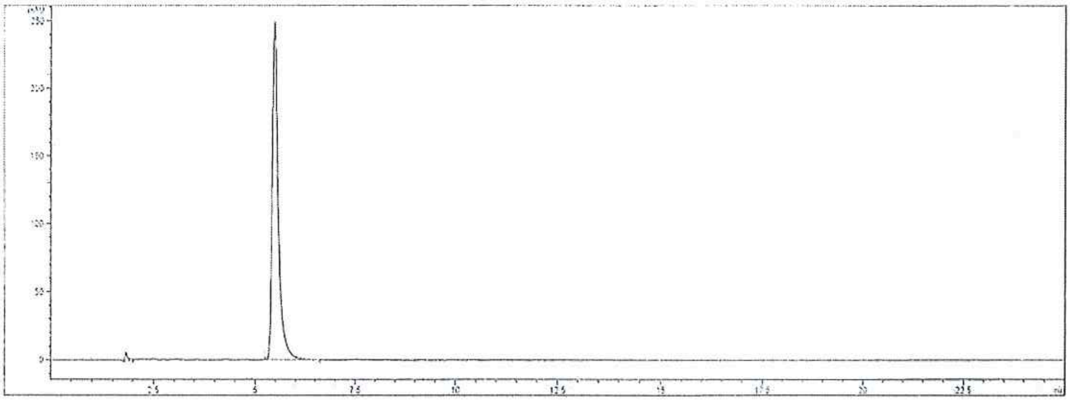

Compound	Retention time	Area%
MF3	5.48	>98

MF4

Gradient Shimming

Sample Name:
MF4
Data Collected on:
m300-mercury300
Archive directory:
/export/home/chempack/vnmrsys/data
Sample directory:

FidFile: PROTON

Pulse Sequence: PROTON (s2pul)
Solvent: cdcl3
Data collected on: Mar 17 2022

Operator: caccia

Relax. delay 1.000 sec
Pulse 45.0 degrees
Acq. time 1.706 sec
Width 4803.1 Hz
32 repetitions
OBSERVE H1, 300.1976543 MHz
DATA PROCESSING
FT size 16384
Total time 1 min 29 sec

Agilent Technologies

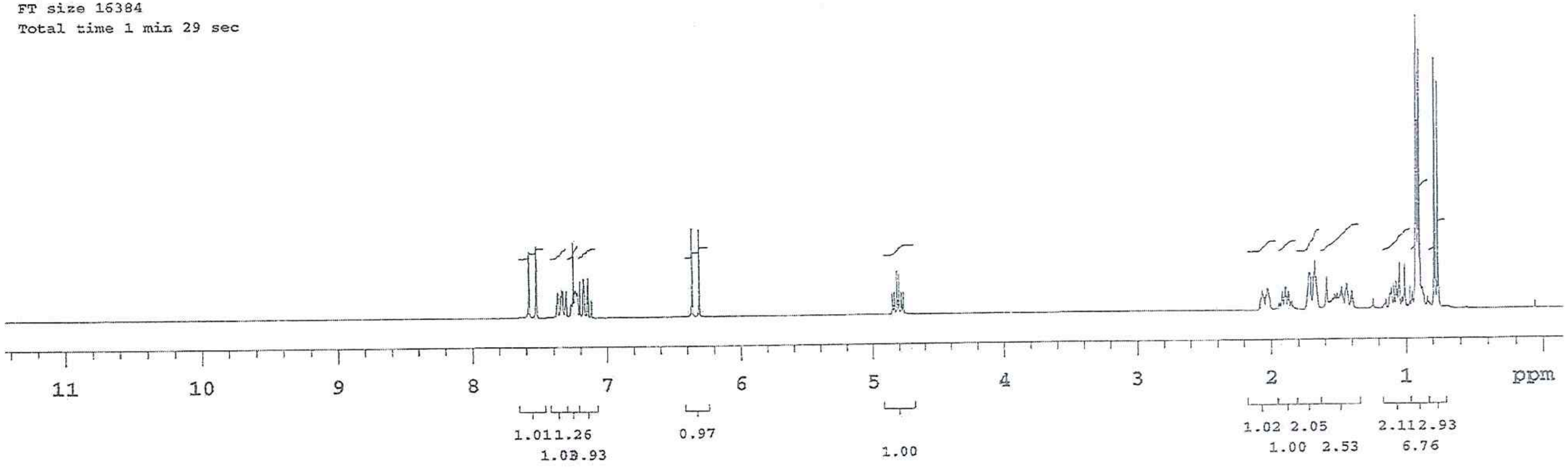

Agilent Technologies

Sample Name:
MF4_9-31
Data Collected on:
m300-mercury300
Archive directory:
/home/caccia/vnmrsys/data
Sample directory:
AutoSW_calib_20210712_02
FidFile: CARBON

Pulse Sequence: CARBON (s2pul)
Solvent: cdcl3
Data collected on: Jul 15 2021

Operator: caccia

Relax. delay 1.000 sec
Pulse 45.0 degrees
Acq. time 0.868 sec
Width 18867.9 Hz
2000 repetitions
OBSERVE C13, 75.4847602 MHz
DECOUPLE H1, 300.1991980 MHz
Power 38 dB
continuously on
WALTZ-16 modulated
DATA PROCESSING
Line broadening 0.5 Hz
FT size 32768
Total time 1 hr, 4 min

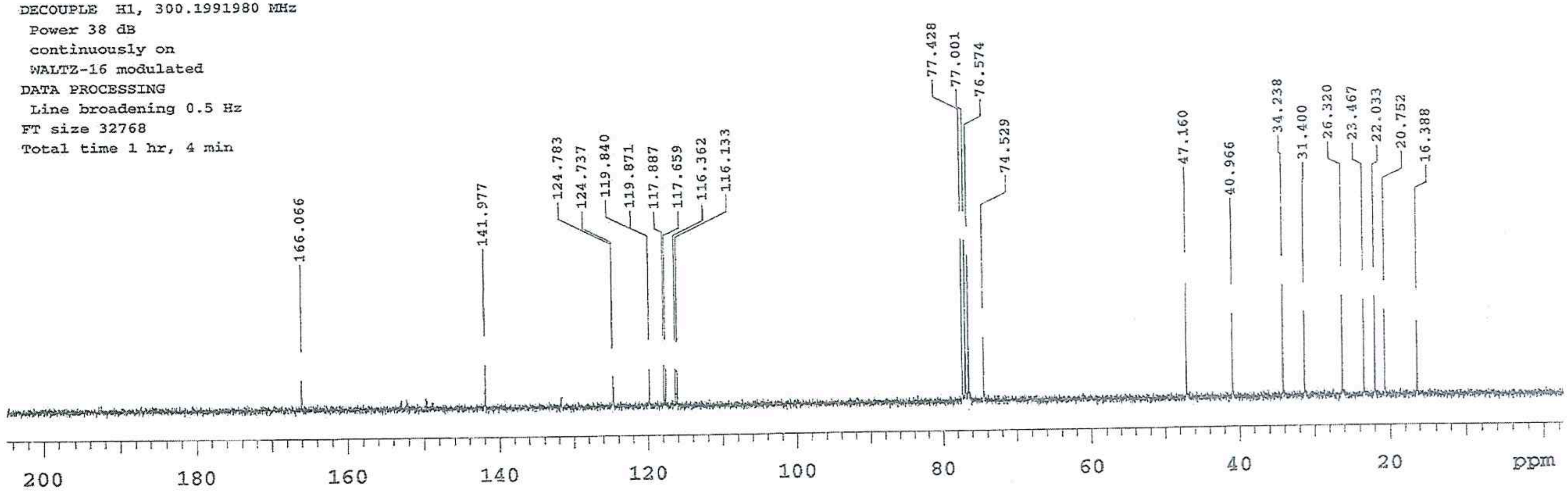

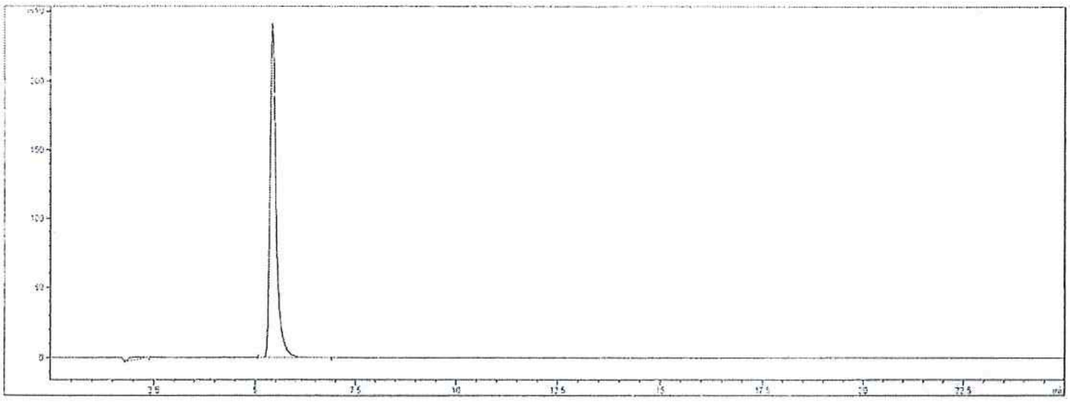

Compound	Retention time	Area%
MF4	5.41	>98

MCe1

Sample Name:
MC1_25-49
Data Collected on:
m300-mercury300
Archive directory:
/home/caccia/vnmrsys/data
Sample directory:
AutoSW_calib_20210712_02
FidFile: PROTON

Pulse Sequence: PROTON (s2pul)
Solvent: cdcl3
Data collected on: Jul 16 2021

Operator: caccia

Relax. delay 1.000 sec
Pulse 45.0 degrees
Acq. time 1.706 sec
Width 4803.1 Hz
32 repetitions
OBSERVE H1, 300.1976543 MHz
DATA PROCESSING
FT size 16384
Total time 1 min 29 sec

Agilent Technologies

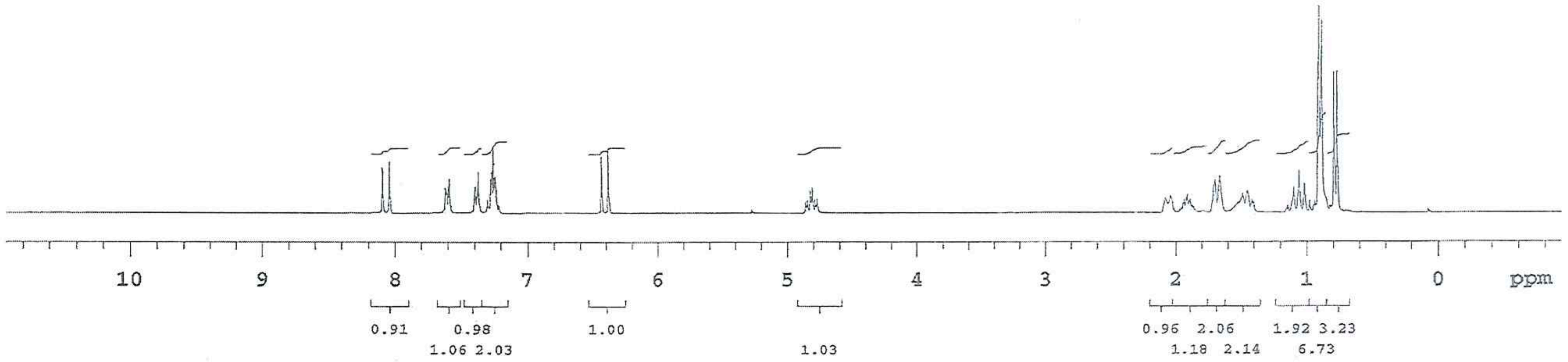

MCl

Agilent Technologies

Sample Name:
MC1_25-49
Data Collected on:
m300-mercury300
Archive directory:
/home/caccia/vnmrsys/data
Sample directory:
AutoSW_calib_20210712_02
FidFile: CARBON

Pulse Sequence: CARBON (s2pul)
Solvent: cdcl3
Data collected on: Jul 16 2021

Operator: caccia

Relax. delay 1.000 sec
Pulse 45.0 degrees
Acq. time 0.868 sec
Width 18867.9 Hz
2000 repetitions
OBSERVE C13, 75.4847602 MHz
DECOUPLE H1, 300.1991980 MHz
Power 38 dB
continuously on
WALTZ-16 modulated
DATA PROCESSING
Line broadening 0.5 Hz
FT size 32768
Total time 1 hr, 4 min

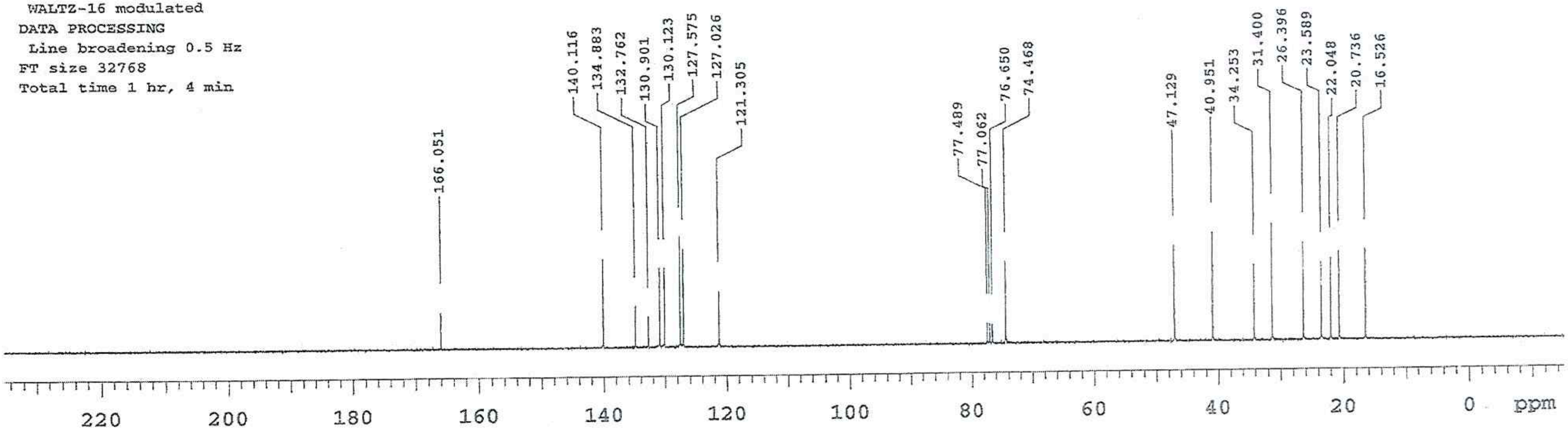

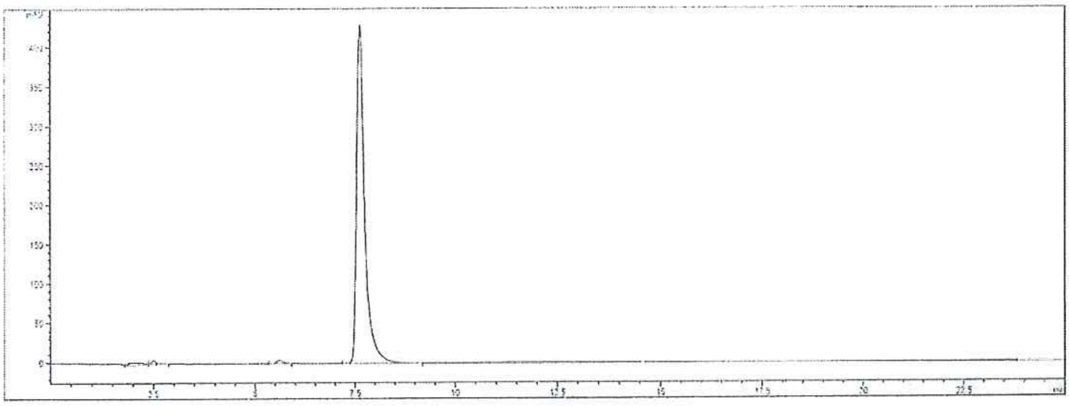

Compound	Retention time	Area%
MCI1	7.63	>97

MCI1 (320.15)

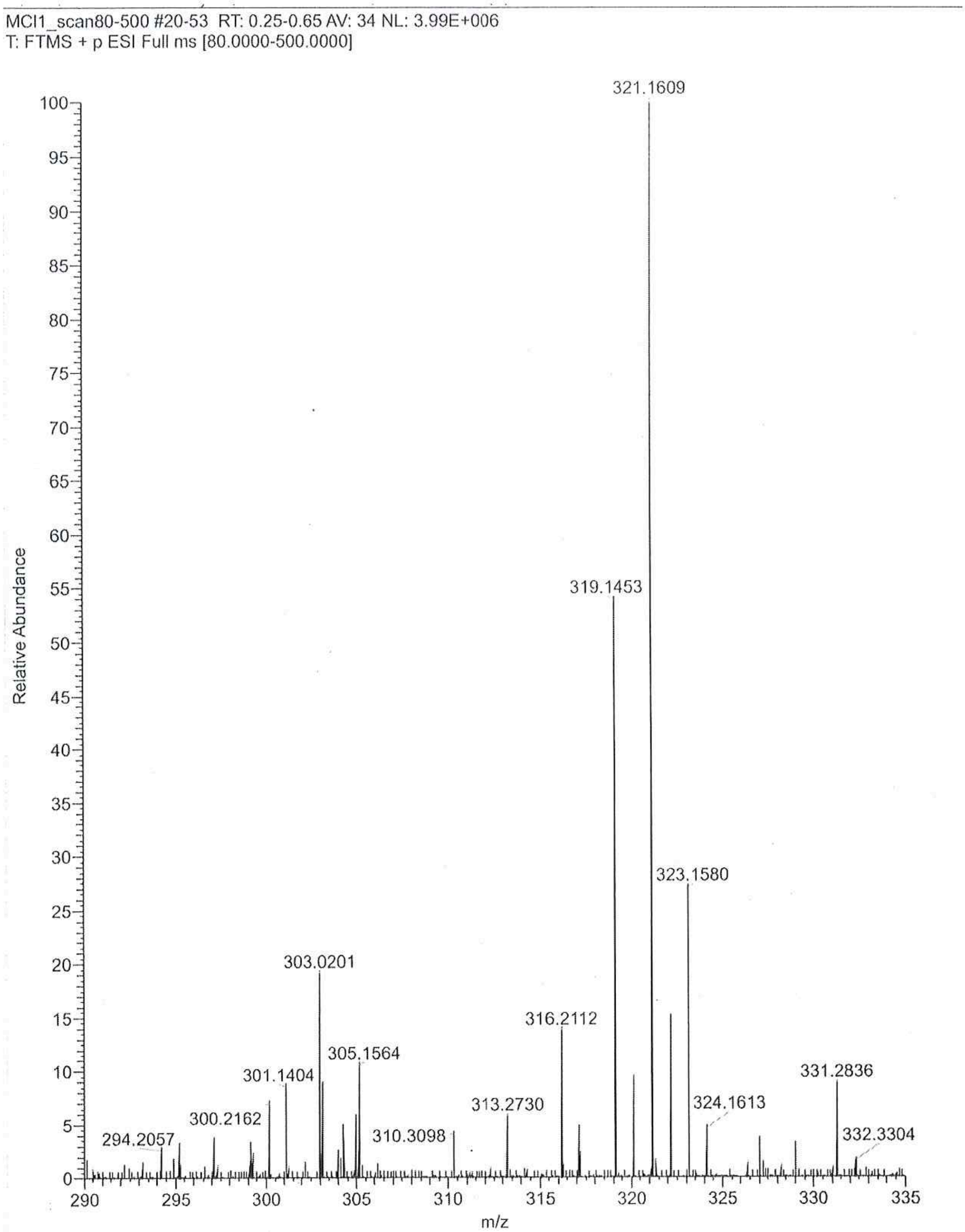
MCI1_scan80-500 #20-53 RT: 0.25-0.65 AV: 34 NL: 3.99E+006
T: FTMS + p ESI Full ms [80.0000-500.0000]
321.1609
319.1453
323.1580
303.0201
316.2112
305.1564
301.1404
331.2836
313.2730
324.1613
300.2162
310.3098
332.3304
294.2057
Relative Abundance
m/z
0
5
10
15
20
25
30
35
40
45
50
55
60
65
70
75
80
85
90
95
100
290
295
300
305
310
315
320
325
330
335

HCl2

Gradient Shimming

Sample Name:
MC12_21-53
Data Collected on:
m300-mercury300
Archive directory:
/export/home/chempack/vnmrsys/data
Sample directory:

FidFile: PROTON

Pulse Sequence: PROTON (s2pul)
Solvent: cdcl3
Data collected on: Sep 8 2021

Operator: caccia

Relax. delay 1.000 sec
Pulse 45.0 degrees
Acq. time 1.706 sec
Width 4803.1 Hz
64 repetitions
OBSERVE H1, 300.1976543 MHz
DATA PROCESSING
FT size 16384
Total time 2 min 58 sec

Agilent Technologies

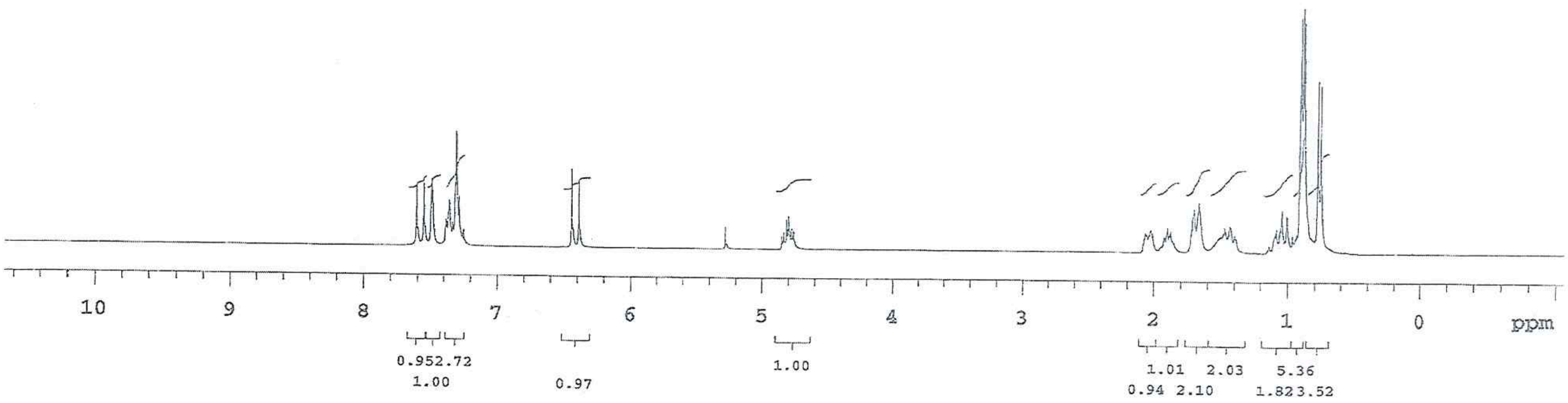

MCe2

Gradient Shimming

Sample Name:
MC12_21-53
Data Collected on:
m300-mercury300
Archive directory:
/export/home/chempack/vnmrsys/data
Sample directory:

FidFile: CARBON

Pulse Sequence: CARBON (s2pul)
Solvent: cdcl3
Data collected on: Sep 8 2021

Operator: caccia

Relax. delay 1.000 sec
Pulse 45.0 degrees
Acq. time 0.868 sec
Width 18867.9 Hz
2000 repetitions
OBSERVE C13, 75.4847602 MHz
DECOUPLE H1, 300.1991980 MHz
Power 38 dB
continuously on
WALTZ-16 modulated
DATA PROCESSING
Line broadening 0.5 Hz
FT size 32768
Total time 1 hr, 4 min

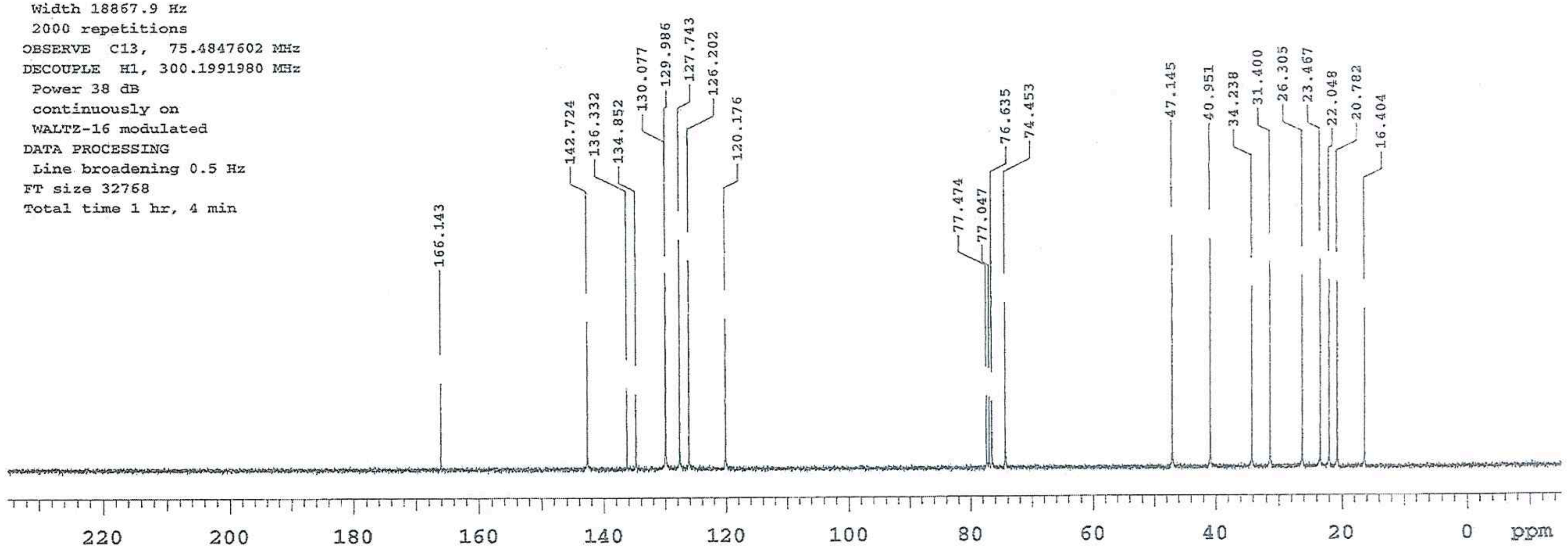

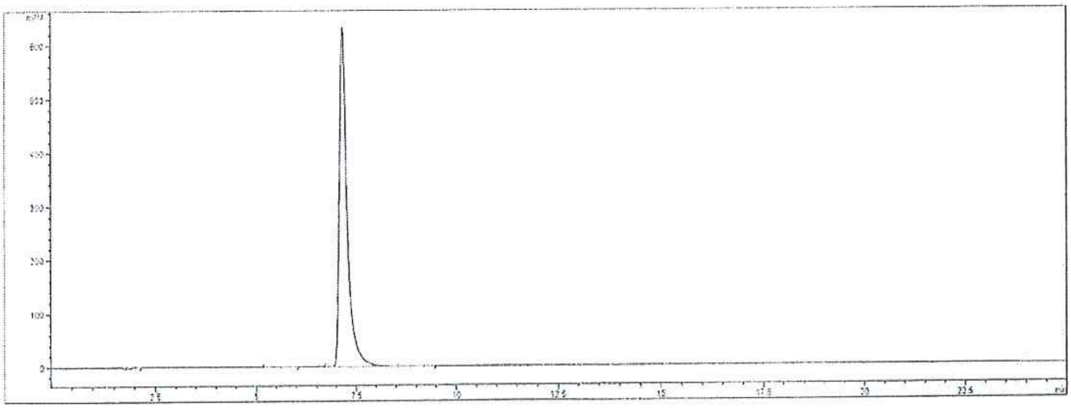

Compound	Retention time	Area%
MCl2	7.17	>99

MCl2 (320.15)

MCl2 #21-50 RT: 0.25-0.65 AV: 30 NL: 3.46E+006
T: FTMS + p ESI Full ms [80.0000-500.0000]

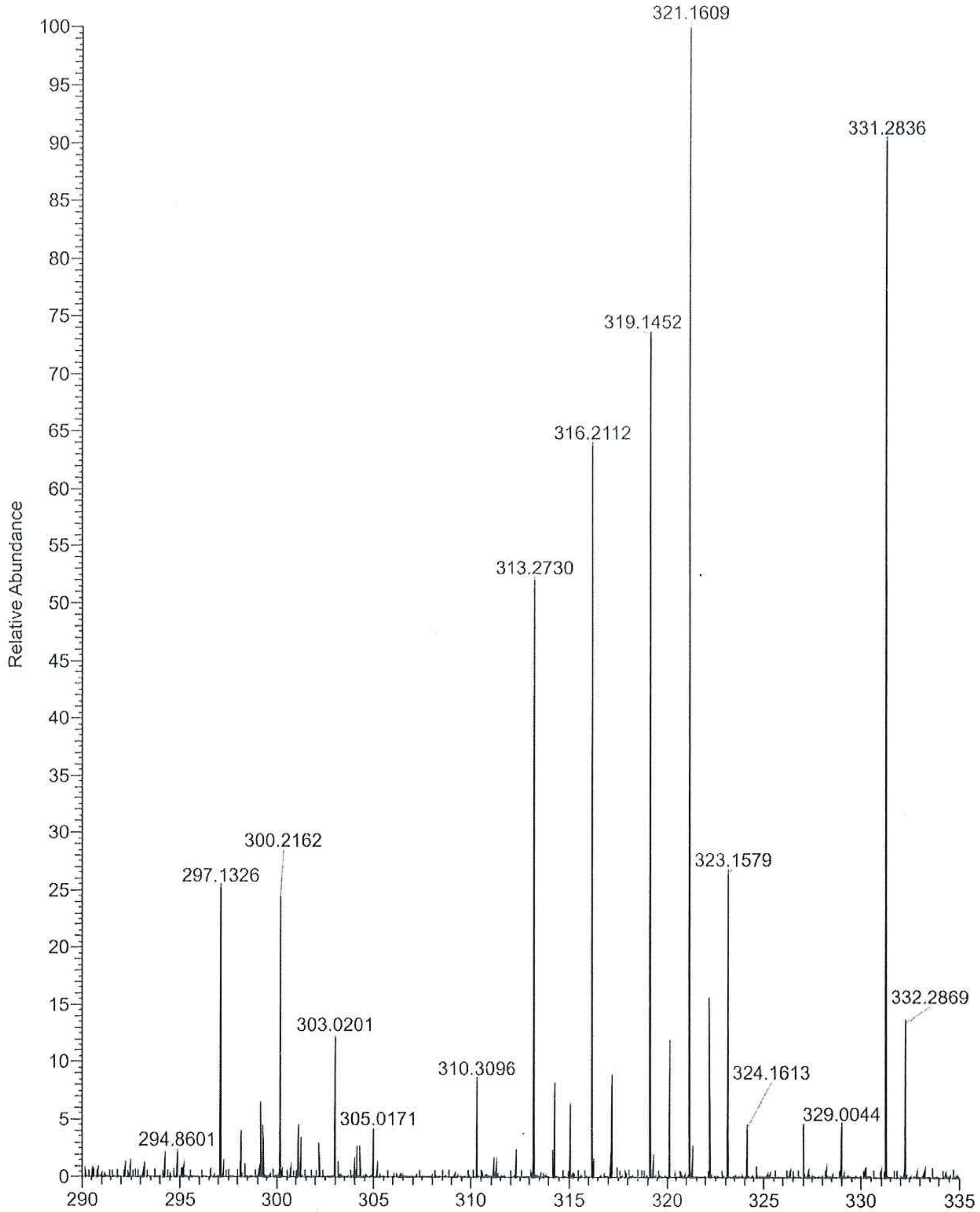

MCl3

Gradient Shimming

Sample Name:
MC13_19-29
Data Collected on:
m300-mercury300
Archive directory:
/export/home/chempack/vnmrsys/data
Sample directory:

FidFile: PROTON

Pulse Sequence: PROTON (s2pul)
Solvent: cdcl3
Data collected on: Sep 13 2021

Operator: caccia

Relax. delay 1.000 sec
Pulse 45.0 degrees
Acq. time 1.706 sec
Width 4803.1 Hz
64 repetitions
OBSERVE H1, 300.1976543 MHz
DATA PROCESSING
FT size 16384
Total time 2 min 58 sec

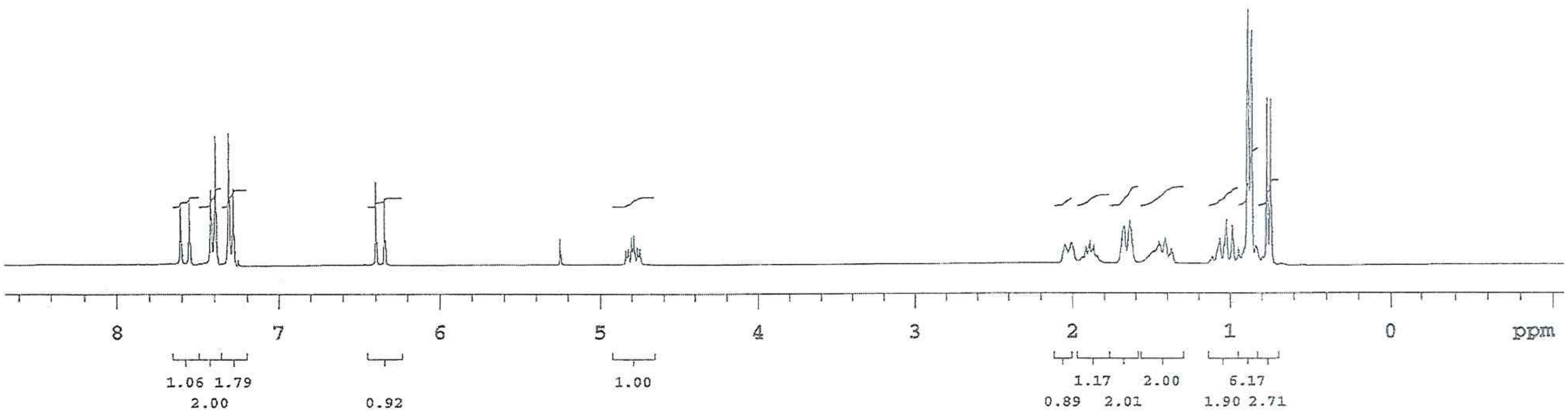

MCl3

Gradient Shimming

Sample Name:
MC13_19-29
Data Collected on:
m300-mercury300
Archive directory:
/export/home/chempack/vnmrsys/data
Sample directory:

FidFile: CARBON

Pulse Sequence: CARBON (s2pul)
Solvent: cdcl3
Data collected on: Sep 13 2021

Operator: caccia

Relax. delay 1.000 sec
Pulse 45.0 degrees
Acq. time 0.868 sec
Width 18867.9 Hz
2000 repetitions
OBSERVE C13, 75.4847602 MHz
DECOUPLE H1, 300.1991980 MHz
Power 38 dB
continuously on
WALTZ-16 modulated
DATA PROCESSING
Line broadening 0.5 Hz
FT size 32768
Total time 1 hr, 4 min

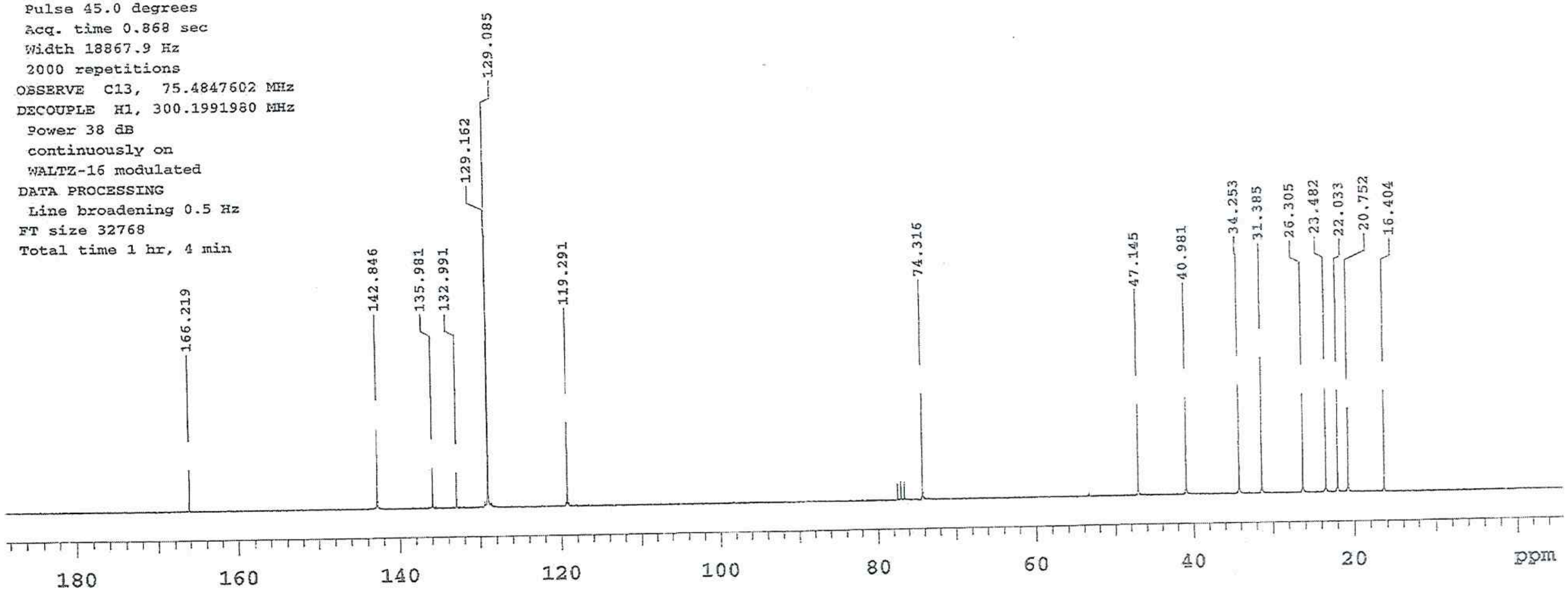

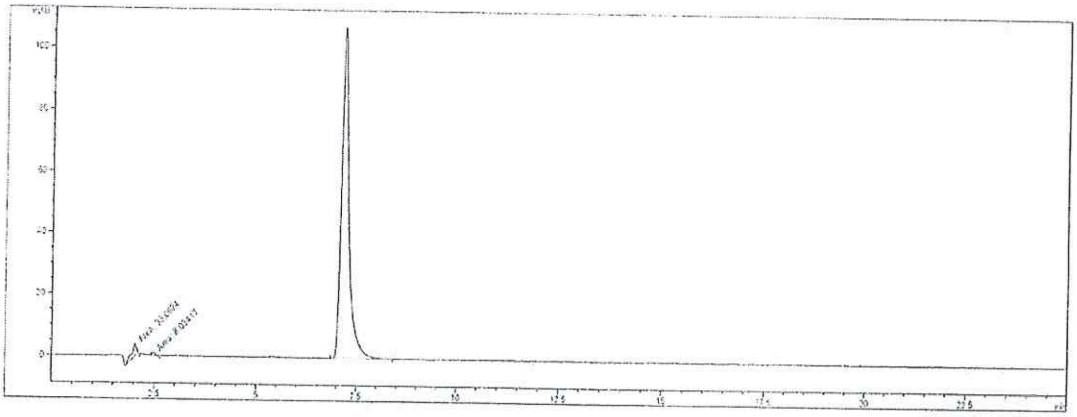

Compound	Retention time	Area%
MCl3	7.16	>97

MCl3 (320.15)

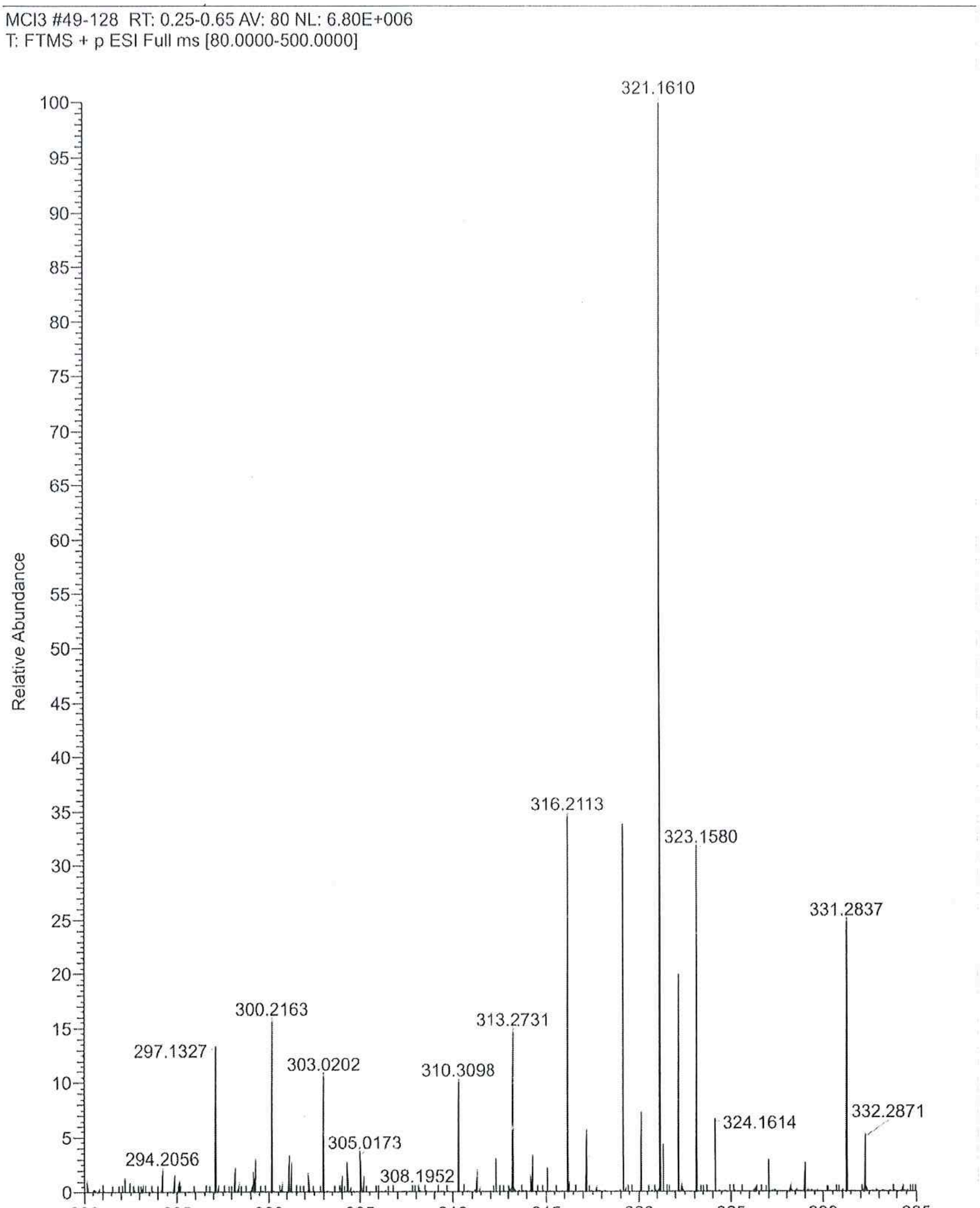
MCl3 #49-128 RT: 0.25-0.65 AV: 80 NL: 6.80E+006
T: FTMS + p ESI Full ms [80.0000-500.0000]
321.1610
316.2113
323.1580
331.2837
300.2163
313.2731
297.1327
303.0202
310.3098
332.2871
324.1614
305.0173
294.2056
308.1952
Relative Abundance
m/z

HQ4

Gradient Shimming

Sample Name:
MC14_fraz_7-35
Data Collected on:
m300-mercury300
Archive directory:
/export/home/chempack/vnmrsys/data
Sample directory:

FidFile: PROTON

Pulse Sequence: PROTON (s2pul)
Solvent: cdcl3
Data collected on: Sep 16 2021

Operator: caccia

Relax. delay 1.000 sec
Pulse 45.0 degrees
Acq. time 1.706 sec
Width 4803.1 Hz
64 repetitions
OBSERVE H1, 300.1976543 MHz
DATA PROCESSING
FT size 16384
Total time 2 min 58 sec

Agilent Technologies

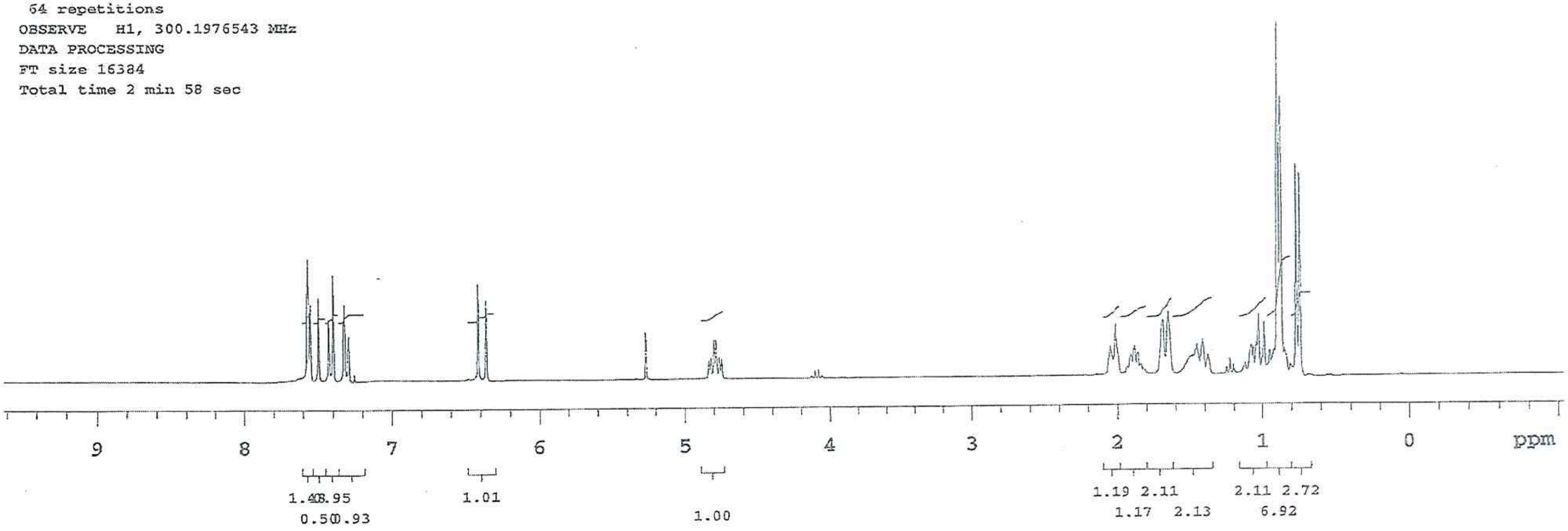

MC14

Agilent Technologies

Gradient Shimming

Sample Name:
MC14_fraz_7-35
Data Collected on:
m300-mercury300
Archive directory:
/export/home/chempack/vnmrsys/data
Sample directory:

FidFile: CARBON

Pulse Sequence: CARBON (s2pul)
Solvent: cdcl3
Data collected on: Sep 16 2021

Operator: caccia

Relax. delay 1.000 sec
Pulse 45.0 degrees
Acq. time 0.868 sec
Width 18867.9 Hz
2000 repetitions
OBSERVE C13, 75.4847602 MHz
DECOUPLE H1, 300.1991980 MHz
Power 38 dB
continuously on
WALTZ-16 modulated
DATA PROCESSING
Line broadening 0.5 Hz
FT size 32768
Total time 1 hr, 4 min

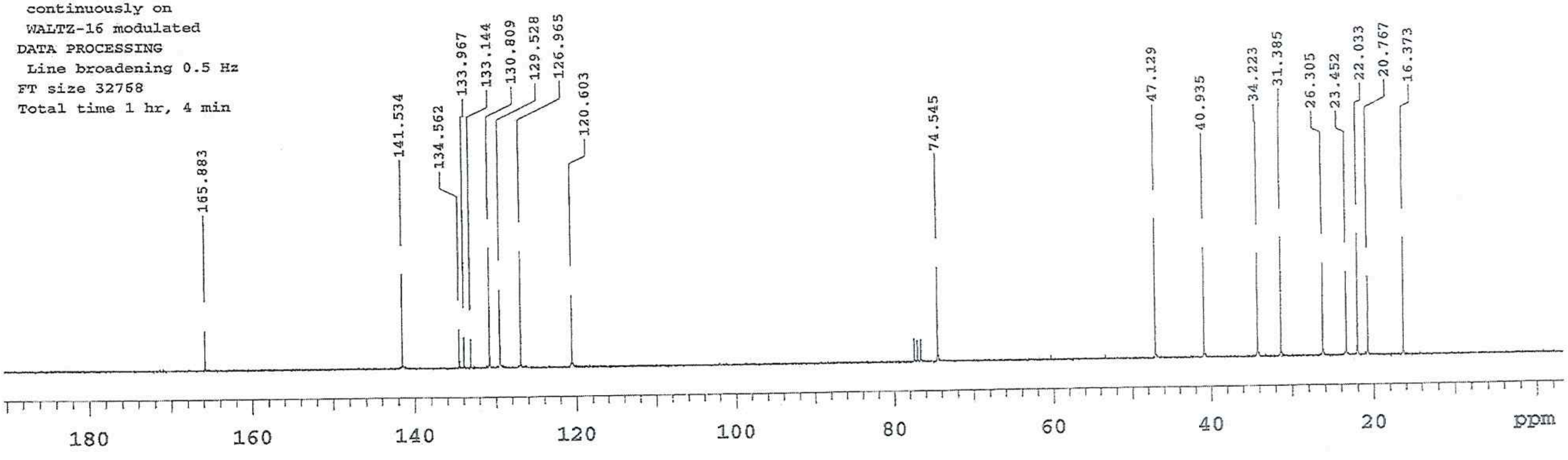

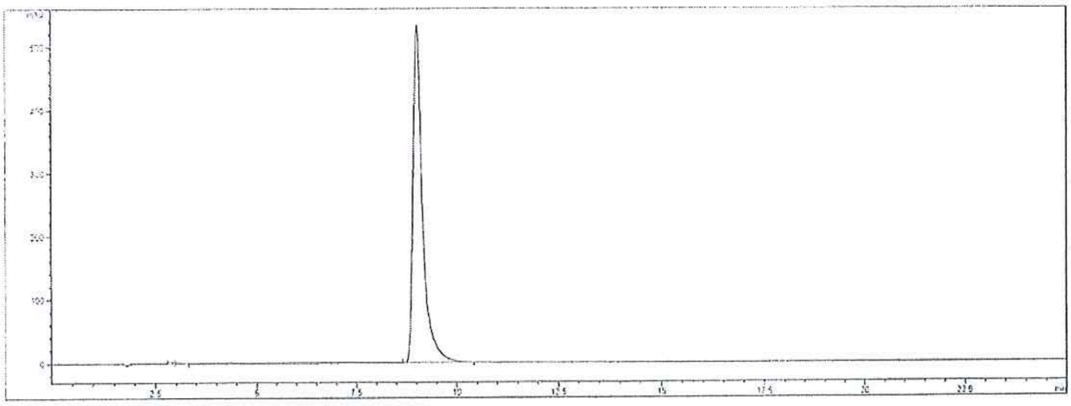

Compound	Retention time	Area%
MCl4	9.02	>99

MR-1

Agilent Technologies

Sample Name:
MBr1
Data Collected on:
m300-mercury300
Archive directory:

Sample directory:

FidFile: PROTON

Pulse Sequence: PROTON (s2pul)
Solvent: cdcl3
Data collected on: Sep 15 2022

Operator: caccia

Relax. delay 1.000 sec
Pulse 45.0 degrees
Acq. time 1.706 sec
Width 4803.1 Hz
64 repetitions
OBSERVE H1, 300.1976543 MHz
DATA PROCESSING
FT size 16384
Total time 2 min 58 sec

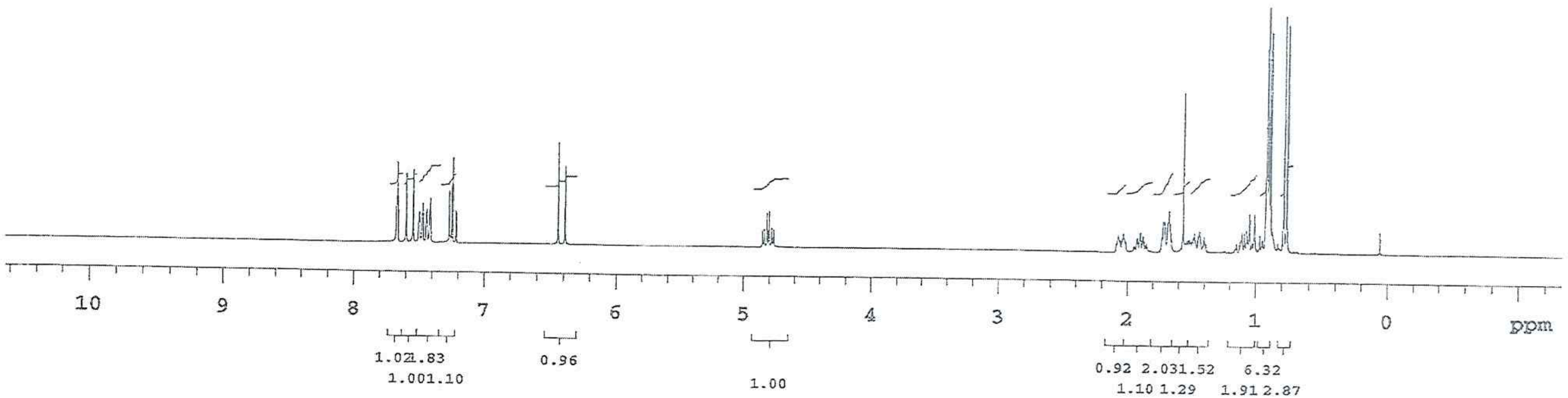

MCl4 (354.12)

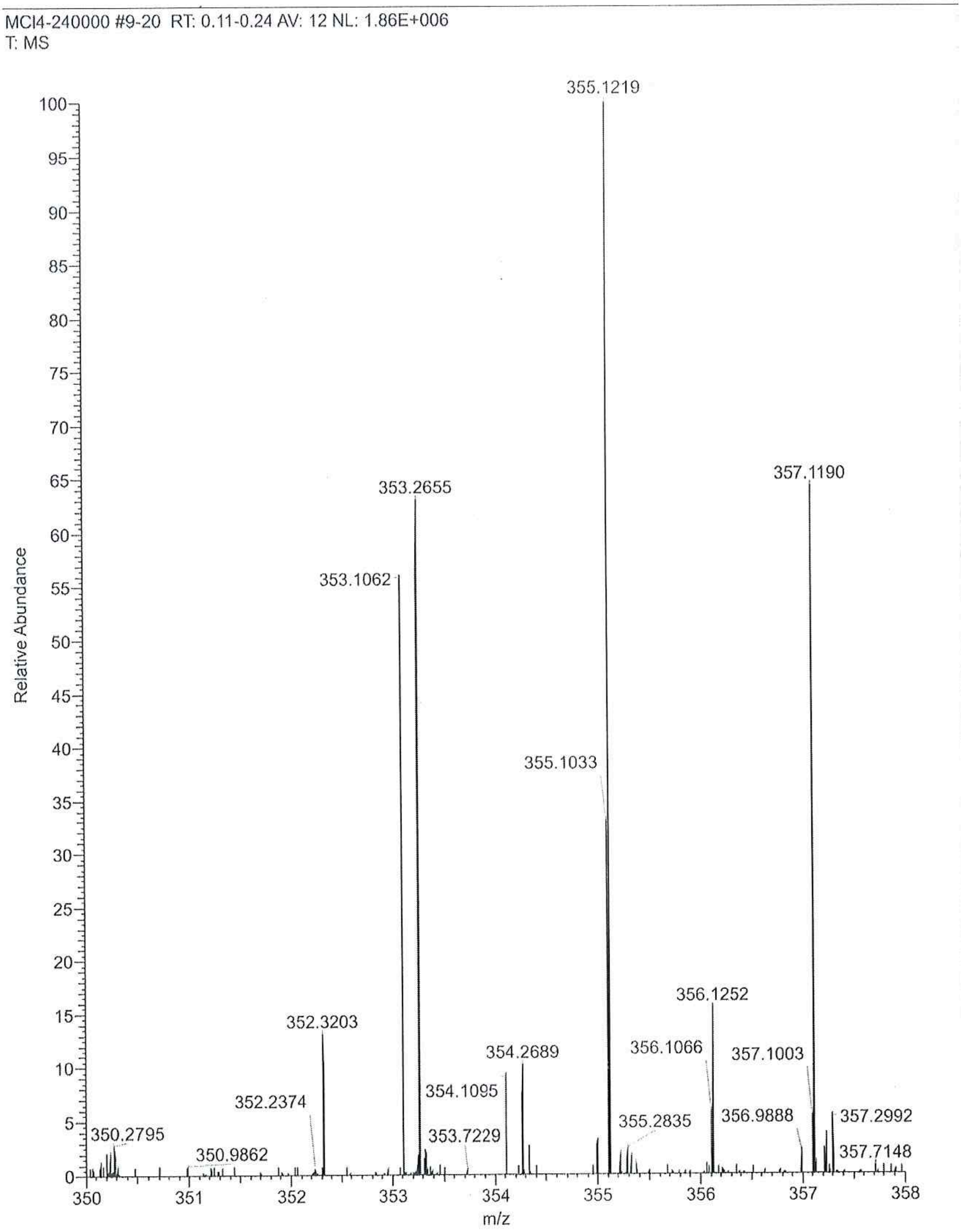
MCl4-240000 #9-20 RT: 0.11-0.24 AV: 12 NL: 1.86E+006
T: MS
Relative Abundance
m/z
355.1219
357.1190
353.2655
353.1062
355.1033
356.1252
352.3203
354.2689
356.1066
357.1003
354.1095
352.2374
356.9888
357.2992
355.2835
350.2795
353.7229
357.7148
350.9862

MB1

Gradient Shimming

Sample Name:
MBr.1
Data Collected on:
m300-mercury300
Archive directory:
/export/home/chempack/vnmrsys/data
Sample directory:

FidFile: CARBON

Pulse Sequence: CARBON (s2pul)
Solvent: cdcl3
Data collected on: Oct 20 2021

Operator: caccia

Relax. delay 1.000 sec
Pulse 45.0 degrees
Acq. time 0.868 sec
Width 18867.9 Hz
2000 repetitions
OBSERVE C13, 75.4847602 MHz
DECOUPLE H1, 300.1991980 MHz
Power 38 dB
continuously on
WALTZ-16 modulated
DATA PROCESSING
Line broadening 0.5 Hz
FT size 32768
Total time 1 hr, 4 min

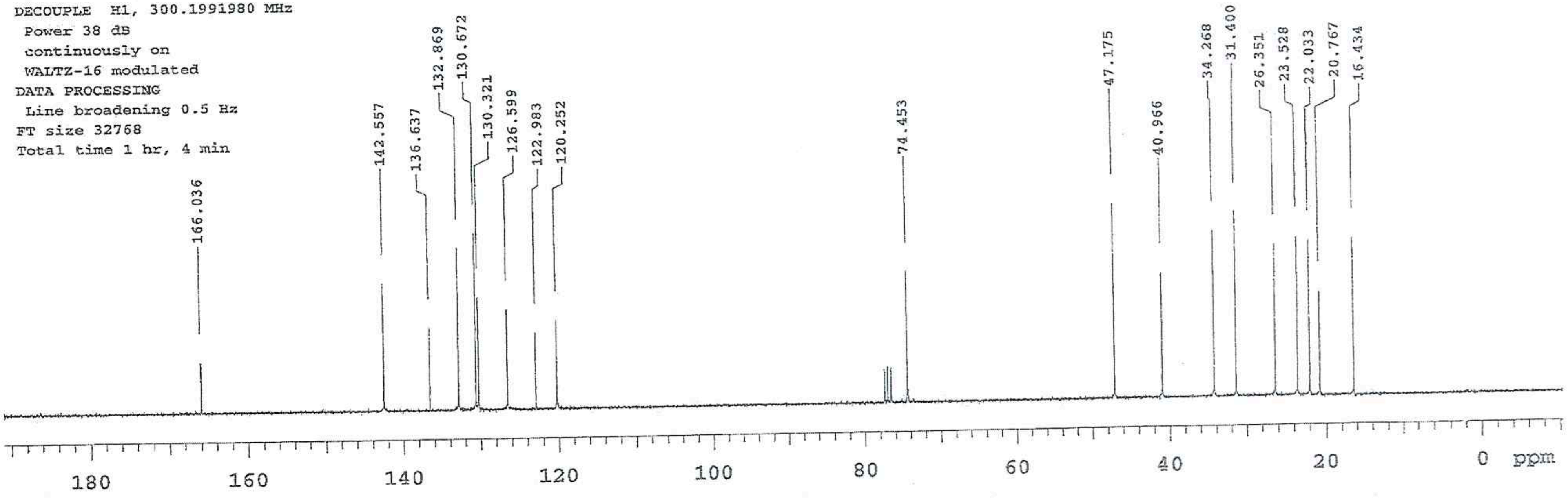

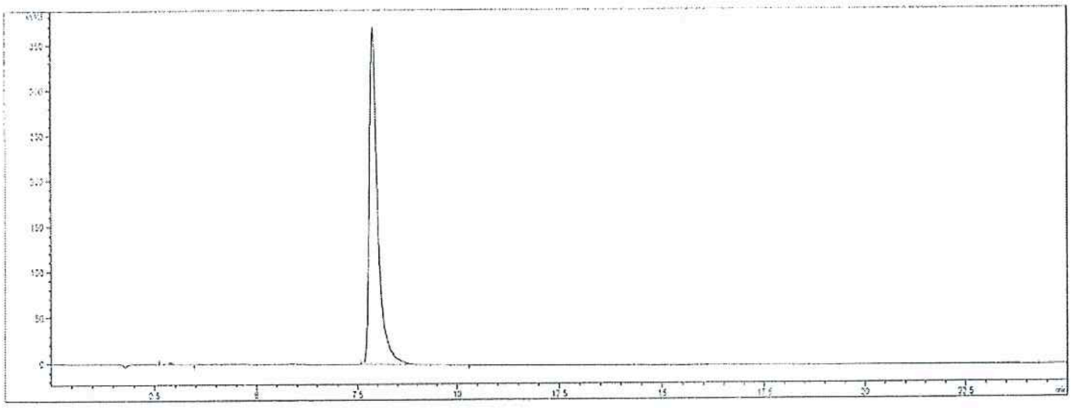

Compound	Retention time	Area%
MBr1	7.89	>99

MBr1 (364.10)

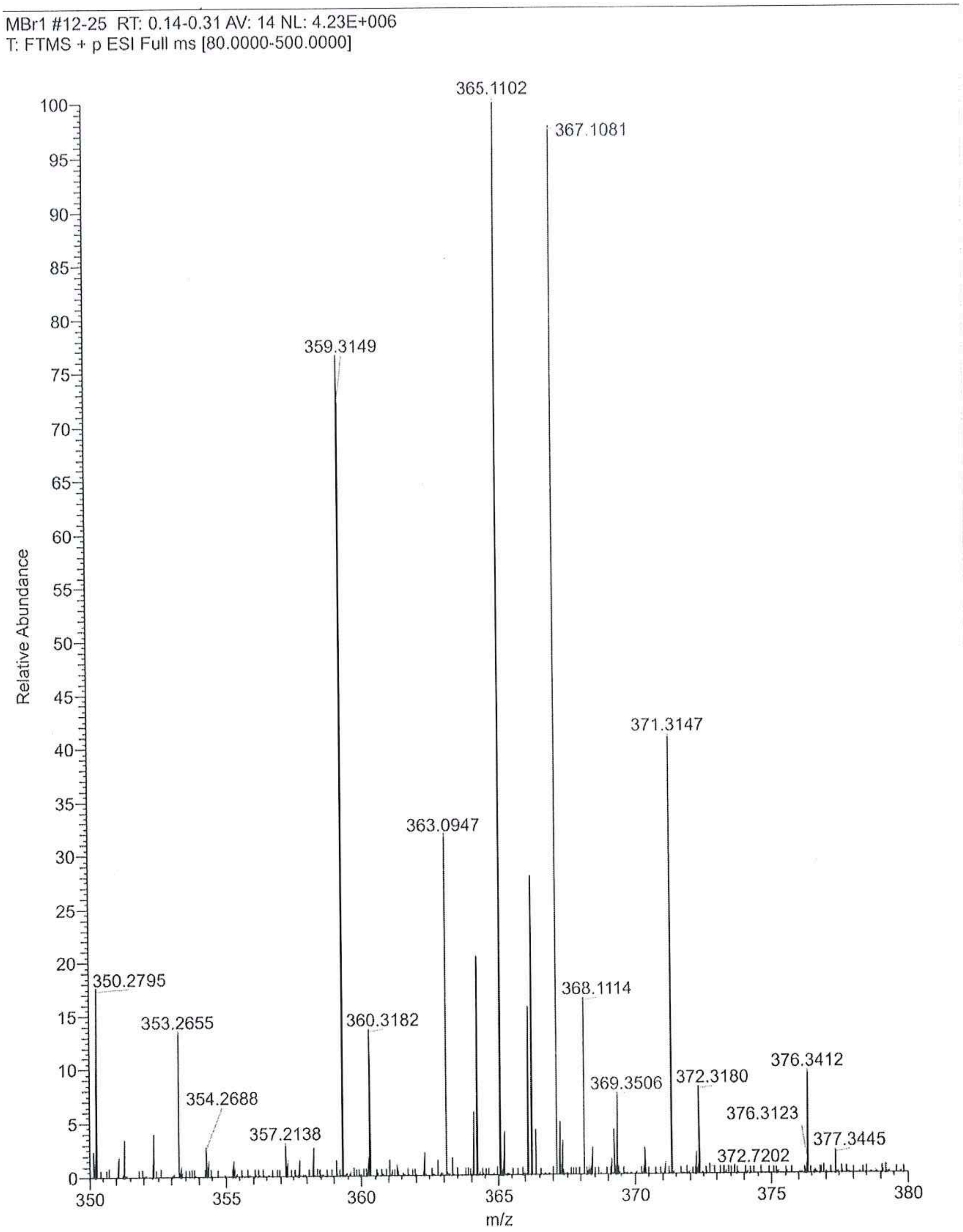
MBr1 #12-25 RT: 0.14-0.31 AV: 14 NL: 4.23E+006
T: FTMS + p ESI Full ms [80.0000-500.0000]
365.1102
367.1081
359.3149
371.3147
363.0947
350.2795
368.1114
353.2655
360.3182
376.3412
372.3180
369.3506
354.2688
376.3123
357.2138
377.3445
372.7202
Relative Abundance
m/z

MBr2

Agilent Technologies

Sample Name:
MBr2
Data Collected on:
m300-mercury300
Archive directory:

Sample directory:

FidFile: PROTON

Pulse Sequence: PROTON (s2pul)
Solvent: cdcl3
Data collected on: Sep 15 2022

Operator: caccia

Relax. delay 1.000 sec
Pulse 45.0 degrees
Acq. time 1.706 sec
Width 4803.1 Hz
64 repetitions
OBSERVE H1, 300.1976543 MHz
DATA PROCESSING
FT size 16384
Total time 2 min 58 sec

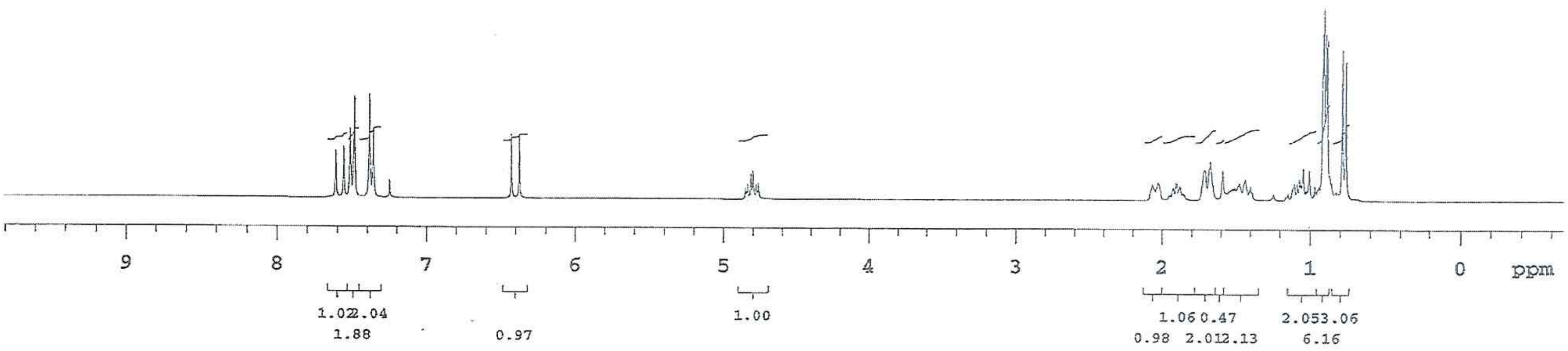

MBr2

Agilent Technologies

Sample Name:
MBr2-39-71
Data Collected on:
m300-mercury300
Archive directory:
/home/caccia/vnmrsys/data
Sample directory:
OLP-RA_MARI1_20211021_01
FidFile: CARBON

Pulse Sequence: CARBON (s2pul)
Solvent: cdcl3
Data collected on: Oct 26 2021

Operator: caccia

Relax. delay 1.000 sec
Pulse 45.0 degrees
Acq. time 0.868 sec
Width 18867.9 Hz
2000 repetitions
OBSERVE C13, 75.4847602 MHz
DECOUPLE H1, 300.1991980 MHz
Power 38 dB
continuously on
WALTZ-16 modulated
DATA PROCESSING
Line broadening 0.5 Hz
FT size 32768
Total time 1 hr, 4 min

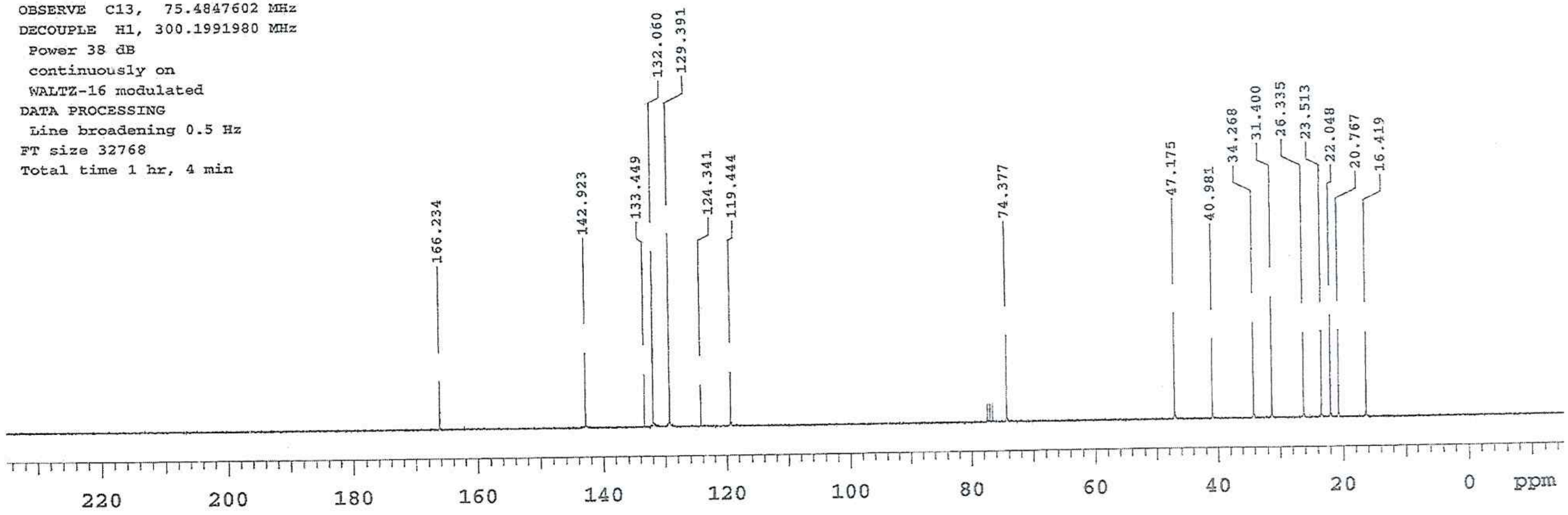

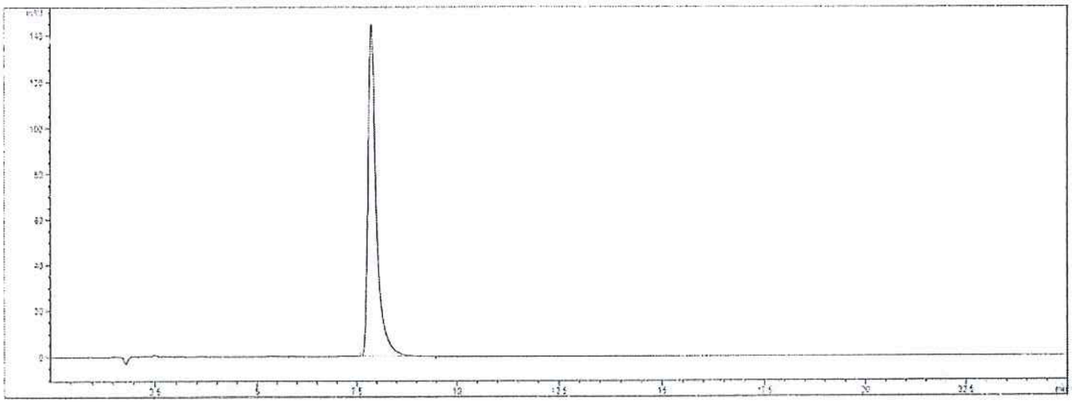

Compound	Retention time	Area%
MBr2	7.88	>99

MBr2 (364.10)

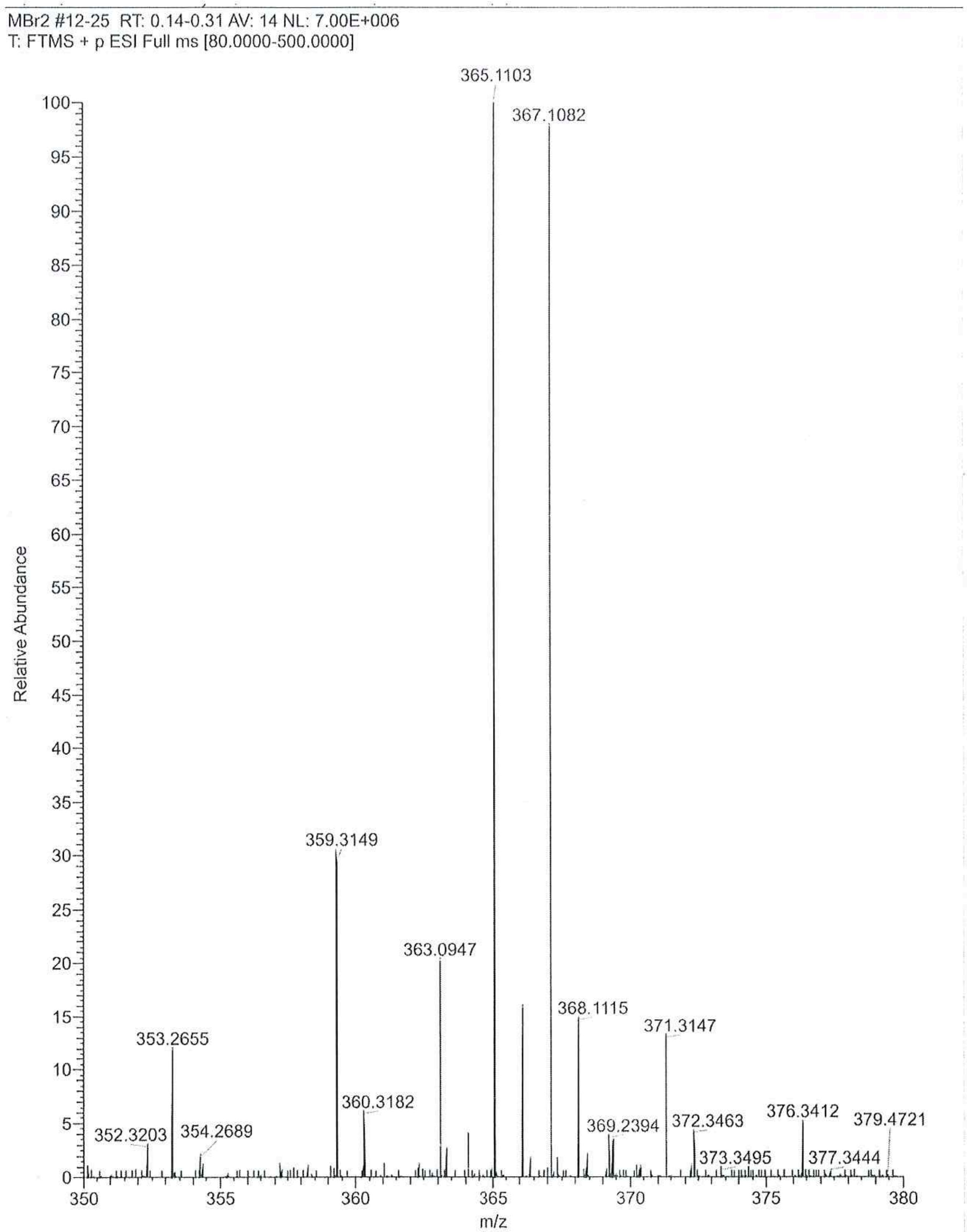
MBr2 #12-25 RT: 0.14-0.31 AV: 14 NL: 7.00E+006
T: FTMS + p ESI Full ms [80.0000-500.0000]
365.1103
367.1082
359.3149
363.0947
368.1115
371.3147
353.2655
360.3182
352.3203
354.2689
369.2394
372.3463
376.3412
379.4721
373.3495
377.3444
Relative Abundance
m/z
