# Supplementary material for: Advancing infection therapy: the role of novel menthol-based antimicrobials
Source: J Enzyme Inhib Med Chem. 2026 Jan 6;41(1):2596488. doi: 10.1080/14756366.2025.2596488 (PMC12777765; doi:10.1080/14756366.2025.2596488)
Supplement: Highlights JEIMC.docx [file IENZ_A_2596488_SM9673.docx]

Highlights

1. Menthol-based antimicrobials for the treatment of bacterial infections and related-biofilms.
2. MF1 showed antimicrobial activity against *E. faecium* infections and *S. aureus*-associated biofilm.
3. MCl2 promoted wound healing in human gingival fibroblasts.
