# Supplementary material for: Advancing infection therapy: the role of novel menthol-based antimicrobials
Source: J Enzyme Inhib Med Chem. 2026 Jan 6;41(1):2596488. doi: 10.1080/14756366.2025.2596488 (PMC12777765; doi:10.1080/14756366.2025.2596488)
Supplement: SI_Editable_Tables (1).docx [file IENZ_A_2596488_SM9672.docx]

| 1. **ADME STUDY**   **Table 1 SI.** Drug likeness properties^a^ | | | | |  |  |  |  |  |
| --- | --- | --- | --- | --- | --- | --- | --- | --- | --- |
| **Cpd** | **Water Solubility** | **Lipinski rule** | | | | **Lipinski Violations** | **TSPA** | **MR** | **Rb** |
|  |  | **MW** | **Log P** | **HBA** | **HBD** |  |  |  |  |
| **MC1** | 1.26*10^-2^ | 286.41 | 4.60 | 2 | 0 | 0 | 26.30 | 88.58 | 5 |
| **MC2** | 2.70*10^-2^ | 330.42 | 4.30 | 4 | 0 | 0 | 44.76 | 94.64 | 5 |
| **MC3** | 9.11*10^-3^ | 346.46 | 4.50 | 4 | 0 | 0 | 44.76 | 101.57 | 7 |
| **MC4** | 1.07*10^-2^ | 316.43 | 4.60 | 3 | 0 | 0 | 35.53 | 95.07 | 6 |
| **MC5** | 1.07*10^-2^ | 316.43 | 4.51 | 3 | 0 | 0 | 35.53 | 95.07 | 6 |
| **MC6** | 1.07*10^-2^ | 316.43 | 4.60 | 3 | 0 | 0 | 35.53 | 95.07 | 6 |
| **MC7** | 7.76*10^-3^ | 376.49 | 4.57 | 5 | 0 | 0 | 53.99 | 108.06 | 8 |
| **MBr1** | 2.51*10^-3^ | 365.30 | 5.08 | 2 | 0 | 1 | 26.30 | 96.28 | 5 |
| **MBr2** | 2.51*10^-3^ | 365.30 | 5.08 | 2 | 0 | 1 | 26.30 | 96.28 | 5 |
| **MF1** | 7.17*10^-3^ | 304.40 | 4.77 | 3 | 0 | 1 | 26.30 | 88.54 | 5 |
| **MF2** | 7.17*10^-3^ | 304.40 | 4.86 | 3 | 0 | 1 | 26.30 | 88.54 | 5 |
| **MF3** | 7.17*10^-3^ | 304.40 | 4.86 | 3 | 0 | 1 | 26.30 | 88.54 | 5 |
| **MF4** | 4.08*10^-3^ | 322.39 | 5.08 | 4 | 0 | 1 | 26.30 | 88.50 | 5 |
| **MCl1** | 3.55*10^-3^ | 320.85 | 4.98 | 2 | 0 | 1 | 26.30 | 93.59 | 5 |
| **MCl2** | 3.55*10^-3^ | 320.85 | 5.08 | 2 | 0 | 1 | 26.30 | 93.59 | 5 |
| **MCl3** | 3.55*10^-3^ | 320.85 | 5.08 | 2 | 0 | 1 | 26.30 | 93.59 | 5 |
| **MCl4** | 9.99*10^-4^ | 355.30 | 5.52 | 2 | 0 | 1 | 26.30 | 98.60 | 5 |
| ^a^ Prediction SwissADME platforms. | | | | |  |  |  |  |  |

1. **BIOLOGICAL STUDIES**

| **Table 2 SI.** Antimicrobial activity of menthol-based antimicrobials against Gram-positive and Gram-negative species. | | | | | | | | | |
| --- | --- | --- | --- | --- | --- | --- | --- | --- | --- |
| **Strain (n) ^a^** | **MIC (mg/L) ^b^** | **Menthol** | **MC1** | **MC2** | **MC3** | **MC4** | **MC5** | **MC6** | **MC7** |
| *S. aureus* | range | >512 | >512 | >512 | >512 | >512 | >512 | >512 | >512 |
| (10) | 50% | >512 | >512 | >512 | >512 | >512 | >512 | >512 | >512 |
|  | 90% | >512 | >512 | >512 | >512 | >512 | >512 | >512 | >512 |
| *S. epidermidis* | range | >512 | >512 | 512->512 | >512 | 512->512 | 512->512 | 512->512 | >512 |
| (8) | 50% | >512 | >512 | 512 | >512 | 512 | 512 | 512 | >512 |
|  | 90% | >512 | >512 | >512 | >512 | >512 | >512 | >512 | >512 |
| *E. faecalis* | range | >512 | 512->512 | >512 | >512 | 512->512 | >512 | >512 | >512 |
| (8) | 50% | >512 | 512 | >512 | >512 | 512 | >512 | >512 | >512 |
|  | 90% | >512 | >512 | >512 | >512 | >512 | >512 | >512 | >512 |
| *E. faecium* | range | >512 | 64->512 | 16-512 | 512 | 16-512 | 64->512 | 128->512 | 512 |
| (10) | 50% | >512 | 512 | 256 | 512 | 256 | 256 | 512 | 512 |
|  | 90% | >512 | >512 | 512 | 512 | 512 | 512 | 512 | 512 |
| *E. coli* | range | >512 | >512 | >512 | >512 | >512 | >512 | >512 | >512 |
| (8) | 50% | >512 | >512 | >512 | >512 | >512 | >512 | >512 | >512 |
|  | 90% | >512 | >512 | >512 | >512 | >512 | >512 | >512 | >512 |
| *K. pneumoniae* | range | >512 | >512 | >512 | >512 | >512 | >512 | >512 | >512 |
| (8) | 50% | >512 | >512 | >512 | >512 | >512 | >512 | >512 | >512 |
|  | 90% | >512 | >512 | >512 | >512 | >512 | >512 | >512 | >512 |
| *P. aeruginosa* | range | >512 | >512 | >512 | >512 | >512 | >512 | >512 | >512 |
| (8) | 50% | >512 | >512 | >512 | >512 | >512 | >512 | >512 | >512 |
|  | 90% | >512 | >512 | >512 | >512 | >512 | >512 | >512 | >512 |
| *A. baumannii* | range | 512->512 | >512 | >512 | >512 | >512 | >512 | >512 | >512 |
| (10) | 50% | 512 | >512 | >512 | >512 | >512 | >512 | >512 | >512 |
|  | 90% | >512 | >512 | >512 | >512 | >512 | >512 | >512 | >512 |
| *Enterobacter* spp. | range | >512 | >512 | >512 | >512 | >512 | >512 | >512 | >512 |
| (10) | 50% | >512 | >512 | >512 | >512 | >512 | >512 | >512 | >512 |
|  | 90% | >512 | >512 | >512 | >512 | >512 | >512 | >512 | >512 |
| **^a^** Including ATCC reference strains (n. 10); **^b^** 50% and 90%, MICs at which 50% and 90% of isolates are inhibited, respectively. | | | | | | | | | |
|  | | | | | | | | | |

| **Table 3 SI.** Antimicrobial activity of halogenated menthol derivatives. | | | | | | | | | | | | |
| --- | --- | --- | --- | --- | --- | --- | --- | --- | --- | --- | --- | --- |
| **Strain (n)^a^** | **MIC (mg/L) ^b^** | **Menthol** | **MF1** | **MF2** | **MF3** | **MF4** | **MBr1** | **MBr2** | **MCl1** | **MCl2** | **MCl3** | **MCl4** |
| *S. aureus* | range | >512 | >512 | >512 | >512 | >512 | >512 | >512 | >512 | >512 | >512 | >512 |
| (10) | 50% | >512 | >512 | >512 | >512 | >512 | >512 | >512 | >512 | >512 | >512 | >512 |
|  | 90% | >512 | >512 | >512 | >512 | >512 | >512 | >512 | >512 | >512 | >512 | >512 |
| *S. epidermidis* | range | >512 | >512 | >512 | >512 | >512 | >512 | >512 | >512 | >512 | >512 | >512 |
| (8) | 50% | >512 | >512 | >512 | >512 | >512 | >512 | >512 | >512 | >512 | >512 | >512 |
|  | 90% | >512 | >512 | >512 | >512 | >512 | >512 | >512 | >512 | >512 | >512 | >512 |
| *E. faecalis* | range | >512 | 64->512 | 64->512 | 64->512 | 64->512 | 64->512 | 128->512 | 64->512 | 64->512 | 64->512 | 64->512 |
| (8) | 50% | >512 | >512 | >512 | >512 | >512 | 512 | 512 | 512 | >512 | >512 | >512 |
|  | 90% | >512 | >512 | >512 | >512 | >512 | >512 | >512 | >512 | >512 | >512 | >512 |
| *E. faecium* | range | >512 | 8-64 | 128-256 | 64-256 | 64-256 | 32-512 | 256-512 | 64-256 | 32-128 | 32-128 | 256-512 |
| (10) | 50% | >512 | 16 | 128 | 128 | 128 | 256 | 256 | 128 | 128 | 128 | 256 |
|  | 90% | >512 | 32 | 128 | 128 | 128 | 512 | 256 | 256 | 128 | 128 | 256 |
| *E. coli* | range | >512 | >512 | >512 | >512 | >512 | >512 | >512 | >512 | >512 | >512 | >512 |
| (8) | 50% | >512 | >512 | >512 | >512 | >512 | >512 | >512 | >512 | >512 | >512 | >512 |
|  | 90% | >512 | >512 | >512 | >512 | >512 | >512 | >512 | >512 | >512 | >512 | >512 |
| *K. pneumoniae* | range | >512 | >512 | >512 | >512 | >512 | >512 | >512 | >512 | >512 | >512 | >512 |
| (8) | 50% | >512 | >512 | >512 | >512 | >512 | >512 | >512 | >512 | >512 | >512 | >512 |
|  | 90% | >512 | >512 | >512 | >512 | >512 | >512 | >512 | >512 | >512 | >512 | >512 |
| *P. aeruginosa* | range | >512 | 512->512 | >512 | >512 | >512 | 512->512 | >512 | 512->512 | >512 | >512 | >512 |
| (8) | 50% | >512 | >512 | >512 | >512 | >512 | >512 | >512 | >512 | >512 | >512 | >512 |
|  | 90% | >512 | >512 | >512 | >512 | >512 | >512 | >512 | >512 | >512 | >512 | >512 |
| *A. baumannii* | range | 512->512 | 512->512 | 512->512 | 512->512 | 512->512 | 512->512 | >512 | 512->512 | 512->512 | 512->512 | 512->512 |
| (10) | 50% | 512 | 512 | 512 | 512 | 512 | 512 | >512 | 512 | 512 | 512 | 512 |
|  | 90% | >512 | >512 | >512 | >512 | >512 | >512 | >512 | >512 | >512 | >512 | >512 |
| *Enterobacter* spp. | range | >512 | >512 | >512 | >512 | >512 | >512 | >512 | >512 | >512 | >512 | >512 |
| (10) | 50% | >512 | >512 | >512 | >512 | >512 | >512 | >512 | >512 | >512 | >512 | >512 |
|  | 90% | >512 | >512 | >512 | >512 | >512 | >512 | >512 | >512 | >512 | >512 | >512 |
| ^a^Including ATCC reference strains (n. 10); ^b^50% and 90%, MICs at which 50% and 90% of isolates are inhibited, respectively. | | | | | | | | | | | | |

1. **COMPUTATIONAL STUDIES**

| **Table 4 SI.** Binding affinities in Kcal/mol. | | | |
| --- | --- | --- | --- |
| **MF1** | **MF2** | **MCl2** | **MCl3** |
| -6.70 | -7,35 | -8.43 | -8.21 |

| **Table 5 SI.** Average Energy and Temperature for **MF1**, **MF2**, **MCl2**, and **MCl3.** | | |
| --- | --- | --- |
| Average | Energy | Temperature |
| **MF1** | -782671 | 303.182 |
| **MF2** | -783064 | 303.181 |
| **MCl2** | -779848 | 303.181 |
| **MCl3** | -784730 | 303.182 |

**Molecular Mechanics (MM)/Generalized Born Surface Area (GSBA) analysis**

The MM/GBSA analysis aims to estimate the overall binding free energy of a biomolecular complex. The MM/GBSA results for the **MF1**, **MF2**, **MCl2**, and **MCl3** provided crucial insights into binding and solvation energies, which are vital for understanding the interaction between these molecules and a protein (Table 6 Supporting Information).

ΔVDWAALS (Van der Waals Energy): This component measures the interaction between protein and ligand atoms due to Van der Waals forces. A trend of decreasing ΔVDWAALS values is observed when moving from **MF1** to **MCl3**, suggesting that Van der Waals interactions become more favorable from **MF1** to **MCl3**.

ΔEEL (Electrostatic Energy): This component quantifies the electrostatic interaction between partial charges of protein and ligand atoms. MF1 exhibits a positive contribution to binding energy, indicating a favorable electrostatic interaction, while for MF2, MCl2, and MCl3, the values are negative, suggesting an unfavorable contribution to binding energy.

ΔEGB (Born Solvated Energy): This represents the solvation energy calculated using the Born model to estimate the solvent effect. ΔEGB values increase from MF1 to MCl3, indicating a progressively positive contribution to binding energy due to solvation.

ΔESURF (Surface Energy): This component accounts for interactions with the solvent associated with the protein and ligand surfaces. Values are relatively consistent across different molecules, indicating that surface energy has minimal impact on binding energy variations.

ΔGGAS (Gas Phase Free Energy): It is the sum of Van der Waals and electrostatic energies in the gas phase (without solvent). ΔGGAS values decrease from MF1 to MCl3, suggesting that gas-phase energy becomes more favorable.

ΔGSOLV (Solvation Energy): This is the sum of Born and surface solvation energies, representing the solvent's effect on binding energy. Values increase from MF1 to MCl3, indicating a progressively positive contribution due to solvation.

ΔTOTAL (Total Energy): It is the sum of all the previous components. Values are negative for all molecules, indicating that the total energy favors the formation of the bond between the protein and ligands. However, ΔTOTAL values are more negative for MCl2 and MCl3 compared to MF1 and MF2, suggesting that MCl2 and MCl3 have an overall more favorable binding energy, this is consistent with the affinities obtained through molecular docking.

| **Table 6 SI**. Energy components (Kcal/mol/protein-ligand) for **MF1**, **MF2**, **MCl2**, and **MCl3**. | | | | |
| --- | --- | --- | --- | --- |
| **Delta Energy (Kcal/mol)** | **MF1** | **MF2** | **MCl2** | **MCl3** |
| ΔVDWAALS | -29.69 | -32.71 | -35.94 | -37.28 |
| ΔEEL | 0.21 | -18.16 | -3.03 | -4.58 |
| ΔEGB | 12.88 | 29.94 | 17.45 | 20.41 |
| ΔESURF | -4.59 | -5.06 | -5.06 | -5.07 |
| ΔGGAS | -29.47 | -50.88 | -38.97 | -41.86 |
| ΔGSOLV | 8.28 | 24.88 | 12.39 | 15.34 |
| ΔTOTAL | -21.19 | -25.99 | -26.58 | -26.52 |
